# On-Surface Synthesis of Azobenzene-Linked Porphyrin Derivatives

**DOI:** 10.1021/acs.jpclett.5c03174

**Published:** 2025-11-06

**Authors:** Yuji Isshiki, Donglin Li, Saranyan Vijayaraghavan, Kewei Sun, Huynh Thien Ngo, Luiza Buimaga-Iarinca, Yoshitaka Matsushita, Edward A. Neal, Cristian Morari, Jonathan P. Hill, Shigeki Kawai

**Affiliations:** † Center for Basic Research on Materials, 52747 National Institute for Materials Science, 1-2-1 Sengen, Tsukuba, Ibaraki 305-0047, Japan; ‡ CSIR-Central Electrochemical Research Institute, Karaikudi 630003, India; § Research Center for Materials Nanoarchitectonics, 52747 National Institute for Materials Science, 1−1 Namiki, Tsukuba, Ibaraki 305-0044, Japan; ∥ National Institute for Research and Development of Isotopic and Molecular Technologies (NIRDIMT), 65-103 Donat, 400293 Cluj-Napoca, Romania; ⊥ Research Network and Facility Services Division, 52747 National Institute for Materials Science, 1-2-1 Sengen, Tsukuba, Ibaraki 305-0047, Japan; # Graduate School of Pure and Applied Sciences, University of Tsukuba, Tsukuba, Ibaraki 305-0047, Japan

## Abstract

On-surface synthesis has become an attractive strategy to obtain functionalized carbon nanostructures from small precursor molecules using a bottom-up approach. Although various on-surface reactions have been developed, it is still unclear how the chirality of self-assembled structures prior to reaction affects the coupling process. Here, we investigate homocoupling of nitro-phenyl groups in Pt-porphyrin derivatives on Au(111) surfaces using low-temperature scanning tunneling microscopy. Two different self-assembled structures composed either of linear oligomer of molecules of opposing chirality or of discrete trimers of molecules having the same chirality (i.e., homochiral) were respectively obtained by using differently substituted phenyl and 3,5-di-*tert*-butylphenyl groups porphyrin cores. A machine-learning-assisted protocol was used for large-scale statistical analysis revealing the distinct difference in reaction yields of azobenzene formation between two different self-assembled structures. Since the azobenzene (dimer) products are composed of two molecules with opposing chirality (i.e., they are heterochiral), the molecules in the homochiral assembly must first be disassembled, which is one of the reasons for the low reaction yield. This study highlights the significant role of porphyrin chirality in the azo coupling reaction process on surface.

## Graphical abstract

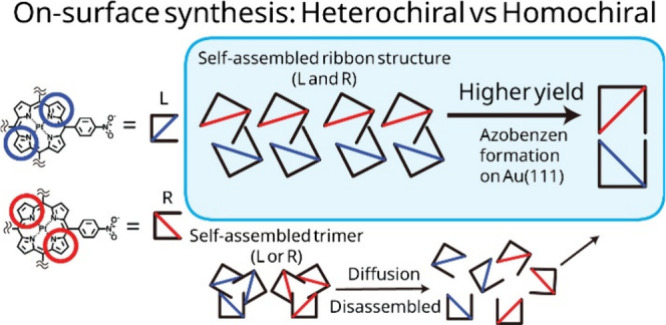

Porphyrins play important roles in a wide variety of natural and synthetic systems from biological catalysts to molecular electronic devices.^1–4^ Their ability to coordinate metals makes them essential components of catalytic^5^ or light-harvesting systems,^6^ and so on. In particular, π-extended porphyrin molecules are attracting attention in the field of reversible photoisomerization and photoresponsive conjugated molecules.^7–9^ To this end, various bonds between alkenes, alkynes, imines, and azo groups have been formed to extend the conjugated systems. Of these, azo groups have relatively strong electronic exchange with porphyrins^10^ due to orbital matching of porphyrins with azo bridges based on continuous sp^2^ conjugation and fewer steric effects (compared to, e.g., alkenes).

On-surface chemistry is a promising bottom-up approach to molecular systems assembly and synthesis and has, for example, enabled the creation of designer carbon nanostructures through the coupling of small organic molecules adsorbed on surfaces.^11^ In particular, since the first synthesis of graphene nanoribbons (GNR) in 2010,^12^ this field has developed rapidly. Numerous homogeneous reactions^13–15^ have been extensively studied on metal surfaces to construct functional nanostructures using carefully designed precursor molecules. Among the various precursor molecules, porphyrins stand out as particularly attractive building blocks due to their versatile coordination chemistry, which enables tuning of electronic, optical, and magnetic properties. To this end, significant advancements involving porphyrins have been achieved, including the synthesis of π-extended porphyrins,^16,17^ hierarchical extension in two-dimensions,^18^ porphyrin–GNR hybrids,^19^ block oligomers,^20,21^ and quantum nanomagnets.^22^ Beyond the construction of functional nanostructures, an understanding of the mechanisms of these reactions is also crucial, as they differ significantly from traditional wet chemistry reaction pathways. Reactions occurring at a surface are profoundly influenced by different surface effects, including catalytically active adatoms,^23^ surface mobility,^24^ and lattice-guided interactions.^25^ While self-assembled structures have been demonstrated to effectively modulate the activity and selectivity of surface reactions,^26,27^ reactions involving chirality at a surface remain relatively unexplored. It is anticipated that the unique assembly patterns of chiral molecules hold strong potential to steer reaction sites and influence reaction mechanisms.

Here, we present the on-surface homocoupling between nitrobenzene groups, which are connected to the *meso*-positions of porphyrin derivatives (Scheme 1). To investigate the influence of the relative position of the reactant, two different molecular assemblies were formed by substituting simple phenyl or 3,5-di-*tert*-butyl-phenyl groups to the porphyrins. Using scanning tunneling microscopy (STM), we have found that the initial self-assembled structure affects the efficiency of azobenzene formation. This research provides insights into the on-surface synthesis using chiral precursors.

**Scheme 1:**
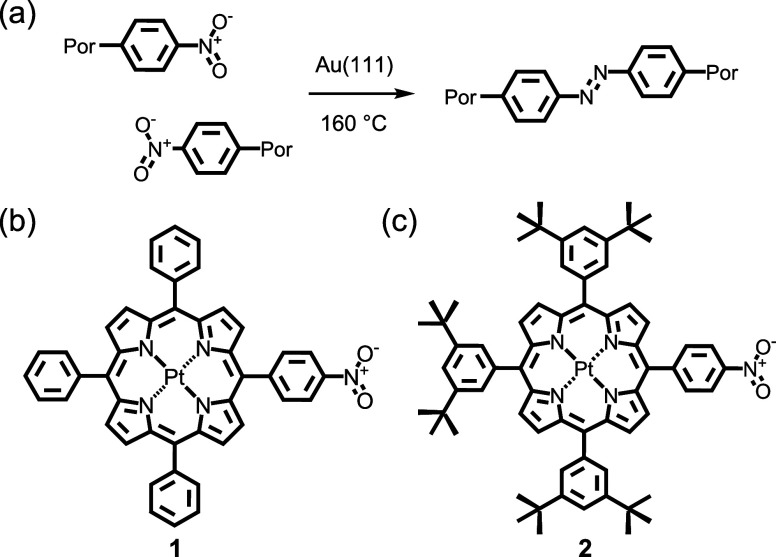
Synthesis and Structure. (**a**) Homocoupling between Nitro Groups Promoted by Annealing on the Au(111) Surface. Chemical Structures of (**b**) 5-(4-Nitrophenyl)-10,15,20-triphenylporphyrin platinum­(II) (**1**) and (**c**) 5-(4-Nitrophenyl)-10,15,20-tris­(3,5-di-t-butylphenyl)­porphyrin platinum­(II) (**2**)­^a^

a Platinum­(II) porphyrin complexes were selected for their excellent stabilities.

The 4-nitrophenyl-substituted compounds used in this work were selected for their known propensity to undergo reaction to form substituted azobenzene derivatives on Au(111).^28^ Furthermore, platinum­(II) complexes of tetraphenylporphyrin derivatives were selected for investigation not only for their excellent stability but also because of the expectation that the porphyrin macrocycle is ‘locked’ in a planar configuration,^29,30^ although here we show that these compounds exhibit the frequently observed tendency of porphyrin macrocycles to adopt a saddle conformation in response to deposition on a metal substrate.^31^ The structures of the frontier molecular orbitals of the platinum porphyrins also indicate little or no contribution to their structures by the central metal cation (see )

The complex 5-(4-nitrophenyl)-10,15,20-triphenylporphyrin platinum­(II) (**1**) was deposited by sublimation at 533 K onto a clean Au(111) surface maintained at room temperature. After cooling to 4.3 K, the formation of self-assembled ribbon structures was observed (Figure 1a). The ribbon structures consist largely of double rows (Figure 1b), aligned with the fcc herringbone structure. Individual molecules appear to have 2-fold symmetry based on a saddle-shaped conformation of the metalloporphyrin macrocycle when adsorbed at a surface.^32,33^ Molecules in both rows have the same mutual orientation with an angle of 55° subtended between molecular normal axes passing through Pt atom and two opposing pyrrole rings (Figure 1c). Adjacent Pt core atoms are separated at distances of 1.50 nm (intrarow) and 1.76 nm (inter-row). The preference for double row formation is caused by hydrogen bonding between the nitro group and C–H groups of the porphyrin (Figure 1c).^34^ Since the single nitro group involved in hydrogen bonding is concealed at the interior of the double row island, there is no two-dimensional extension of the structure unless the molecular coverage reaches one monolayer (). Also, no intermolecular covalent bonding could be detected, and individual molecules could be separated from the molecular assembly by mechanical manipulation using the STM tip ().^35^ At Au(111) elbow sites, hydrogen bonding (H−) dimer and trimer structures of **1** were also observed (). Next, 5-(4-nitrophenyl)-10,15,20-tris­(3,5-di-t-butylphenyl)­porphyrin platinum­(II) (**2**) was deposited on clean Au(111) maintained at room temperature. Small molecular clusters were observed adsorbed at the elbow sites (Figure 1d). Magnified views of the topography show that the cluster is composed of three molecules in a windmill-like shape (Figure 1e). The Pt–Pt distance of 1.75 nm is similar to that found in the ribbon structure of **1**. A model of the chemical structure (Figure 1f) indicates that **2** H-trimers are also formed by hydrogen bonds between nitro groups and the porphyrin cores. H-Dimers of **2** were also occasionally observed (Figure S4c). Similar interactions albeit of differing geometry can also be found in the X-ray crystal structure of **2** (). It is likely that the bulky *tert*-butyl groups prevent the formation of the ribbon-like self-assembly found in **1** since van der Waals interactions between *tert*-butyl groups are relatively large favoring the H-trimer structure.

**Figure 1:**
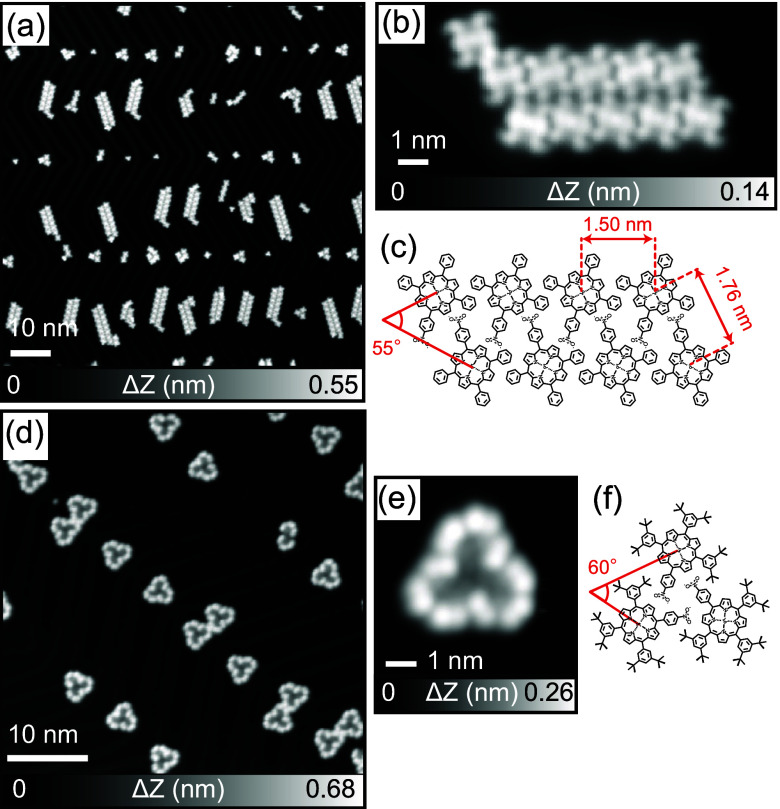
STM imaging and chemical structures. (**a**) STM topographies of **1** adsorbed on Au(111) and (**b**) a magnified view. (**c**) Proposed chemical structure of the self-assembled **1** ribbon structure. (**d**) STM topographies of **2** and (**e**) a magnified view. (**f**) Proposed chemical structure of the hydrogen bonding (H−) self-assembled **2** trimer. Measurement parameters: Sample bias voltage *V* = 0.1 V, tunneling current *I* = 10 pA in (a) and (d); *V* = – 900 mV and *I* = 50 pA in (b) and (e).

In both molecules, the d*I*/d*V* spectra of the monomer recorded in a bias range of – 2.0 to 2.0 V contain three distinct peaks at 1.7, – 1.0, and – 1.8 V, which correspond to the lowest unoccupied molecular orbital (LUMO), highest occupied molecular orbital (HOMO), and HOMO–1 levels, respectively (Figure 2a,b). Thus, the peripheral phenyl and di*tert*-butyl-phenyl groups do not affect the orbital energies of the porphyrin cores. The d*I*/d*V* map of **1** taken at the HOMO energy has two bright regions at the porphyrin core. This is similar to that of **2** but the two bright regions appear in closer proximity due to the larger dimensions of **2** (Figures 2c and 2d). LUMO orbitals are also distributed on the porphyrin core similarly for **1** and **2** (Figures 2c and 2d, right sides). The nitro groups appear as bright tails as indicated by white arrows, which are more obvious in the HOMO–1 orbitals shown in the left-hand panels of Figures 2c and 2d. The two bright spots and the tail establish the prochirality of the molecule on surface as defined by right (R) or left (L) angles relative to the nitro group axis (Figures 2e and 2f). Note that the d*I*/d*V* spectra and maps of **1** ribbon and **2** trimer are almost identical to those of the monomer (). We found that the ribbon is composed of **1** with both chiralities with molecules in the upper row exhibiting R chirality and those in the lower row exhibit L chirality (Figure 2g). In contrast, the trimer of **2** is composed of molecules with exclusively either R or L chirality (Figures 2h). Ribbon and trimer structures are most likely formed through intermolecular hydrogen bonding interactions involving nitro groups. In fact, C–H···O H-bonding is an important interaction in self-assembling and biochemical systems.^36,37^ For **1**, strong N–O···H–C H-bonding occurs between a nitro group of one molecule and an adjacent phenyl ring on the opposing row of molecules of opposite chirality (see also ). For **1** ribbons, strong hydrogen bonding involving nitro groups forces the self-assembled porphyrin units to be incommensurate with the gold substrate. The resulting different adsorption sites between R and l isomers results in variations in the observed contrast in the STM images, which is further enhanced by the intrinsic chirality of the structure. For **2**, There is a 3-way mutual interaction between nitro groups and the proton ortho to the nitro group in the adjacent molecules. Based on the dimensions, there is also the possibility of further stabilization of the trimer unit by further interaction with an adjacent t-butyl group (). Based on reported N–O···H–C H-bonding distances,^38,39^ inter-Pt distances for **1** of 1.8 Å (inter-row) and 1.3 Å (intrarow) are found, which support the model structure. For **2**, a Pt–Pt distance of 1.75 Å estimated from the model agrees well with that found experimentally.

**Figure 2:**
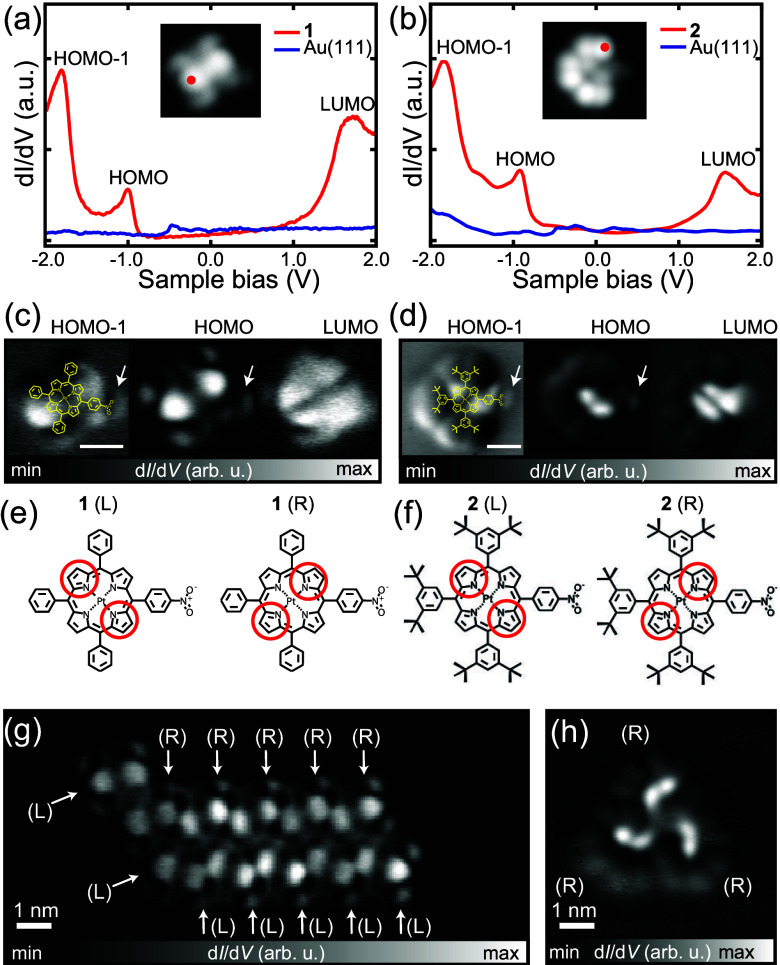
STS measurements of (**a**) **1** and (**b**) **2** monomers. d*I*/d*V* spectra were obtained at the site indicated by dots in the insets (red line) and on the pristine Au(111) surface for reference (blue line). (**c**) d*I*/d*V* maps recorded at the corresponding HOMO–1, HOMO, and LUMO level energies of **1** and (**d**) **2**. Scale bar shows 1 nm. (**e,f**) Chemical structures indicating chirality of **1** and **2** molecules on surface. (**g**) d*I*/d*V* maps recorded at the HOMO level energy of **1** ribbon and (**h**) **2** H-trimer (all-(R). For all-(S) see ). Measurement parameters: *I* = 50 pA and *V* = – 1.8, – 1.0 and 1.7 V for HOMO–1, HOMO and LUMO respectively in (c) and (d), *V* = – 1.0 V and *I* = 50 pA in (g) and (h).

Both the ribbon and H-trimer structures are eliminated by annealing at 160 °C for 10 min () followed by the emergence of small molecular clusters (dimers) absorbed mostly at the elbow sites on Au(111). In both cases, the whole dimers of **1** and **2** can be manipulated by using the STM tip () indicating that covalent bonds have been formed between porphyrin cores. Characteristic phenyl and 3,5-di*tert*-butyl-phenyl groups remain, indicating that the nitro groups have reacted in a reductive homocoupling leading to the synthesis of azobenzene moieties (Figures 3a,b) as has been observed in previous work.^28^ The d*I*/d*V* spectra of covalent bonding (C−) dimerized **1** and **2** are almost identical to those of the monomers and H-dimers obtained prior to annealing (Figures 3c,d). However, there are significant peak shifts to lower energy by approximately 0.2 eV, which is most probably related to differences in the vacuum level.^40^ Furthermore, calculated Bader charges () for the monomers and azobenzene dimers of **1** and **2** indicate that a large positive charge contribution located at the nitro N atoms of the monomer is eliminated by the reductive coupling process to the azobenzene dimer leading to differences in the charge transfer characteristics between the molecules and metal surface in that region (other parts of the molecules exhibit similar charge transfer prior to and following dimerization; see ). It should also be pointed out here that the reaction temperature of 160 °C is not sufficient to cause cyclodehydrogenation between phenyl substituents and the porphyrin core.^41^ Prior to annealing, the nitro group axis in the H-dimer is offset by 0.5 nm (Figures 3e,f). In contrast, azobenzene formation results in an almost symmetric linear dimer structure. The d*I*/d*V* maps of C-dimerized **1** and C-dimerized **2** indicate that both products are composed of one R and one L chiral units each and, interestingly, we found no azobenzene dimers with two of the same chiral units. Also, note that only the *trans* configuration of the azobenzene group is formed. For computed structures of the monomers and azobenzene dimers, see .

**Figure 3:**
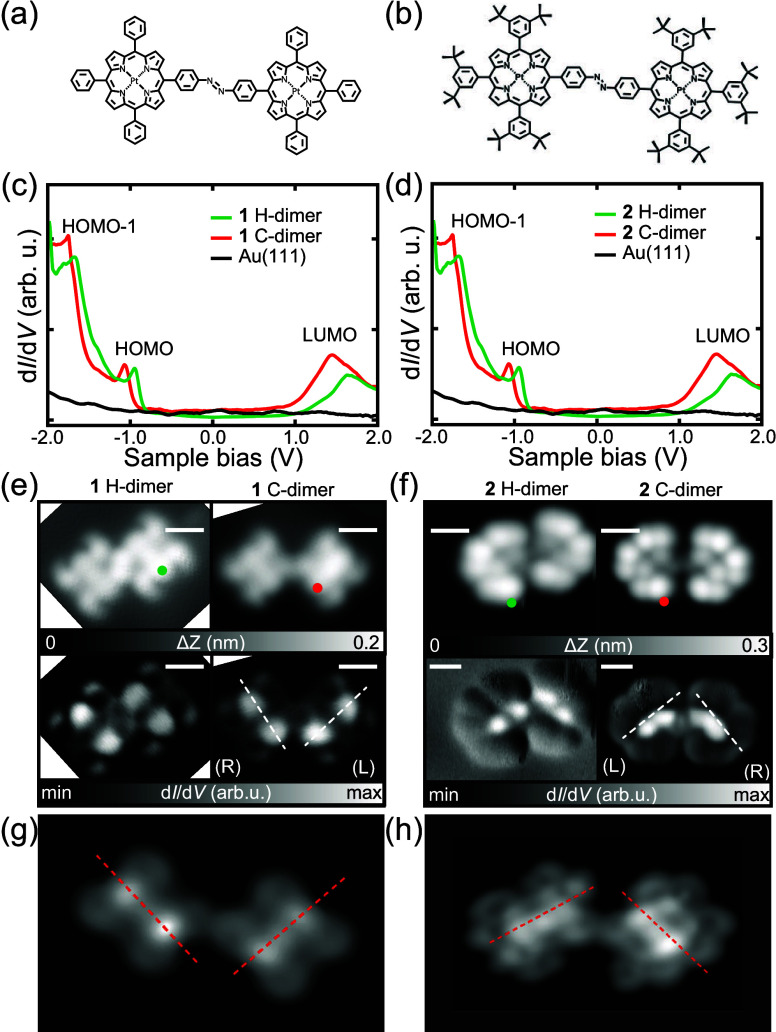
Chemical structures of (**a**) **1** covalent bonding (C−) dimer and (**b**) **2** C-dimer. (**c**,**d**) d*I*/d*V* spectra of Au surface (black line), dimer prior to annealing (green line), and representative d*I*/d*V* spectra of dimerized molecules following annealing (red line). Spectra were obtained at the green and red dots in (**e**,**f**) (for spectra measured at other locations see ). (**e**,**f**) STM topographies (upper panel) and d*I*/d*V* maps (lower panel) recorded at the HOMO energies of **1** (**e**) and **2** (**f**) before (left panel) and after annealing (right panel). (R) and (L) indicate the chirality of porphyrin rings. Simulated STM images of (**g**) **1** C-dimer and (**h**) **2** C-dimer. Measurement parameters: *V* = – 1.0 V and *I* = 50 pA in (**e**) and (**f**).

Since the coexisting RL chirality in C-dimerized **1** is the same as that in the ribbon structure, the self-assembled structure should promote C-dimerization based on nitro groups being in proximity in an appropriate geometry for reaction. In contrast, the self-assembled trimer of **2** composed of molecules of a single chirality as the majority of the self-assembled structure (Figure 1d), must be decomposed prior to reaction to a heterochiral C-dimer because its nitro groups are not in an appropriate proximity or geometry for azobenzene formation to occur. Thus, chiral **2** molecules diffuse on the surface until encountering a molecule in appropriate geometry (i.e., with complementary chirality) suitable for reaction to form a heterochiral C-dimer. Based on the different requirements for dimerization, the identity of the self-assembled structures should affect the yield of dimerization. It is notable in both computed structures of the dimerized compounds (Figure 3g, 3h, and ) that the azobenzene unit has its different moieties (−C_6_H_4_-, -N=N-, -C_6_H_4_-) all coplanar similar to the reported X-ray crystal structures of the parent azobenzene compound.^42^ The apparent necessity of this azobenzene geometry in the final dimerized structure enforces heterochirality in the final product since saddle conformations of each component porphyrin unit must be adopted to accommodate the coplanar azobenzene unit based on steric requirements (). This mechanism causes exclusivity of heterochirality for this dimerization reaction and might have connotations for applications of the related compounds in asymmetric synthesis. Furthermore, the azobenzene core adds the aspect of photoisomerization where trans-azobenzene might be converted to cis-azobenzene by applying an appropriate optical stimulus (ultraviolet light). In the present system, the large steric bulk of the porphyrin units prevents access to the cis isomer in the surface-constrained environment. However, smaller substituents of the azobenzene core might permit access to photoisomerization. This matter is currently under investigation in our laboratories.

To conduct a statistical analysis of the yields of different on-surface species, we used the Python program based on previous studies,^43^ where molecules are classified based on the molecular shape obtained from the STM topography. Further, we have developed a program to extract the outline of molecules with the open-source machine learning library, Scikit-learn in Python to implement the classification algorithm.^44,45^ Automated image processing was conducted to extract the outlines of the molecules. The measured images (particularly the first image collected directly following sample preparation) are tilted due to unintentional thermal drift and scanner creep mainly in the Z direction (Figure 4a). The blue and red lines in Figure 4b represent the cross sections of the STM topography in the X (fast scan) and Y (slow scan) directions, respectively. If there is no step in the image, the image can simply be fitted by

1 $$a\left(x-x_{0}\right)+b\left(y-y_{0}\right)+c\left(z-z_{0}\right)=0$$

where, (*a*, *b*, *c*) are the orthogonal vectors to the plane and (*x*_0_, *y*_0_, *z*_0_) are points that are on the plane.^46^ Consequently, the plane correction can be performed by subtracting the raw image from this calculated plane image. If steps are present in the image, an extended plane correction process is required. First, the image is divided into a 4 × 4 grid. Then, each of the 16 regions is fitted with , and subsequently the entire image is adjusted based on the best fit from each region (Figures 4c and 4d). This plane correction is sufficiently effective to correct images containing up to three steps. Since a gold atomic step is approximately 0.2 nm in height (Figure 4d), areas higher than 0.15 nm correspond to upper terraces and molecules adsorbed on the surface. Since the size of the molecules is a few nm, we eliminated these points only on the upper terrace. Subsequently, the height of the upper terrace was adjusted to that of the lower terrace by subtracting the step height from the upper terrace height (Figures 4e and 4f). Finally, molecular outlines were detected by applying the Depth-First Search algorithm^46^ to the binary image obtained from Figure 4e with a threshold of 0.1 nm. Each molecule was enclosed in a bounding box that fits its diagonal length, allowing counting of the molecules in the large-scale topography (Figure 4g). For shape classification, we used the Spectral Clustering method, with Zernike moment coefficients, contour length, and molecular height as feature parameters (Figure 4h).^47^ The molecules were grouped into five categories: Groups 1, 2, 3, 4, and 5 correspond to monomers, C-dimers, C-trimers, C-tetramers, and other large molecular clusters, respectively (Figure 4i). Using this approach, we automatically collected 928 and 1056 molecules from 10 and 11 images with scan sizes in the rage of 100 to 200 nm for reactions involving **1** and **2**, respectively. It should be noted that this approach can only be used to differentiate molecules/clusters with significantly different dimensions so that, for instance, unreacted (self-assembled) H-dimers cannot be distinguished from reacted azobenzene C-dimers. Also, large surface contaminants of sufficient size become classified as monomers using this method (see ). The overall accuracy of the method is 95.3%.

**Figure 4:**
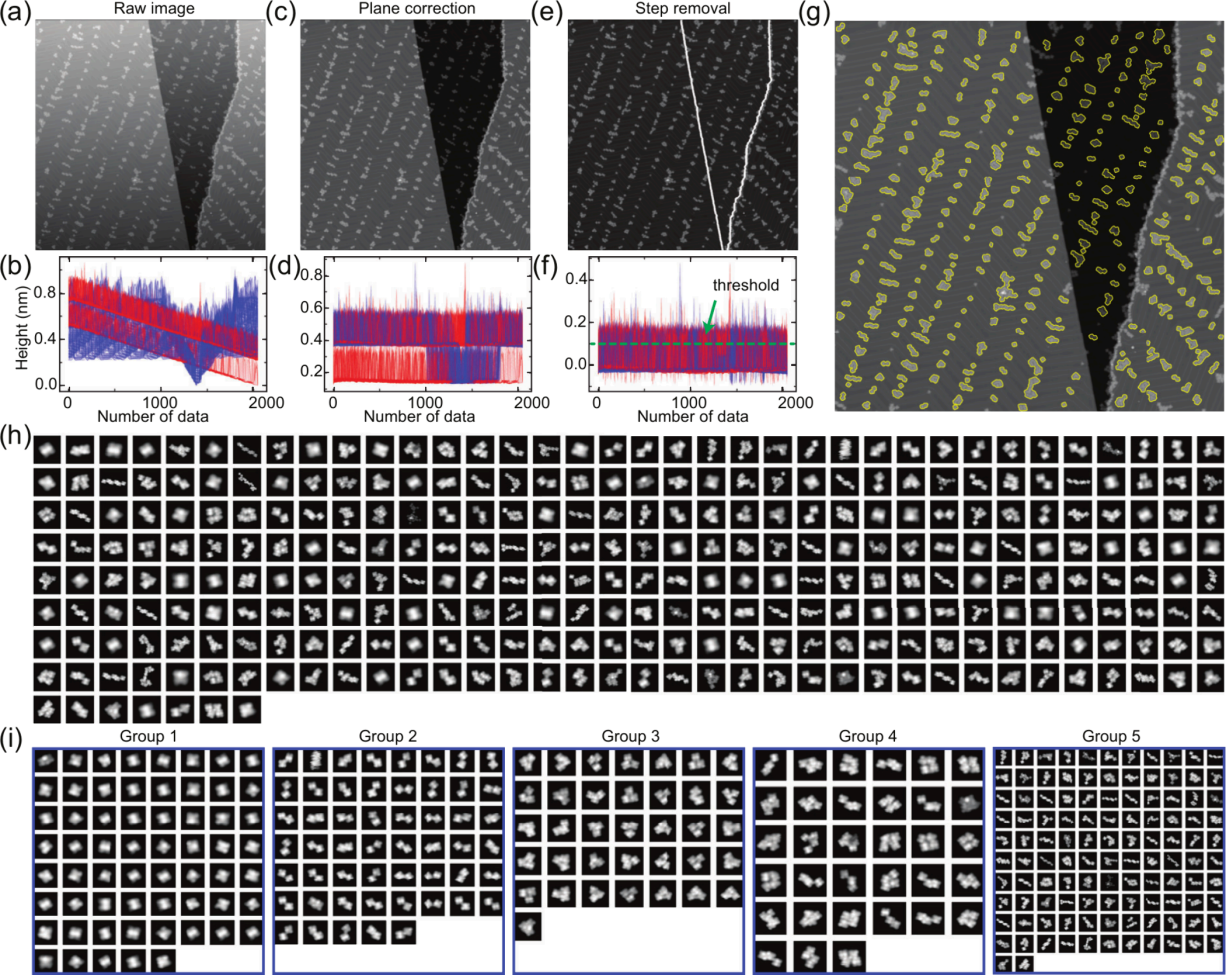
Automated STM image processing for molecule counting and morphology detection. (**a**) Raw STM topography including Z drift and creep and (**b**) its cross-section. Blue line is the cross-section in the X (fast scan) direction, red line is the cross-section in the Y (slow scan) direction. (**c**) Plane corrected image and (**d**) its cross-section. (**e**) Step-removed image and (**f**) its cross-section. (**g**) Extracted molecule contours superimposed on the plane corrected STM topography. (**h**) Automated enumeration of molecules. (**i**) Automated categorization of molecule shapes.

Figure 5a shows that the analysis classified the shapes of products into four and five groups for **1** and **2**, respectively, after annealing on Au(111) under identical reaction conditions. We found that the reaction yield of azobenzene from **1** (16%) is four times greater than that from **2** (4%). Thus, this analysis indicates that the homocoupling reaction of nitro groups was promoted by the self-assembled **1** ribbon structure, where molecules of one chirality are in close mutual contact with molecules of the other chirality (Figure 5b). In contrast, the homochiral self-assembled **2** trimer requires disassembly, subsequent monomer diffusion on the surface, then reaction with another molecule of opposing chirality in order to dimerize. These factors (multistep disassembly, requirement to react with opposing enantiomer) lead to a significant reduction in yield for monomer **2**, while the yield of monomer **1** might be assisted by proximity of opposing enantiomers in the ribbon structure. In on-surface syntheses, the chirality of the monomer is one of the most important factors since the chirality pattern tends to be maintained before and after the reaction.^48–50^ However, as shown here, if the product is composed of molecules of opposing chirality, the relationship of monomer units and chirality of preformed self-assembled structures can significantly affect the reaction yield. Although the assembly structures might be unstable at the reaction temperature (160 °C), any intermolecular interactions promoting the condensation to chiral products must occur during a relatively short period of time especially for **1** where disassembly is not required for formation of the chiral product. Regardless, this should still lead to preferential heterochiral reaction of **1** (compared to **2**) based on the close proximity of the reactants and their appropriate molecular geometries, and the necessity for **2** to disassemble prior to heterochiral azobenzene formation. Also, given that the vertical distances of the nitro groups from the surface and therefore their reactivities for azobenzene formation ought to be comparable (based on the similar distances from the surface: 2.44 Å (for **1**) and 2.47 Å (for **2**)), the large difference in reaction yield (4-fold for **1** over **2**) also indicates a substantial preference for the formation of heterochiral C-dimer for **1** supporting the role of the self-assembly structure in formation of the heterochiral C-dimer. Other factors such as diffusion and collision rates on the surface also probably affect reaction yield.

**Figure 5:**
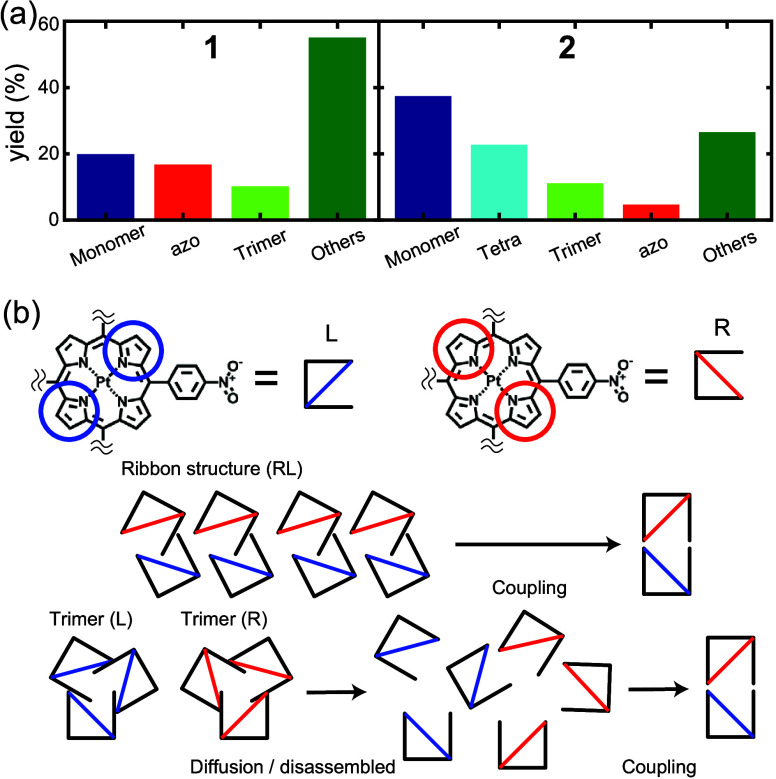
On-surface products of annealing **1** or **2**. (a) Yields of the different products obtained after annealing according to automated STM image processing. Total number of molecules is 928 for **1** (left panel) and 1056 for **2** (right panel). (b) Azo coupling processes involving **1** or **2**. Blue line denotes L isomer, red line denotes R isomer. Notably, homocoupling of **2** requires initial disassembly.

This study demonstrates that porphyrin chirality significantly influences the azo coupling process on surfaces. In **1**, the self-assembled ribbon structure promotes efficient coupling, resulting in higher yields of azobenzene-linked dimers with alternating chirality. The close molecular packing in these ribbons facilitates the reaction, preserving the original chirality. In contrast, **2** predominantly forms hydrogen bonding trimers, requiring dissociation and diffusion for covalent bonding dimer formation, leading to a lower yield. Our findings reveal that controlling molecular chirality and steric factors can optimize surface reactions, offering insights for designing surface-based molecular systems for nanomaterials and electronics.

## Methods

### STM Experiments

All experiments were performed using a custom built low-temperature ultrahigh vacuum scanning tunneling microscopy, operating under 1 × 10^–10^ mbar at 4.3 K. Clean Au (111) surfaces (MaTeck, purity: 99.999%) were obtained by repeated cycles of Ar^+^ sputtering and subsequent annealing at 450 °C. The molecules were sublimated from a quartz crucible (Kentax GmbH) at 300 °C while the Au (111) substrate was kept at room temperature. STM topographies were obtained in constant current mode. The differential conductance was measured with the digital lock-in amplifier with a modulating voltage of *V*_AC_ = 10 mV at a frequency of 512 Hz.

### Synthesis

5-(4-Nitrophenyl)-10,15,20-triphenylporphyrin and 5-(4-nitrophenyl)-10,15,20-tris­(3,5-di-*t*-butylphenyl)­porphyrin were synthesized according to literature methods.^51,52^

#### 5-(4-Nitrophenyl)-10,15,20-triphenylporphyrin platinum­(II) (**1**)

Platinum­(II) was inserted into 5-(4-nitrophenyl)-10,15,20-triphenylporphyrin (200 mg, 0.3 mmol) exactly according to Turner et al.^53^ Yield: 160 mg (62%). ^1^H NMR (400 MHz, CDCl_3_) δ = 8.75 (d, *J* = 5.3 Hz, 2H), 8.72 (m, 4H), 8.66 (d, *J* = 5.3 Hz, 2H), 8.58 (d, *J* = 8.8 Hz, 2H), 8.34 (d, *J* = 8.8 Hz, 2H), 8.11 (m, 6H), 7.73 (m, 9H) ppm. ^13^C NMR (101 MHz, CDCl_3_) δ = 148.1, 141.3, 140.9, 140.0, 134.6, 133.7, 131.0, 130.8, 130.6, 129.8, 127.9, 126.9, 122.9, 122.7, 121.9, 119.7 ppm. MALDI-TOF-MS (dithranol; + ve) *m*/*z*: found 853.3, calc. for C_44_H_28_N_5_O_2_Pt 853.2 ([M + H]^+^).

#### 5-(4-Nitrophenyl)-10,15,20-tris­(3,5-di-t-butylphenyl)­porphyrin platinum­(II) (**2**)

5-(4-Nitrophenyl)-10,15,20-tris­(3,5-di-*t*-butylphenyl)­porphyrin (0.5 g, 0.5 mmol) was treated with PtCl_2_ (1.5 equiv, 200 mg) in refluxing benzonitrile (50 mL) for 18 h under a dry nitrogen atmosphere. The reaction was performed in two stages: (1) heating for 3h at 110 °C then (2) refluxing for 18 h. After reaction completion, benzonitrile was removed under reduced pressure. The resulting orange solid was subjected to column chromatography (SiO_2_; dichloromethane/hexane 60:40 v/v). Product containing fractions were combined and the solvents were removed under reduced pressure. The resulting bright orange solid was recrystallized from dichloromethane/methanol yielding **2** as dark orange microcrystalline powder. Yield: 405 mg (68%). ^1^H NMR (400 MHz, CDCl_3_) δ = 8.85 (d, *J* = 5.3 Hz, 2H), 8.84 (s, 4H), 8.65 (d, *J* = 5.3 Hz, 2H), 8.63 (d, *J* = 8.8 Hz, 2H), 8.37 (d, *J* = 8.8 Hz, 2H), 8.03 (d, *J* = 1.8 Hz, 4H), 8.02 (d, *J* = 1.8 Hz, 2H), 7.80 (t, *J* = 1.8 Hz, 16H), 1.52 (s, 54H) ppm. ^13^C NMR (101 MHz, CDCl_3_) δ = 149.0, 148.8, 147.8, 141.5, 141.3, 141.1, 140.4, 139.6, 134.6, 131.7, 131.3, 131.1, 129.5, 129.1, 129.0, 124.4, 124.1, 122.0, 121.3, 118.8, 35.1, 31.8 ppm. FTIR (ATR): ν = 2957 (s, C–H (Ar)), 2903 (m, C–H (CH_3_)), 2867 (m, C–H (CH_3_)), 1593 (s, C–C (Ar)), 1523 (s, N–O (NO_2_)), 1342 (s, N–O (NO_2_)), 827 (s, C–H (Ar, *p*-disubstituted)) cm^–1^: HRMS (ESI+) *m*/*z*: found 1189.5647, calc. for C_68_H_76_N_5_O_2_Pt 1189.5641 ([M + H]^+^).

### X-ray Crystallography

Crystals suitable for X-ray diffraction were grown by slow diffusion of hexane into a solution of **2** in dichloromethane. Data collection was performed using MoKα radiation (λ = 0.71073 Å) on a RIGAKU VariMax Saturn diffractometer equipped with a charge-coupled device (CCD) detector or a Bruker APEX CCD diffractometer. Prior to the diffraction experiment the crystals were flash-cooled to the given temperature in a stream of cold nitrogen gas. Cell refinements and data reductions were carried out using the d*trek program package in the CrystalClear software suite.^54^ The structures were solved using a dual-space algorithm method (SHELXT)^55^ and refined by full-matrix least-squares on F2 using SHELXL-2014 ^56^ in the WinGX program package.^57^ Non-hydrogen atoms were anisotropically refined and hydrogen atoms were placed on calculated positions with temperature factors fixed at 1.2 times Ueq of the parent atoms and 1.5 times Ueq for methyl groups. Crystallographic data (excluding structure factors) have been deposited with the Cambridge Crystallographic Data Centre with CCDC reference numbers 2350301 (**2**). Copies of the data can be obtained, free of charge, on application to CCDC, 12 Union Road, Cambridge CB2 1EZ, UK http://www.ccdc.cam.ac.uk, e-mail: data_request@ccdc.cam.ac.uk, or fax: + 44 1223 336033.

### Computational Methods

The simulations were performed using the Siesta code.^58,59^ As exchange-correlation functional, we have used the GGA-PBE functional.^60^ The DFT interactions were supplemented with a set of Grimme force-fields, to simulate the dispersion interactions (van der Waals).^61^ The systems were confined to unit cells that allow the study of periodically Au(111) surface and included 3 atomic layers for all the investigated systems. The supercell sizes vary from 10 × 10 Au atoms for monomer s adsorption to 9 × 15 Au atoms for C-dimers. The length of the cells along the OZ axis was 40 Å for all systems, thus allowing a vacuum level of more than 25 Å that we consider large enough to avoid the artificial influence of the electric charge from one cell to another. The Monkhorst–Pack grid for the integrals in the Brillouin zone was 1 × 1 × 1. As relaxation methods, we used the Conjugated Gradient for all the systems. As basis sets, we used double-ζ polarized (DZP) with an energy shift of 100 meV for all atoms. The systems were allowed to find the local minima until the maximum gradient in the relaxed structure was below 0.04 eV/A. The simulations of STM images were performed by using the Tersoff-Hamman approximation. That method considers that the STM image is determined by the local density of states (LDOS).^62^ We computed LDOS in the energy window spanning from the Fermi level of the system up to 1 eV below the Fermi level, corresponding to an external bias of 1 V. The surface was scanned by searching for a given constant value of the LDOS (essentially a ″constant current″ STM experiment).

## Supplementary Material









## Notes

Calculated frontier orbital structures including levels (Table S1), additional STM imaging and molecular manipulation data, X-ray crystal structure data for **2**, additional d*I*/d*V* spectra, detailed model structures of ribbons and trimers, molecule–surface charge transfer profiles, computed structures of **1** and **2** monomers and azobenzene dimers on Au(111) surface, Bader charges for monomers and azobenzene dimers (Table S2), chemical analytical data for compounds **1** & **2** (PDF)
Coordinates of the calculated structures (PDF)
Crystallographic information (CIF)
checkCIF/PLATON report (PDF)
