# Supplementary material for: On-Surface Synthesis of Azobenzene-Linked Porphyrin Derivatives
Source: J Phys Chem Lett. 2025 Nov 6;16(45):11833–41. doi: 10.1021/acs.jpclett.5c03174 (PMC12621240; doi:10.1021/acs.jpclett.5c03174)
Supplement: Supplementary file 1 [file jz5c03174_si_001.pdf]

Supporting Information

On-surface synthesis of azobenzene-linked porphyrin derivatives

Yuji Isshiki[1], Donglin Li[1], Saranyan Vijayaraghavan[2], Kewei Sun[1], Huynh Thien Ngo[3], Luiza Buimaga-Iarinca[4]*, Yoshitaka Matsushita[5], Edward A. Neal[3], Cristian Morari[4], Jonathan P. Hill[3]*, Shigeki Kawai[1,6]*

[1]Center for Basic Research on Materials, National Institute for Materials Science, 1-2-1 Sengen, Tsukuba, Ibaraki 305-0047, Japan.

[2]CSIR-Central Electrochemical Research Institute, Karaikudi 630003, India.

[3]Research Center for Materials Nanoarchitectonics, National Institute for Materials Science, 1–1 Namiki, Tsukuba, Ibaraki 305-0044, Japan.

[4]National Institute for Research and Development of Isotopic and Molecular Technologies (NIRDIMT), 65-103 Donath, Ro-400293, Cluj-Napoca, Romania.

[5]Research Network and Facility Services Division, National Institute for Materials Science, 1-2-1 Sengen, Tsukuba, Ibaraki 305-0047, Japan.

[6]Graduate School of Pure and Applied Sciences, University of Tsukuba, Tsukuba, Ibaraki, 305-0047, Japan.

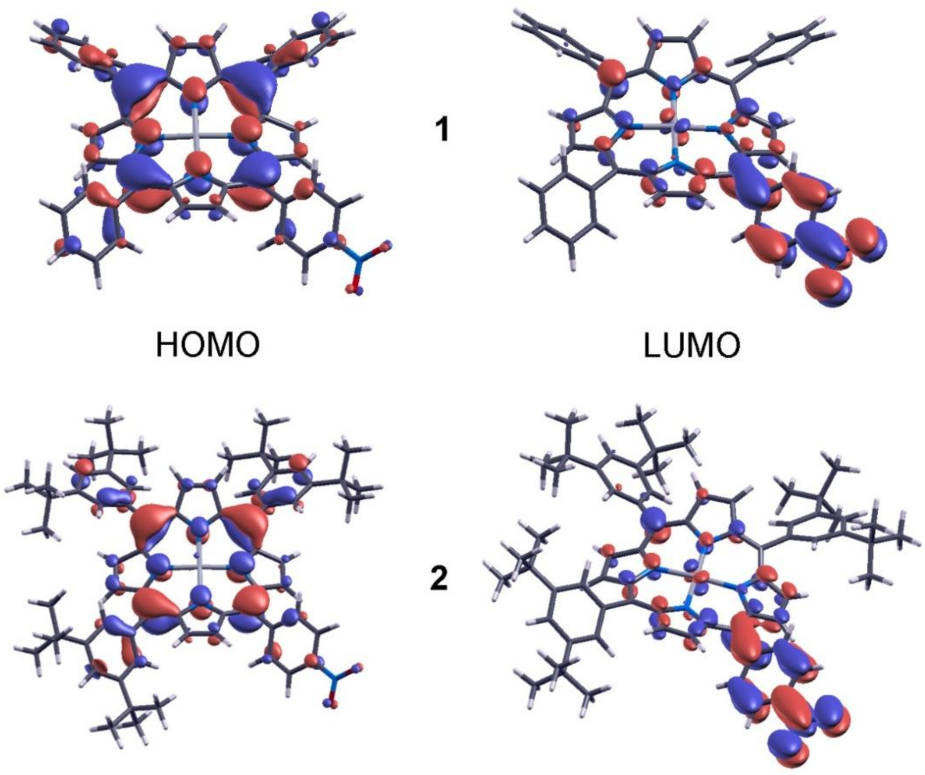

Figure S1. Frontier molecular orbitals of **1** and **2** reveal only minor contributions by the central Pt^{2+} cation to the structures of the HOMOs and LUMOs of the molecules.

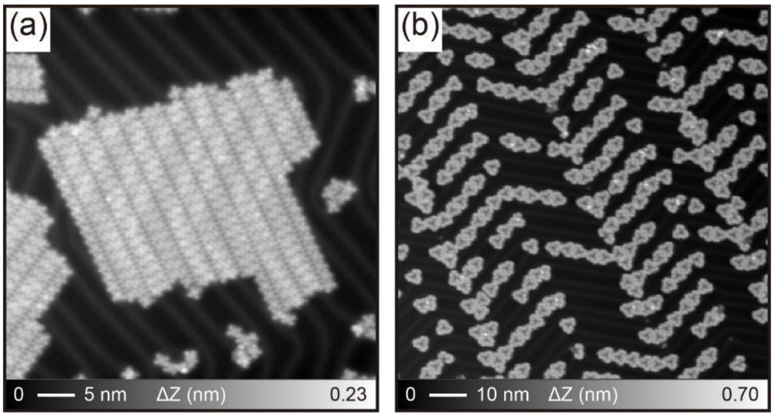

Figure S2. Increased coverage STM images for 1 and 2. (a) Tightly-packed monolayer domain of compound **1** on Au(111) surface. (b) At higher coverage, self-assembled trimers of **2** form poorly ordered chains.

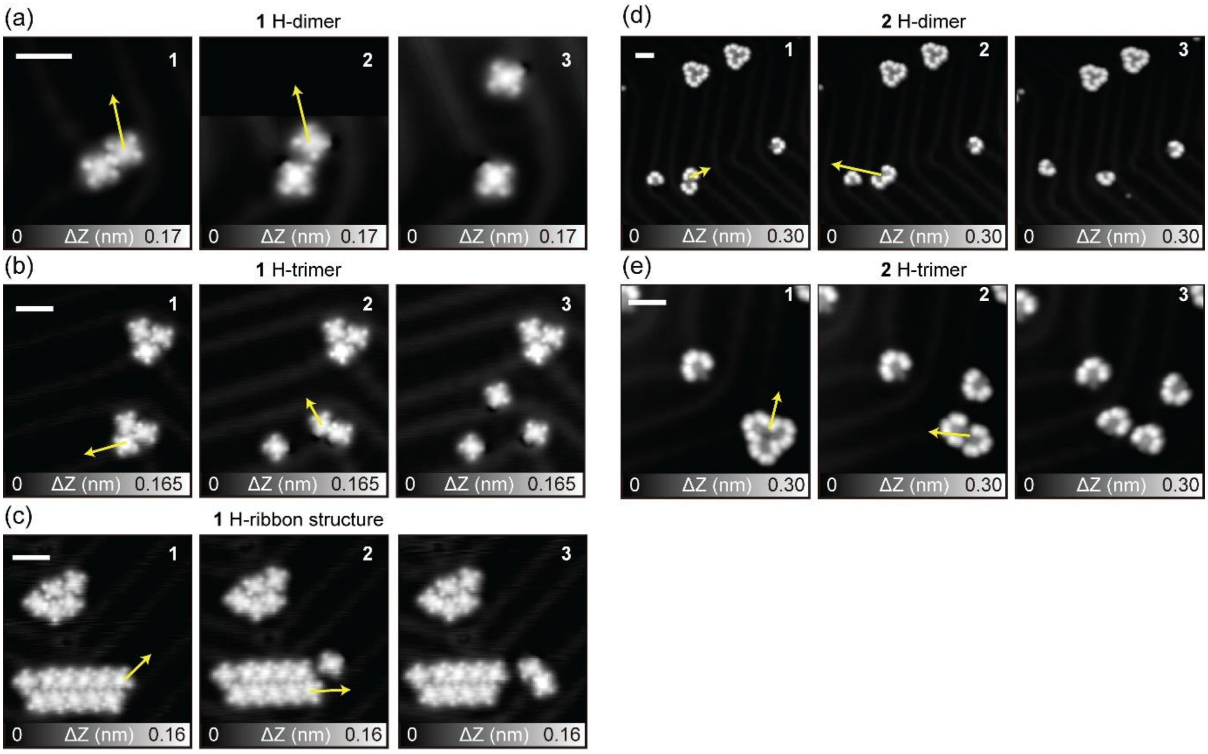

Figure S3. **Atomic manipulation for dimer, trimer and ribbon structures.** Atomic manipulation of (a) hydrogen bonding (H-)dimer, (b) H-trimer, (c) H-ribbon structure of **1**, and (d) H-dimer and (e) H-trimer of **2**. All scale bar is 3 nm. To investigate the bonding of the self-assembled structures of **1** and **2**, we separated the single molecules from the assemblies using an atomic manipulation technique. The tip was gradually brought closer to the target molecule with a bias of 1 mV and moved in the direction of the yellow arrow. By repeating this process, the single molecules were moved to the target position. When the atomic manipulation method was applied to the H-dimer, H-trimer, and H-ribbon structure of **1**, the single molecule could be pulled out in two to three processes. In the sample before heating, the molecules were assembled through hydrogen bonding between the nitro group and the polyphyrin, which could be decomposed by atomic manipulation.

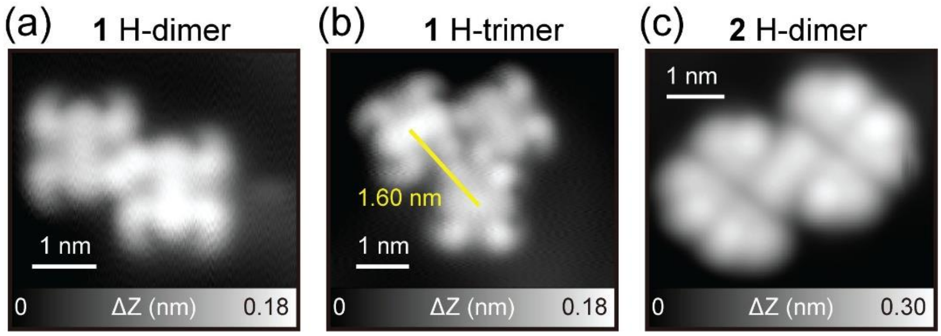

Figure S4. **STM image and d*I*/d*V* spectrum in self-assembly structures.** STM images of self-assemble structures (**a**) **1** H-dimer and (**b**) **1** H-trimer, and (c) **2** H-dimer. In addition to the d*I*/d*V* spectrum of the main monomer, the electronic structure of the self-assembled structure was investigated. As with the monomer, energy peaks were observed for the HOMO-1, HOMO, and LUMO levels in the bias range from -2.0 to 2.0 V. The peak values were close to those of the monomer, and the energy difference between the HOMO and LUMO was 2.6 to 2.8 eV for all cases. Although the molecules formed assemblies through hydrogen bonds, the electronic structure did not change significantly.

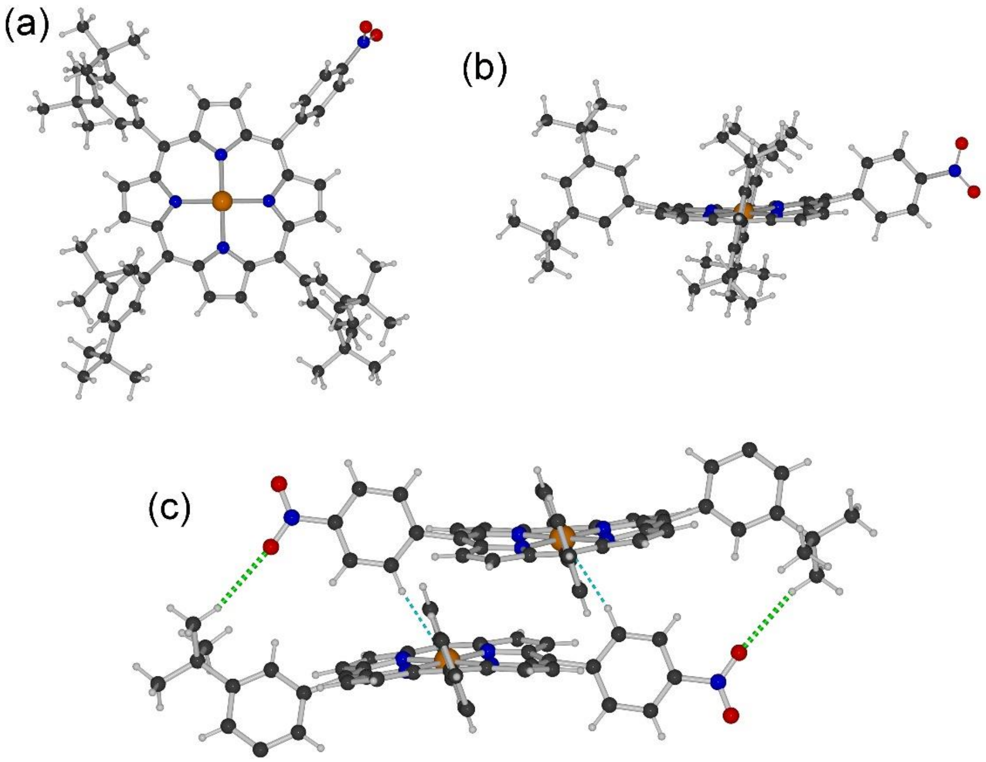

Figure S5. X-ray crystal structure of compound **2**,

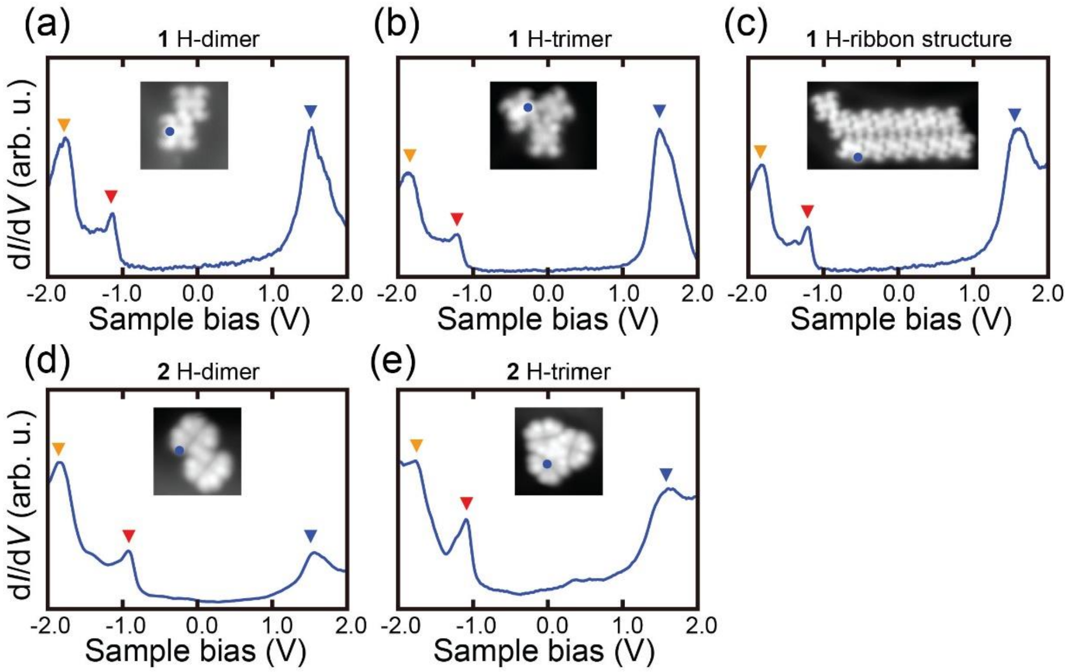

Figure S6. **d*I*/d*V* spectrum of self-assembled structures**. (**a**) **1** H-dimer, (**b**) **1** H-trimer, (**c**) **1** H-ribbon structure, (**d**) **2** H-dimer, and (**e**) **2** H-trimer. Blue dots on inset are the point where d*I*/d*V* spectrum was obtained. Orange, red and blue triangles indicate HOMO-1, HOMO and LUMO levels, respectively.

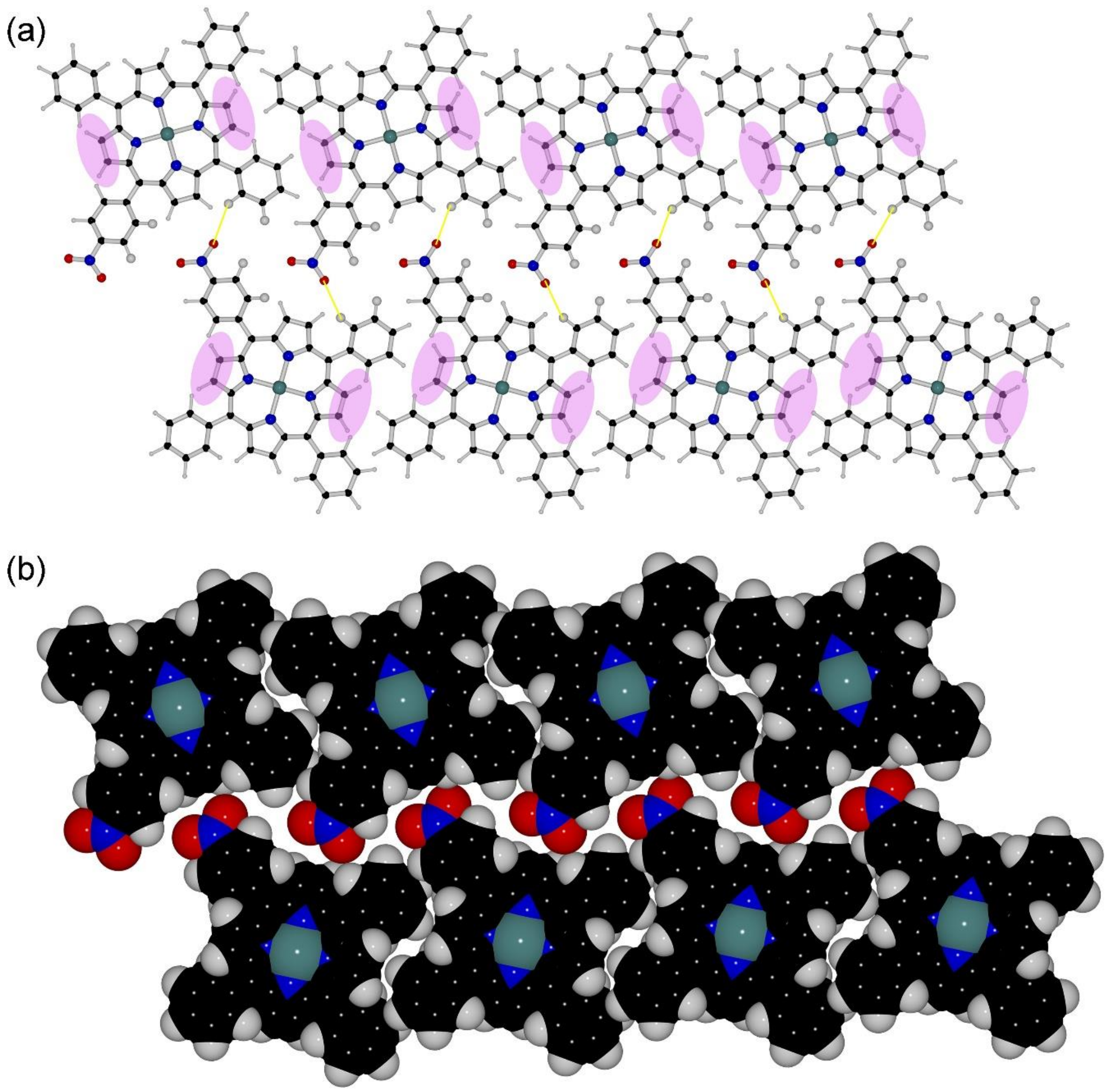

Figure S7. Model structure of **1** self-assembled ribbon. (a) NO_2…H-C hydrogen bonding (indicated by yellow lines) stabilizes the ribbon structure. Based on proximity, hydrogen bonding is likely to occur between the nitro group oxygen atoms and phenyl protons (shown enlarged in the image). Pink shading indicates high contrast areas due to tetrapyrrole saddle structure. (b) Space-filling representation of the structure shown in (a) revealing close-contacts of nitro O atoms and phenyl C-H.

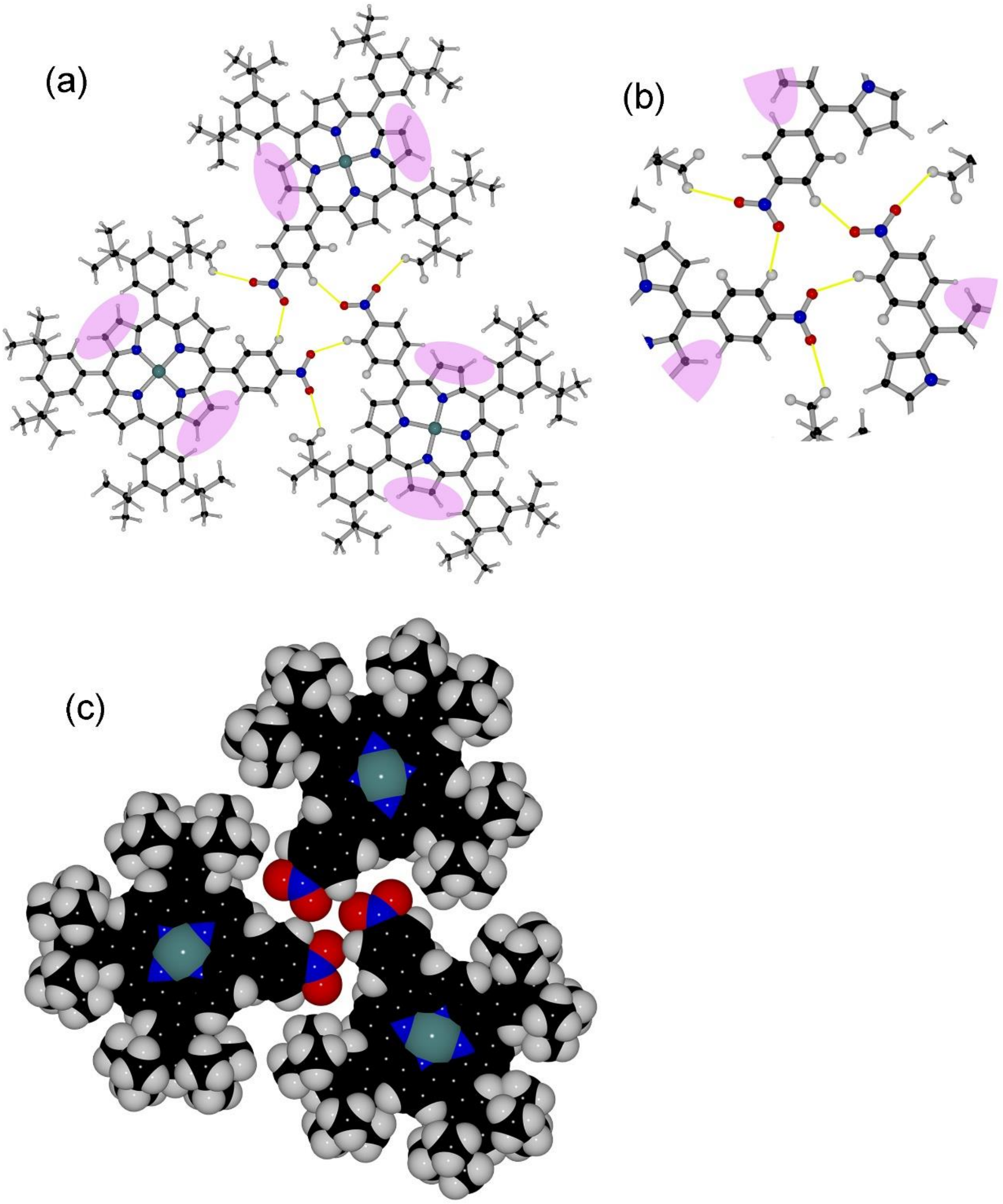

Figure S8. Model structure of **2** self-assembled trimer. (a) Mutual NO_2…H-C hydrogen bonding (indicated by yellow lines) is responsible for the trimer structure. Based on proximity, hydrogen bonding is also likely to occur between the nitro group oxygen atoms and t-butyl protons of an adjacent molecule (also shown enlarged in the image). Pink shading indicates high contrast areas due to tetrapyrrole saddle structure. (b) Detail of the hydrogen bonding region of the **2** trimer. (c) Space-filling representation of the structure shown in (a) revealing close-contacts of nitro O atoms and phenyl C-H.

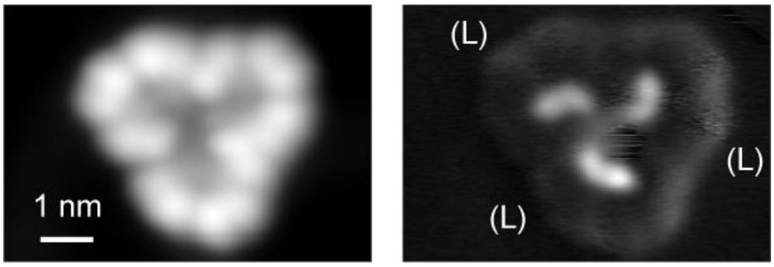

Figure S9. d*I*/d*V* maps recorded at the HOMO level energy of **2** H-trimer for the complementary all-(S) trimer. V = –1.1 V, I = 50 pA.

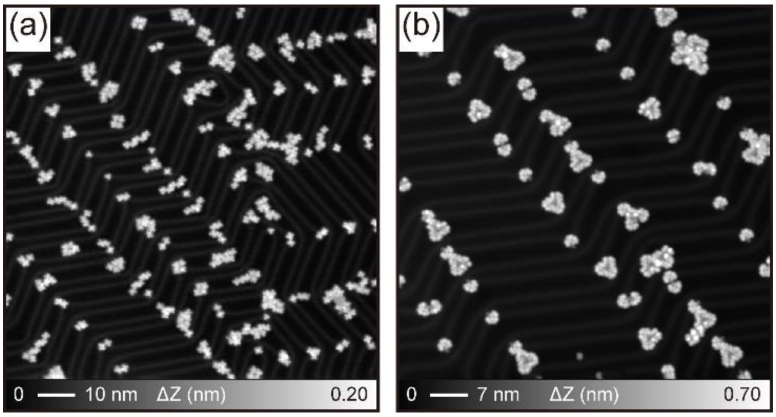

Figure S10. **Large scale images after annealing sample.** STM images of (a) **1** and (b) **2** on Au(111) after annealing the sample at 433 K for 10 min. Measurement parameters: $U = 0.1$ *V*, $I = 10$ pA in (a,b).

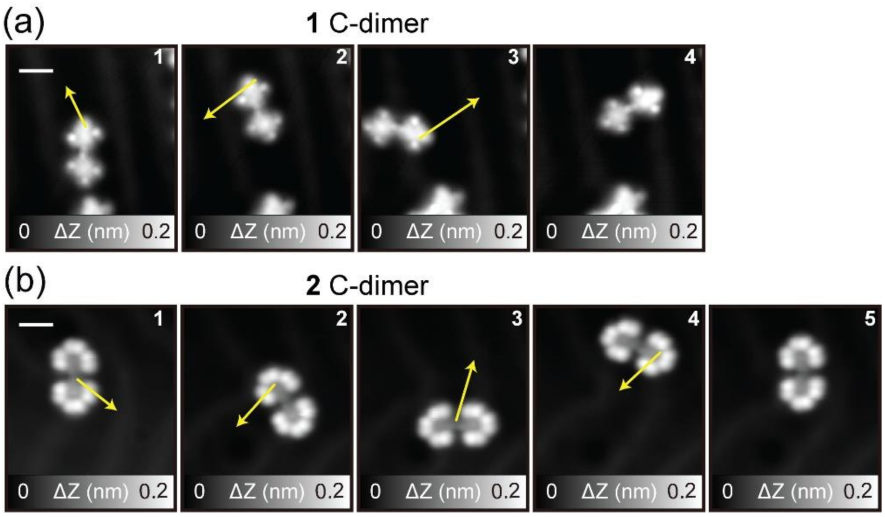

Figure S11. **Atomic manipulation for dimerized structures.** Atomic manipulation of (**a**) **1** covalent bonding (C-)dimer and (**b**) **2** C-dimer. Scale bar is 2 nm. After the azo coupling reaction, the atomic manipulation process as seen in Fig. S3 was applied to the dimer molecule. Because the azobenzene was formed, the two porphyrins moved together. Even after multiple manipulation processes, the two porphyrins were not broken down, indicating that they were strongly bound to each other by the azo bond.

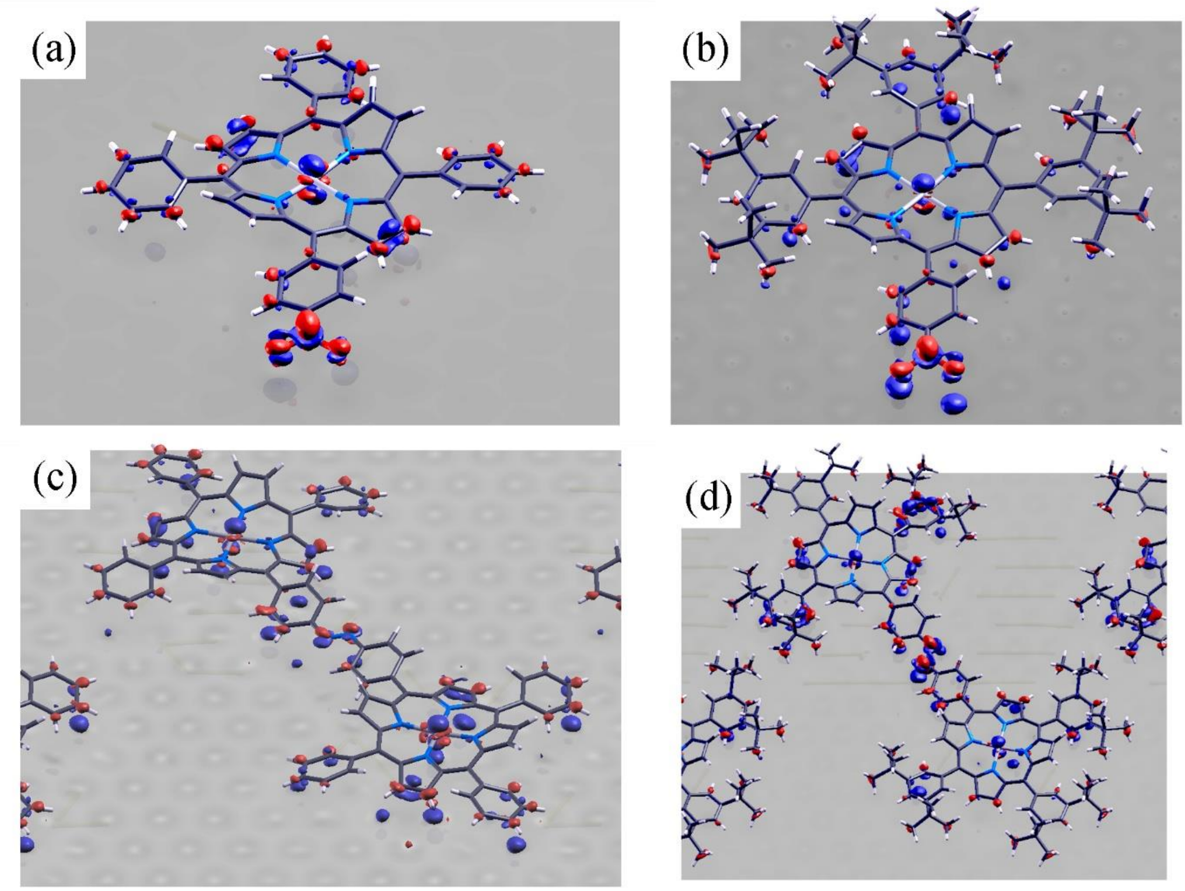

Figure S12. Charge transfer between molecule and surface for monomers and dimers. (a) **1** monomer, (b) **2** monomer. (c) **1** azobenzene C-dimer. (d) **2** azobenzene C-dimer. Contour plots are represented at 0.003 e/B^3. A transparent plane was inserted between the molecule and surface for clarity, to focus on the molecule's contour plot. Red - negative charge, blue – positive charge.

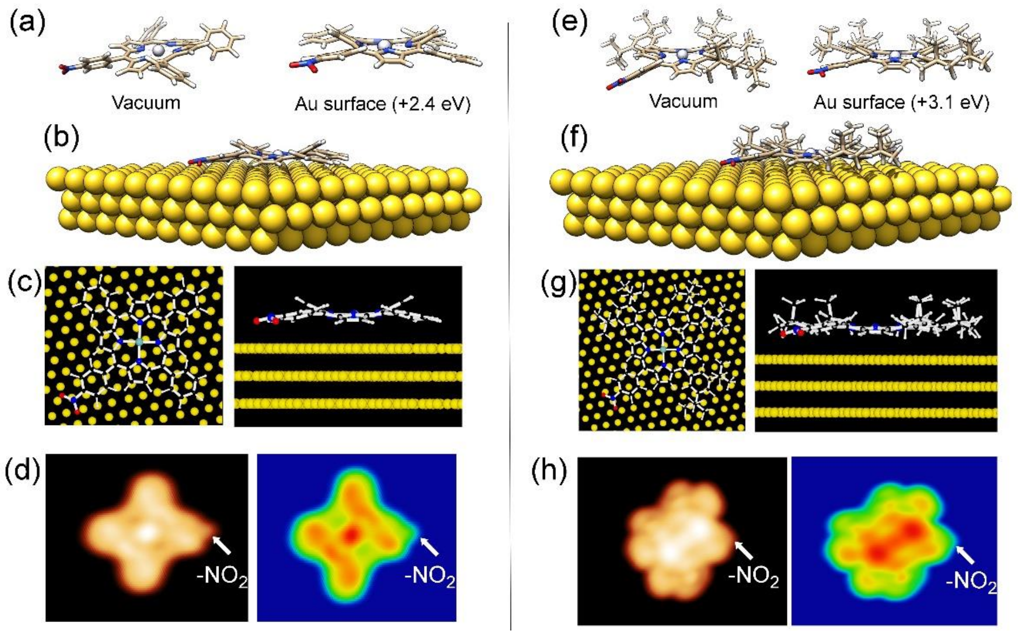

Figure S13. **Computed structures of 1 and 2.** (a) Compound **1**: (left) conformation in vacuum; (right) conformation when adsorbed on Au surface. Conformation in vacuum is more stable by 2.4 eV. (b) **1** on Au(111) surface. (c) Plan and edge views of **1** on Au(111). Note the saddle structure with opposing pyrrole groups respectively pointing towards and deflected away from the surface. (d) Simulated STM images of **1** on Au(111). Position of nitro group is indicated. (e) Compound **2**: (left) conformation in vacuum; (right) conformation when adsorbed on Au surface. Conformation in vacuum is more stable by 3.1 eV. (f) **2** on Au(111) surface. Note that 12 out of 18 methyl groups face the surface with 6 deflected away from the surface. (g) Plan and edge views of **2** on Au(111). Saddle structure is obscured by t-butyl groups. (h) Simulated STM images of **2** on Au(111). Position of nitro group is indicated.

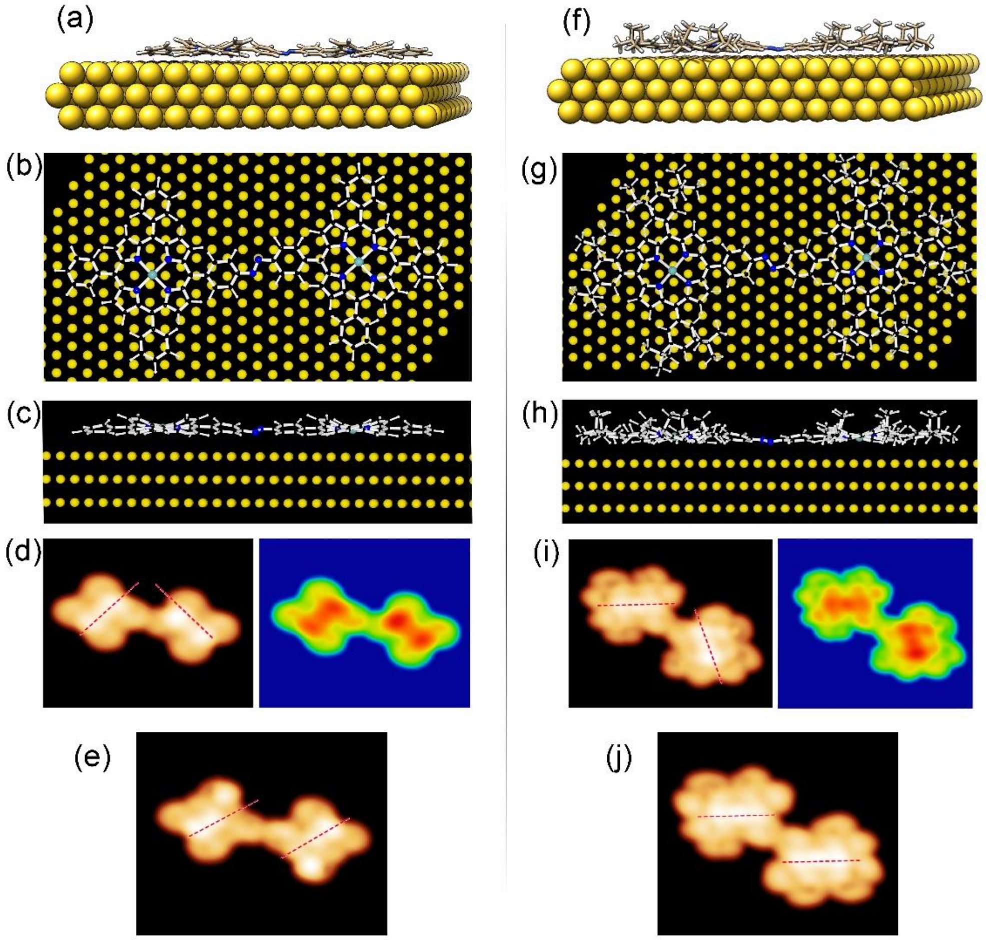

Figure S14. **Computed structures of azobenzene 1 C-dimer and azobenzene 2 C-dimer.** (a) Azobenzene **1** dimer on Au(111). (b) Plan and (c) edge views. (d) Simulated STM image of the **1** C-dimer. Red dashed lines highlight the perpendicular orientation of the macrocyclic saddles (i.e., heterochirality). (e) Simulated STM image of the homochiral azobenzene **1** C-dimer (i.e., not experimentally observed) with a parallel orientation of macrocyclic saddling (indicated by red dashed lines). (f) Azobenzene **2** C-dimer on Au(111). (g) Plan and (h) edge views. (i) Simulated STM image of the **2** C-dimer. Red dashed lines highlight the roughly perpendicular orientation of the macrocyclic saddles (i.e., heterochirality). (j) Simulated STM image of the homochiral azobenzene **2** C-dimer (i.e., not experimentally observed) with a parallel orientation of macrocyclic saddling indicated by red dashed lines.

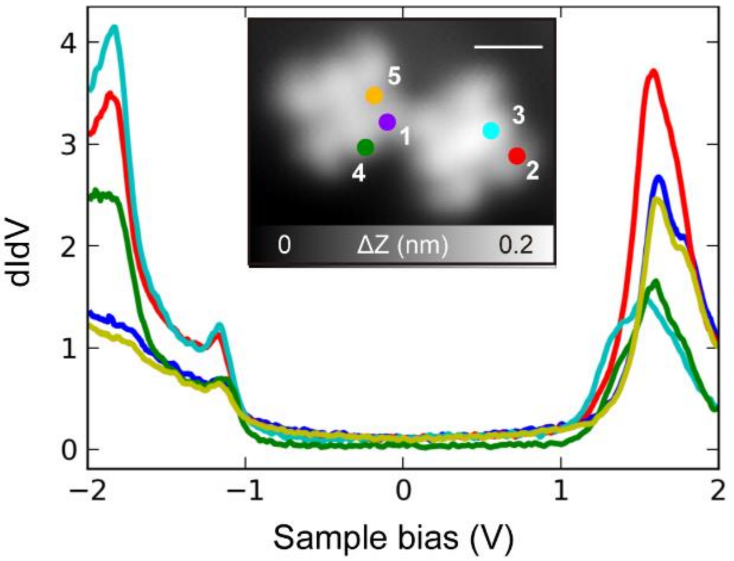

Figure S15. d*I*/d*V* spectra of **1** C-dimer (after annealing) taken at the indicated points on the molecule.

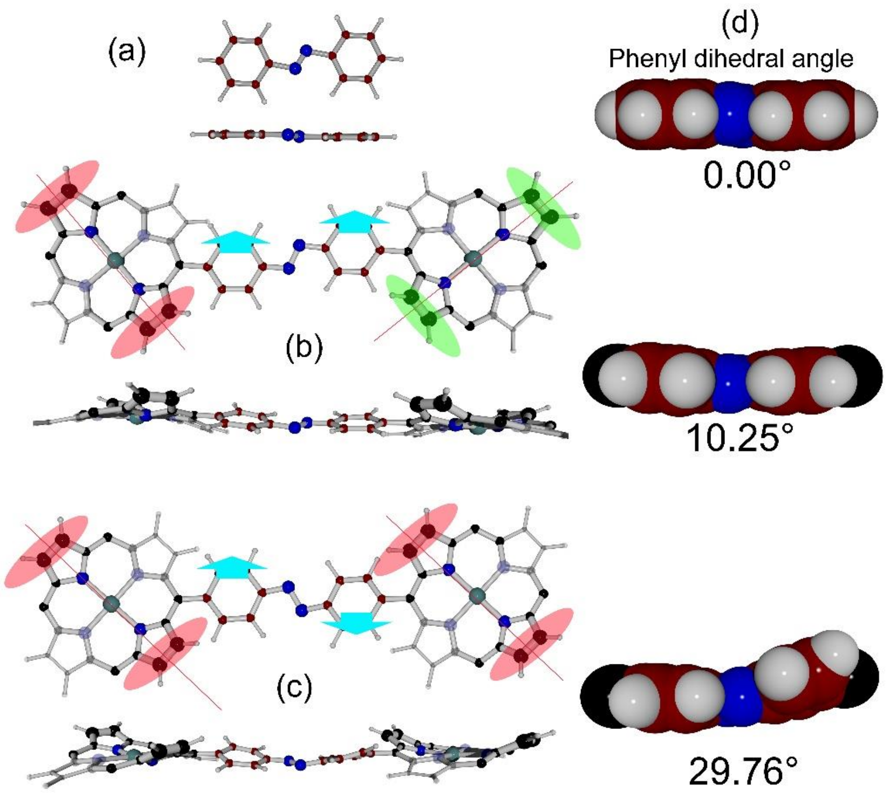

Figure S16. **Driving force for heterochirality in azobenzene C-dimers of 1 and 2**. (a) X-ray crystal structure of azobenzene (see Brown, C. J. *Acta Cryst.* **21**, 146 (1996)) indicates the preference for a highly planar structure with a dihedral angle subtended between phenyl groups of 0.00°. (b) Calculated structure of azobenzene **2** C-dimer (consistent with STM observations) contains a planar azobenzene unit (phenyl dihedral angle = 10.25°). Planar azobenzene forces relative mirror geometry of macrocycle saddling. Blue arrows indicate azobenzene phenyl C-H remote from surface (deflects proximal pyrrole unit towards surface). Red ellipses indicate pyrrole groups deflected away from surface; green ellipses: macrocylic saddling with opposing chirality to red ellipses. (c) Calculated structure of azobenzene **2** C-dimer having parallel (i.e., homochiral) orientation of macrocyclic saddles (not observed) contains a non-planar azobenzene unit (phenyl dihedral angle = 29.76°). Red ellipses indicate pyrrole groups deflected away from surface. (d) Space-filling representations and phenyl dihedral angles of the different azobenzene units. Based on these data, it is proposed that the preference for coplanarity of the azobenzene unit is the driving force for heterochirality in azobenzene C-dimers of **1** and **2**.

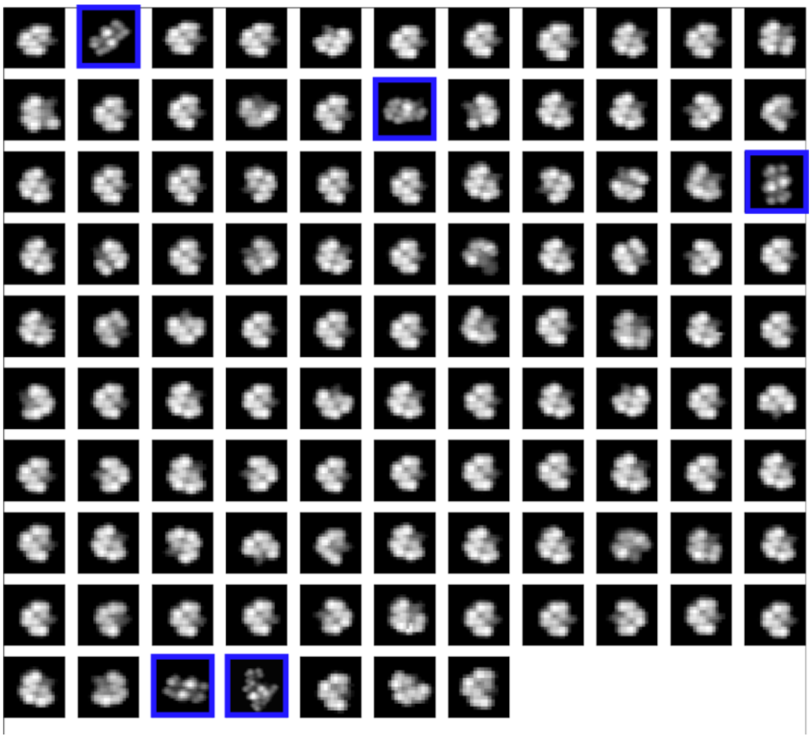

Figure S17. Automatic image collection of monomers (**2**). Although overall accuracy of the method is 95.3%, large surface contaminants of sufficient size can be accidentally classified as monomers (see items in blue squares).

Table. S1. Energy levels of LUMO, HOMO and HOMO-1 in self-assembled structures.

	**1**			**2**	
	**Dimer**	**Trimer**	**Ribbon**	**Dimer**	**Trimer**
**LUMO [eV]**	1.53	1.49	1.62	1.56	1.60
**HOMO [eV]**	-1.13	-1.21	-1.21	-1.00	-1.10
**HOMO-1 [eV]**	-1.76	-1.86	-1.83	-1.86	-1.77

Table 2. Bader charges for nitro and azobenzene heteroatoms monomers and azobenzene dimers of **1** and **2**.

State	Atom	Bader charge (surface)	Bader charge (vacuum)
**1** (monomer; nitro)	N	+0.43	+0.51
	O1	-0.52	-0.45
	O2	-0.51	-0.45
**2** (monomer; nitro)	N	+0.59	+0.63
	O1	-0.43	-0.38
	O2	-0.48	-0.41
**1** (dimer; azobenzene)	N1	-0.51	-0.59
	N2	-0.63	-0.60
**2** (dimer; azobenzene)	N1	-0.40	-0.36
	N2	-0.47	-0.36

Chemical analytical data for 1 & 2

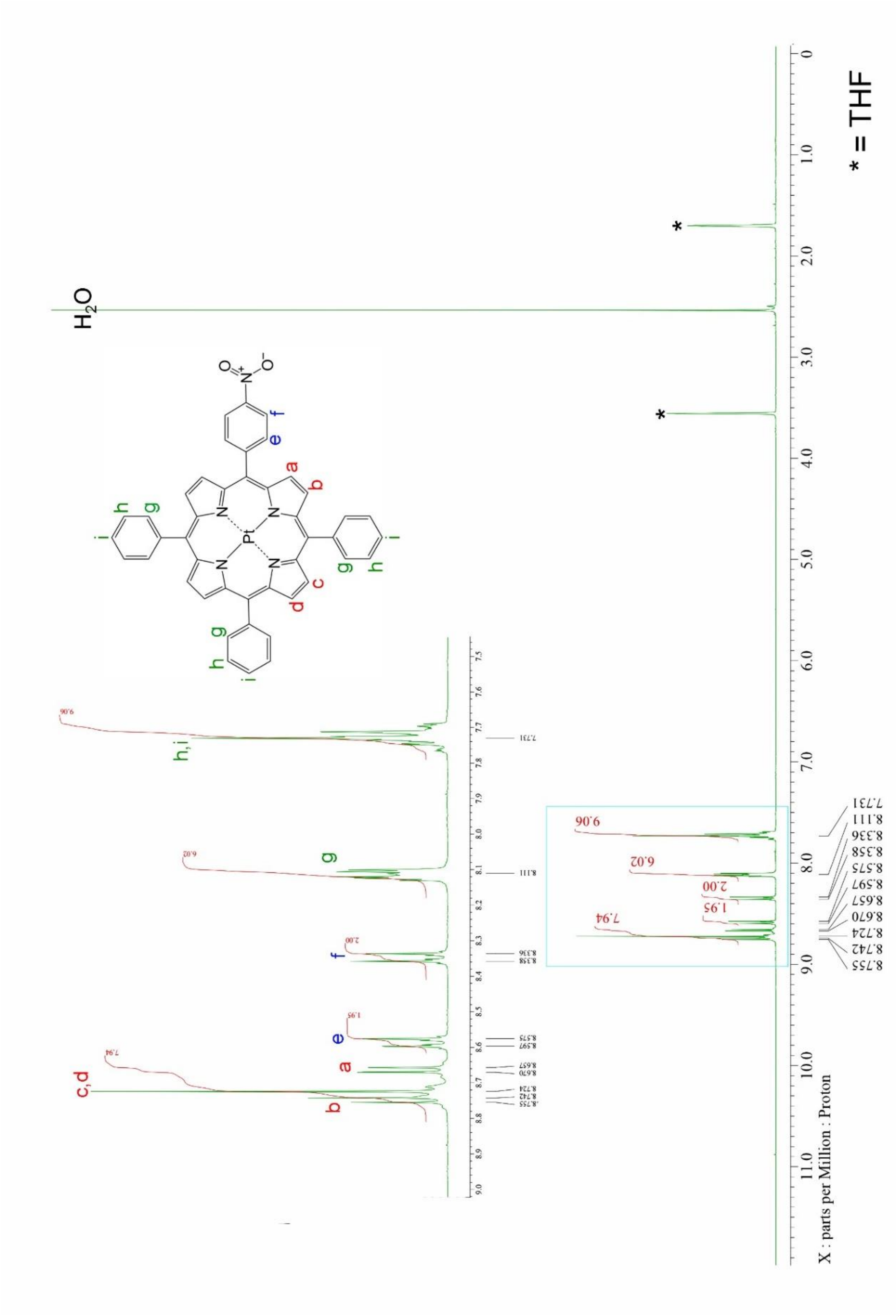

Figure S18. ^{1}H NMR spectrum of compound **1** in THF-d_8.

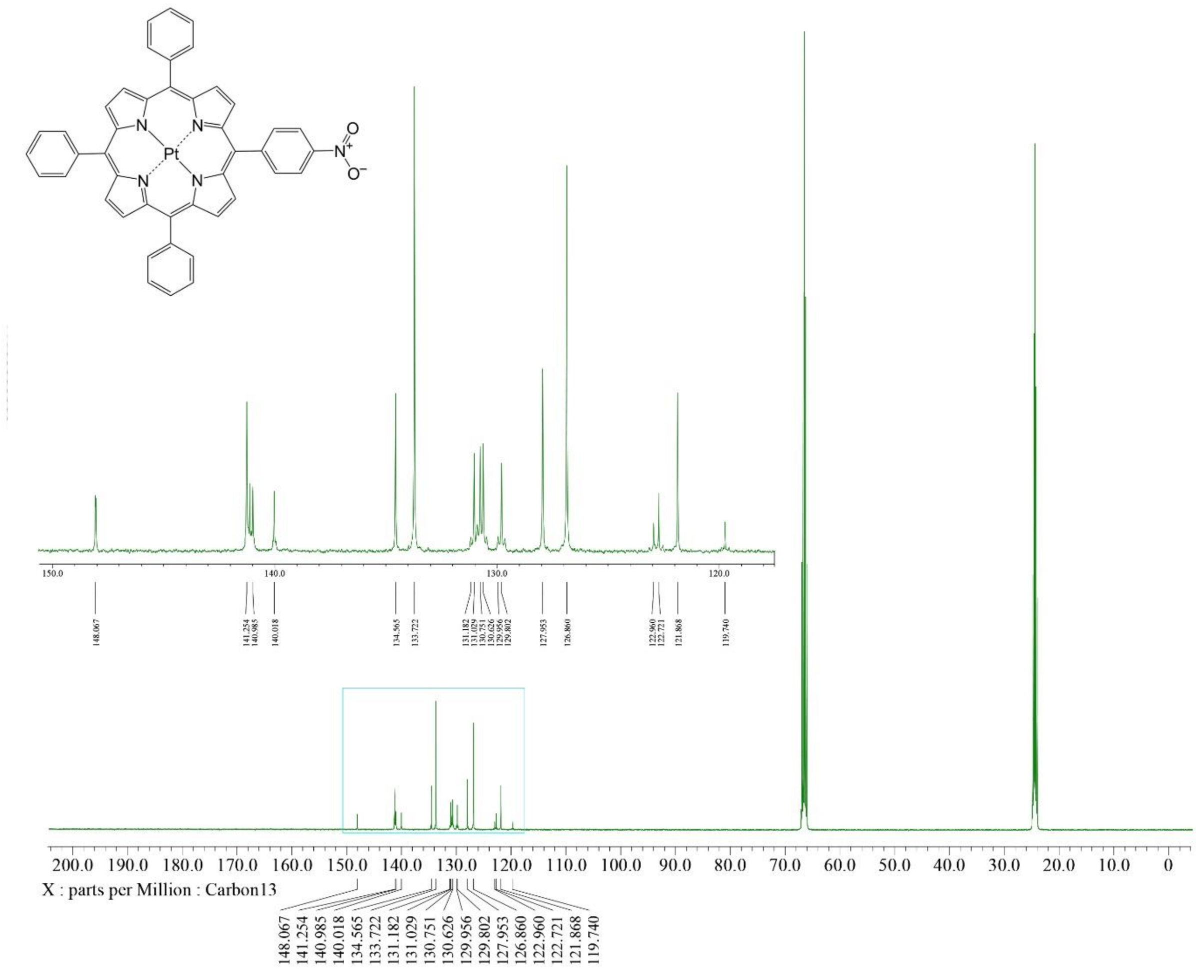

Figure S19. ^{13}C NMR spectrum of compound **1** in THF-d_8.

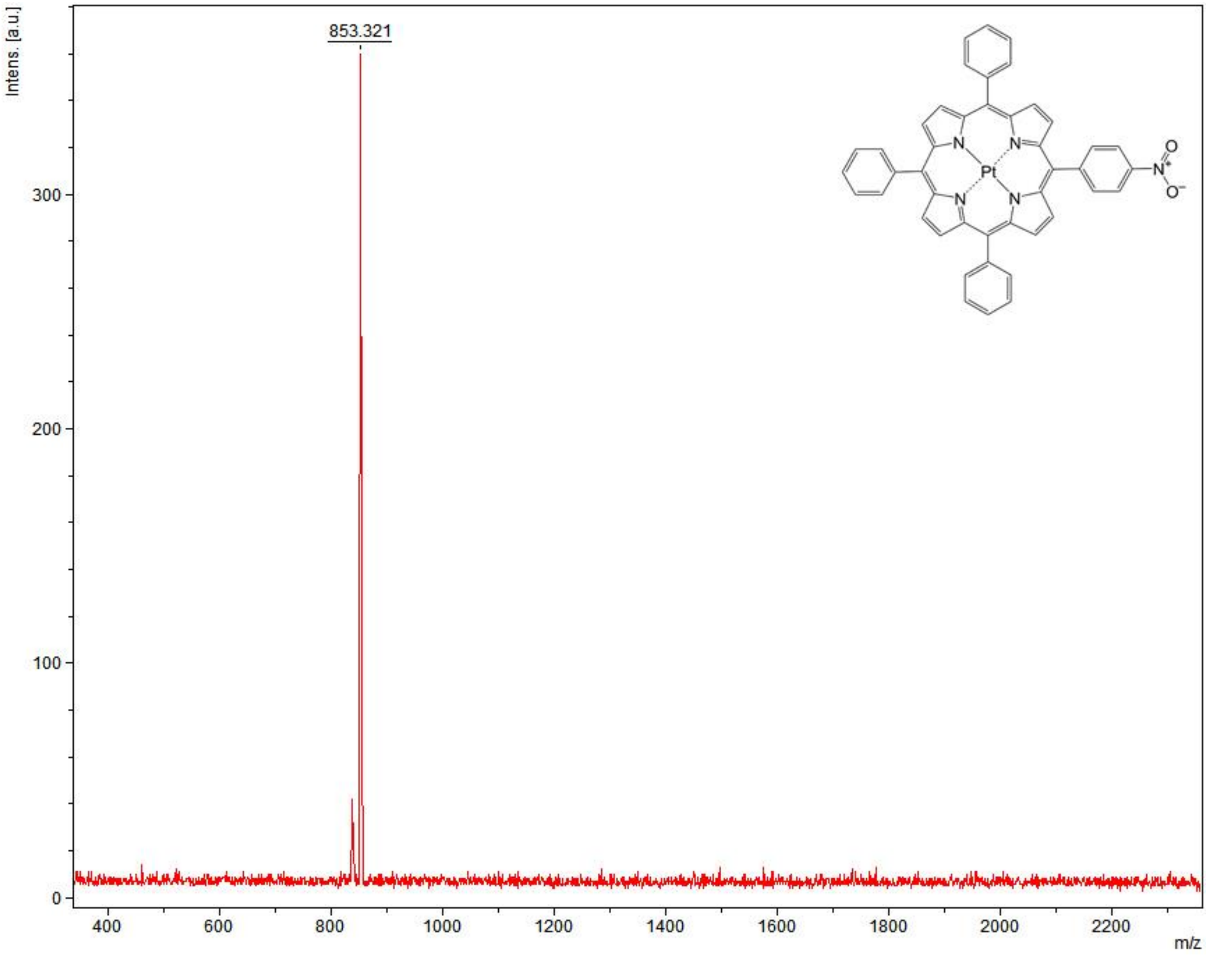

Figure S20. MALDI-TOF-MS spectrum of compound **1** using dithranol as matrix.

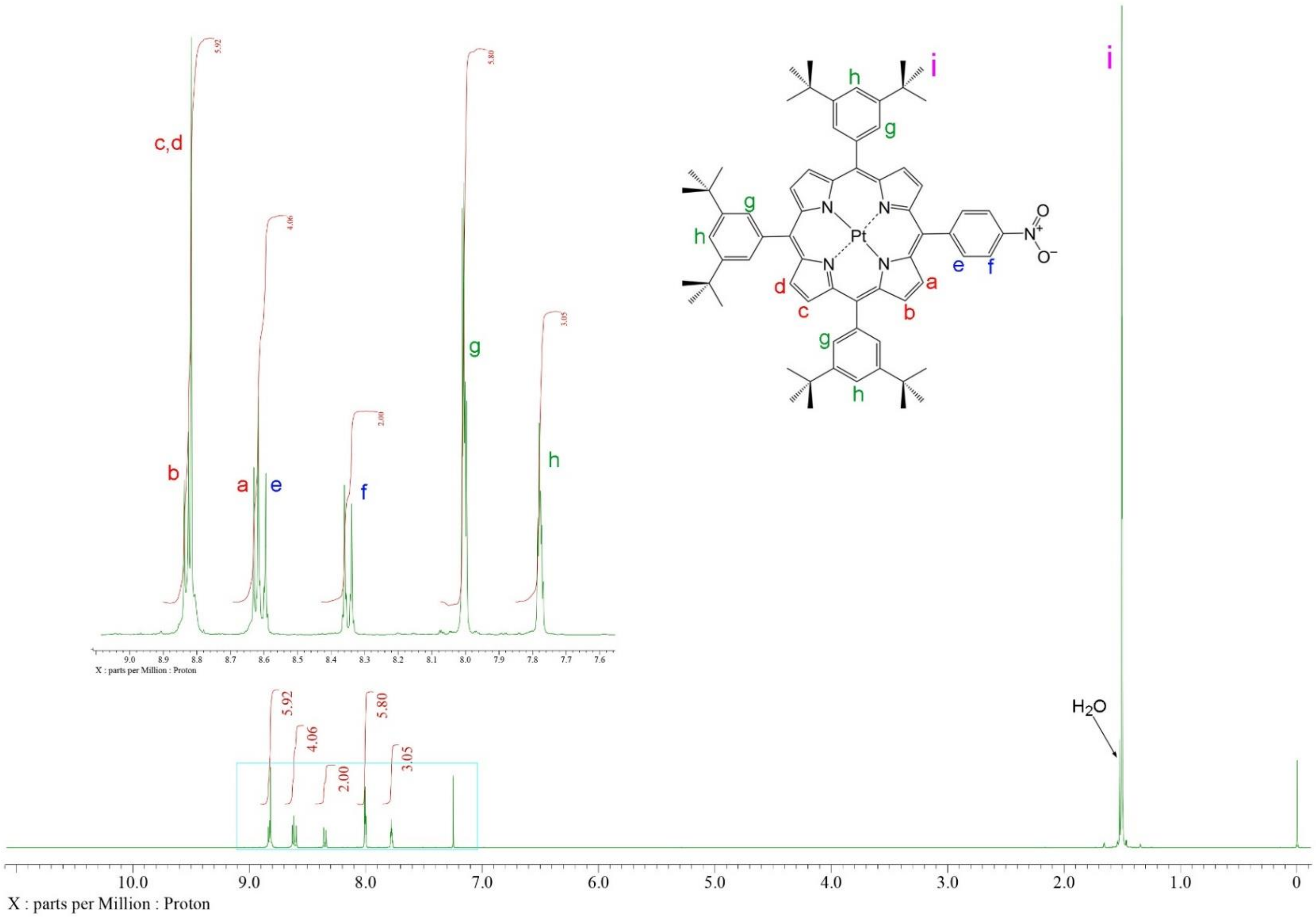

Figure S21. ^{1}H NMR spectrum of compound **2** in $CDCl_3$.

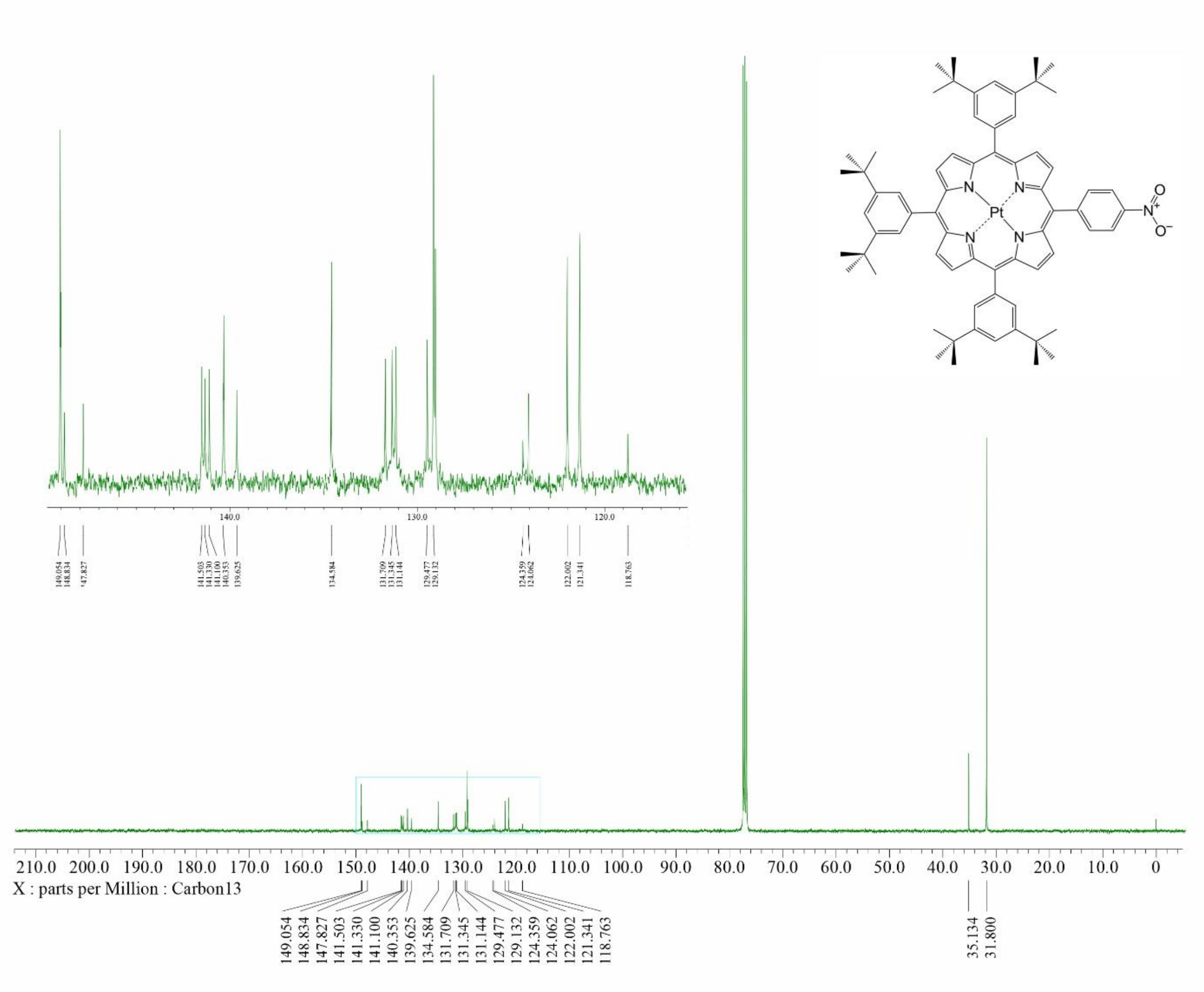

Figure S22. ^{13}C NMR spectrum of compound **2** in $CDCl_3$.

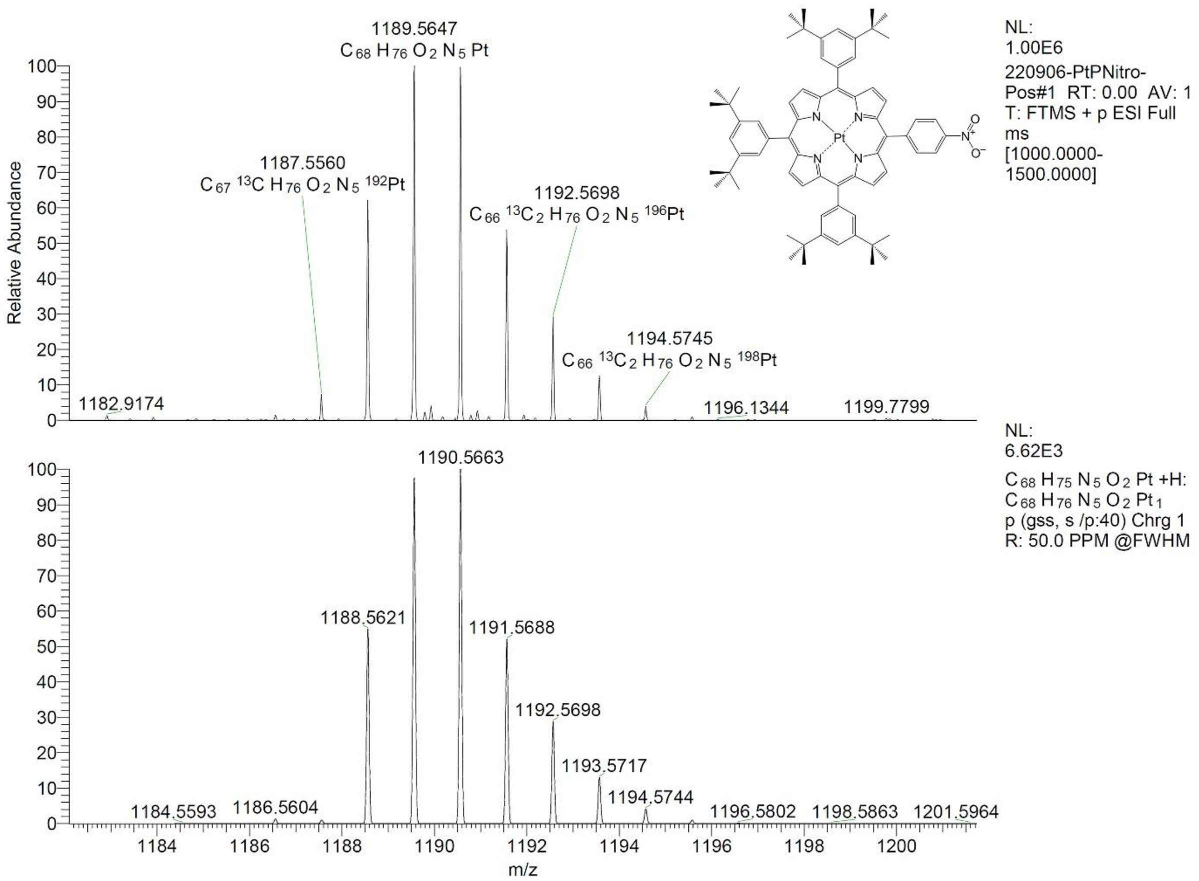

Figure S23. ESI-TOF-MS spectrum of compound **2**.
