# Supplementary material for: On-Surface Synthesis of Azobenzene-Linked Porphyrin Derivatives
Source: J Phys Chem Lett. 2025 Nov 6;16(45):11833–41. doi: 10.1021/acs.jpclett.5c03174 (PMC12621240; doi:10.1021/acs.jpclett.5c03174)
Supplement: Supplementary file 2 [file jz5c03174_si_002.pdf]

Coordinates of the calculated structures.

5-(4-Nitrophenyl)-10,15,20-triphenylporphyrin platinum (II) (**1**) on Au(111) Surface

Pt	6.662405	14.555208	2.820676
C	3.171835	12.001115	2.425719
C	4.193215	11.000079	2.398447
C	5.440183	11.663332	2.751168
N	5.217552	13.066222	2.900600
C	3.807458	13.261942	2.736780
C	4.219972	17.898251	3.937184
C	3.247948	16.897004	3.928798
C	3.843260	15.725875	3.285554
N	5.200963	15.979651	2.972173
C	5.414180	17.346133	3.294532
C	3.114926	14.522322	2.988981
C	10.136487	17.116967	2.403059
C	9.116127	18.117132	2.412250
C	7.875570	17.446762	2.756652
N	8.101085	16.045454	2.894187
C	9.506108	15.850088	2.698640
C	6.594026	18.107412	2.990558
C	9.127411	11.189102	3.815185
C	10.097371	12.195554	3.814386
C	9.492444	13.372640	3.200823
N	8.132189	13.119217	2.885571
C	7.923371	11.749169	3.204620
C	6.719904	10.997683	2.966143
C	10.209502	14.590744	2.947709
C	6.451761	19.598535	2.958460
C	1.631100	14.635076	2.904128
C	6.827322	9.506063	2.947811
C	11.694059	14.519063	2.920017
C	1.024488	15.902402	2.523116
C	-0.401051	16.074636	2.544647

C	-1.264024	14.941847	2.648100
C	-0.672869	13.652695	2.869325
C	0.730740	13.521483	3.089091
C	5.163880	20.182568	2.641881
C	4.962707	21.593402	2.627635
C	6.079558	22.482438	2.736173
C	7.370475	21.926440	3.000275
C	7.540782	20.519927	3.184645
C	12.371537	13.291425	2.541865
C	13.802512	13.187541	2.520479
C	13.937611	15.623923	3.034227
C	12.531234	15.672117	3.189933
C	8.080462	8.861000	2.604202
C	8.208646	7.439722	2.596874
C	7.052823	6.605274	2.735520
C	5.802676	7.224391	3.053619
C	5.711893	8.636273	3.235572
C	14.584550	14.368728	2.769865
N	16.018023	14.350608	2.621586
O	16.595192	15.540550	2.313369
O	16.592184	13.158666	2.327095
H	2.072628	11.834186	2.196857
H	4.023119	9.884951	2.285987
H	4.167620	18.949201	4.382741
H	2.188505	16.911686	4.358352
H	11.233887	17.284114	2.154079
H	9.283129	19.228858	2.262621
H	9.184547	10.139665	4.261954
H	11.152893	12.192179	4.252672
H	1.687188	16.819003	2.373760
H	-0.842668	17.120352	2.342749
H	-2.403234	15.048020	2.514207
H	-1.342834	12.714538	2.913025
H	1.133810	12.533943	3.490517

H	4.286277	19.492077	2.407508
H	3.916747	22.012013	2.384088
H	5.944292	23.618465	2.604171
H	8.286354	22.625084	3.076902
H	8.536197	20.129770	3.581890
H	11.756664	12.356792	2.312696
H	14.355195	12.196607	2.323630
H	14.620545	16.543582	3.167964
H	12.052543	16.621568	3.597409
H	8.992347	9.504410	2.374020
H	9.232223	6.969952	2.351819
H	7.129867	5.462653	2.615236
H	4.859088	6.572795	3.191802
H	4.763762	9.087880	3.677777
Au	7.194811	10.800209	0.000000
Au	10.072736	15.784920	0.000000
Au	14.389623	23.261988	0.000000
Au	-5.755849	23.261988	0.000000
Au	-8.633774	23.261988	0.000000
Au	15.828585	20.769632	0.000000
Au	11.511698	13.292564	0.000000
Au	8.633774	8.307853	0.000000
Au	17.267548	18.277275	0.000000
Au	-10.072736	20.769632	0.000000
Au	12.950661	10.800209	0.000000
Au	18.706511	15.784920	0.000000
Au	-4.316887	20.769632	0.000000
Au	10.072736	5.815497	0.000000
Au	20.145472	13.292564	0.000000
Au	14.389623	8.307853	0.000000
Au	1.438962	5.815497	0.000000
Au	21.584435	10.800209	0.000000
Au	-7.194811	20.769632	0.000000
Au	15.828585	5.815497	0.000000

Au	23.023397	8.307853	0.000000
Au	11.511698	3.323141	0.000000
Au	-2.877925	18.277275	0.000000
Au	24.462359	5.815497	0.000000
Au	17.267548	3.323141	0.000000
Au	-5.755849	13.292564	0.000000
Au	25.901321	3.323141	0.000000
Au	12.950661	0.830785	0.000000
Au	18.706511	0.830785	0.000000
Au	27.340284	0.830785	0.000000
Au	-2.877925	8.307853	0.000000
Au	-1.438962	15.784920	0.000000
Au	11.511698	23.261988	0.000000
Au	2.877925	23.261988	0.000000
Au	-2.877925	23.261988	0.000000
Au	12.950661	20.769632	0.000000
Au	-5.755849	18.277275	0.000000
Au	4.316887	20.769632	0.000000
Au	14.389623	18.277275	0.000000
Au	2.877925	3.323141	0.000000
Au	-1.438962	20.769632	0.000000
Au	15.828585	15.784920	0.000000
Au	5.755849	18.277275	0.000000
Au	0.000000	13.292564	0.000000
Au	17.267548	13.292564	0.000000
Au	-8.633774	18.277275	0.000000
Au	7.194811	15.784920	0.000000
Au	18.706511	10.800209	0.000000
Au	0.000000	18.277275	0.000000
Au	-4.316887	15.784920	0.000000
Au	20.145472	8.307853	0.000000
Au	8.633774	13.292564	0.000000
Au	1.438962	10.800209	0.000000
Au	21.584435	5.815497	0.000000

Au	1.438962	15.784920	0.000000
Au	10.072736	10.800209	0.000000
Au	23.023397	3.323141	0.000000
Au	0.000000	3.323141	0.000000
Au	4.316887	0.830785	0.000000
Au	24.462359	0.830785	0.000000
Au	11.511698	8.307853	0.000000
Au	2.877925	13.292564	0.000000
Au	8.633774	23.261988	0.000000
Au	2.877925	8.307853	0.000000
Au	12.950661	5.815497	0.000000
Au	10.072736	20.769632	0.000000
Au	-2.877925	13.292564	0.000000
Au	4.316887	10.800209	0.000000
Au	11.511698	18.277275	0.000000
Au	14.389623	3.323141	0.000000
Au	-4.316887	10.800209	0.000000
Au	12.950661	15.784920	0.000000
Au	4.316887	5.815497	0.000000
Au	15.828585	0.830785	0.000000
Au	14.389623	13.292564	0.000000
Au	5.755849	8.307853	0.000000
Au	-7.194811	15.784920	0.000000
Au	15.828585	10.800209	0.000000
Au	0.000000	23.261988	0.000000
Au	-1.438962	10.800209	0.000000
Au	17.267548	8.307853	0.000000
Au	7.194811	5.815497	0.000000
Au	1.438962	20.769632	0.000000
Au	18.706511	5.815497	0.000000
Au	5.755849	3.323141	0.000000
Au	-11.511698	23.261988	0.000000
Au	20.145472	3.323141	0.000000
Au	2.877925	18.277275	0.000000

Au	8.633774	3.323141	0.000000
Au	21.584435	0.830785	0.000000
Au	1.438962	0.830785	0.000000
Au	4.316887	15.784920	0.000000
Au	5.755849	23.261988	0.000000
Au	7.194811	0.830785	0.000000
Au	10.072736	0.830785	0.000000
Au	7.194811	20.769632	0.000000
Au	5.755849	13.292564	0.000000
Au	0.000000	8.307853	0.000000
Au	8.633774	18.277275	0.000000
Au	-1.438962	5.815497	0.000000
Au	0.000000	6.646282	-2.349816
Au	12.950661	24.092773	-2.349816
Au	-1.438962	24.092773	-2.349816
Au	14.389623	21.600416	-2.349816
Au	0.000000	11.630994	-2.349816
Au	15.828585	19.108061	-2.349816
Au	0.000000	21.600416	-2.349816
Au	17.267548	16.615706	-2.349816
Au	-1.438962	4.153926	-2.349816
Au	18.706511	14.123350	-2.349816
Au	1.438962	19.108061	-2.349816
Au	20.145472	11.630994	-2.349816
Au	1.438962	9.138637	-2.349816
Au	21.584435	9.138637	-2.349816
Au	2.877925	16.615706	-2.349816
Au	23.023397	6.646282	-2.349816
Au	1.438962	4.153926	-2.349816
Au	24.462359	4.153926	-2.349816
Au	4.316887	14.123350	-2.349816
Au	25.901321	1.661571	-2.349816
Au	2.877925	6.646282	-2.349816
Au	10.072736	24.092773	-2.349816

Au	5.755849	11.630994	-2.349816
Au	11.511698	21.600416	-2.349816
Au	-7.194811	14.123350	-2.349816
Au	12.950661	19.108061	-2.349816
Au	7.194811	9.138637	-2.349816
Au	14.389623	16.615706	-2.349816
Au	4.316887	4.153926	-2.349816
Au	15.828585	14.123350	-2.349816
Au	8.633774	6.646282	-2.349816
Au	17.267548	11.630994	-2.349816
Au	2.877925	1.661571	-2.349816
Au	18.706511	9.138637	-2.349816
Au	10.072736	4.153926	-2.349816
Au	20.145472	6.646282	-2.349816
Au	5.755849	1.661571	-2.349816
Au	21.584435	4.153926	-2.349816
Au	11.511698	1.661571	-2.349816
Au	23.023397	1.661571	-2.349816
Au	-2.877925	6.646282	-2.349816
Au	7.194811	24.092773	-2.349816
Au	-4.316887	24.092773	-2.349816
Au	8.633774	21.600416	-2.349816
Au	-10.072736	24.092773	-2.349816
Au	10.072736	19.108061	-2.349816
Au	-2.877925	21.600416	-2.349816
Au	11.511698	16.615706	-2.349816
Au	-12.950661	24.092773	-2.349816
Au	12.950661	14.123350	-2.349816
Au	-1.438962	19.108061	-2.349816
Au	14.389623	11.630994	-2.349816
Au	-8.633774	21.600416	-2.349816
Au	15.828585	9.138637	-2.349816
Au	0.000000	16.615706	-2.349816
Au	17.267548	6.646282	-2.349816

Au	-5.755849	11.630994	-2.349816
Au	18.706511	4.153926	-2.349816
Au	1.438962	14.123350	-2.349816
Au	20.145472	1.661571	-2.349816
Au	-7.194811	19.108061	-2.349816
Au	4.316887	24.092773	-2.349816
Au	2.877925	11.630994	-2.349816
Au	5.755849	21.600416	-2.349816
Au	-11.511698	21.600416	-2.349816
Au	7.194811	19.108061	-2.349816
Au	4.316887	9.138637	-2.349816
Au	8.633774	16.615706	-2.349816
Au	-5.755849	16.615706	-2.349816
Au	10.072736	14.123350	-2.349816
Au	5.755849	6.646282	-2.349816
Au	11.511698	11.630994	-2.349816
Au	0.000000	1.661571	-2.349816
Au	12.950661	9.138637	-2.349816
Au	7.194811	4.153926	-2.349816
Au	14.389623	6.646282	-2.349816
Au	-4.316887	14.123350	-2.349816
Au	15.828585	4.153926	-2.349816
Au	8.633774	1.661571	-2.349816
Au	17.267548	1.661571	-2.349816
Au	-10.072736	19.108061	-2.349816
Au	1.438962	24.092773	-2.349816
Au	-7.194811	24.092773	-2.349816
Au	2.877925	21.600416	-2.349816
Au	-2.877925	11.630994	-2.349816
Au	4.316887	19.108061	-2.349816
Au	-5.755849	21.600416	-2.349816
Au	5.755849	16.615706	-2.349816
Au	-4.316887	9.138637	-2.349816
Au	7.194811	14.123350	-2.349816

Au	-4.316887	19.108061	-2.349816
Au	8.633774	11.630994	-2.349816
Au	-1.438962	9.138637	-2.349816
Au	10.072736	9.138637	-2.349816
Au	-2.877925	16.615706	-2.349816
Au	11.511698	6.646282	-2.349816
Au	-8.633774	16.615706	-2.349816
Au	12.950661	4.153926	-2.349816
Au	-1.438962	14.123350	-2.349816
Au	14.389623	1.661571	-2.349816
Au	12.950661	22.431202	-4.699631
Au	14.389623	19.938847	-4.699631
Au	15.828585	17.446491	-4.699631
Au	17.267548	14.954135	-4.699631
Au	18.706511	12.461779	-4.699631
Au	20.145472	9.969423	-4.699631
Au	21.584435	7.477067	-4.699631
Au	23.023397	4.984712	-4.699631
Au	24.462359	2.492356	-4.699631
Au	25.901321	0.000000	-4.699631
Au	10.072736	22.431202	-4.699631
Au	11.511698	19.938847	-4.699631
Au	12.950661	17.446491	-4.699631
Au	14.389623	14.954135	-4.699631
Au	15.828585	12.461779	-4.699631
Au	17.267548	9.969423	-4.699631
Au	18.706511	7.477067	-4.699631
Au	20.145472	4.984712	-4.699631
Au	21.584435	2.492356	-4.699631
Au	23.023397	0.000000	-4.699631
Au	7.194811	22.431202	-4.699631
Au	8.633774	19.938847	-4.699631
Au	10.072736	17.446491	-4.699631
Au	11.511698	14.954135	-4.699631

Au	12.950661	12.461779	-4.699631
Au	14.389623	9.969423	-4.699631
Au	15.828585	7.477067	-4.699631
Au	17.267548	4.984712	-4.699631
Au	18.706511	2.492356	-4.699631
Au	20.145472	0.000000	-4.699631
Au	4.316887	22.431202	-4.699631
Au	5.755849	19.938847	-4.699631
Au	7.194811	17.446491	-4.699631
Au	8.633774	14.954135	-4.699631
Au	10.072736	12.461779	-4.699631
Au	11.511698	9.969423	-4.699631
Au	12.950661	7.477067	-4.699631
Au	14.389623	4.984712	-4.699631
Au	15.828585	2.492356	-4.699631
Au	17.267548	0.000000	-4.699631
Au	1.438962	22.431202	-4.699631
Au	2.877925	19.938847	-4.699631
Au	4.316887	17.446491	-4.699631
Au	5.755849	14.954135	-4.699631
Au	7.194811	12.461779	-4.699631
Au	8.633774	9.969423	-4.699631
Au	10.072736	7.477067	-4.699631
Au	11.511698	4.984712	-4.699631
Au	12.950661	2.492356	-4.699631
Au	14.389623	0.000000	-4.699631
Au	-1.438962	22.431202	-4.699631
Au	0.000000	19.938847	-4.699631
Au	1.438962	17.446491	-4.699631
Au	2.877925	14.954135	-4.699631
Au	4.316887	12.461779	-4.699631
Au	5.755849	9.969423	-4.699631
Au	7.194811	7.477067	-4.699631
Au	8.633774	4.984712	-4.699631

Au	10.072736	2.492356	-4.699631
Au	11.511698	0.000000	-4.699631
Au	-4.316887	22.431202	-4.699631
Au	-2.877925	19.938847	-4.699631
Au	-1.438962	17.446491	-4.699631
Au	0.000000	14.954135	-4.699631
Au	1.438962	12.461779	-4.699631
Au	2.877925	9.969423	-4.699631
Au	4.316887	7.477067	-4.699631
Au	5.755849	4.984712	-4.699631
Au	7.194811	2.492356	-4.699631
Au	8.633774	0.000000	-4.699631
Au	-7.194811	22.431202	-4.699631
Au	-5.755849	19.938847	-4.699631
Au	-4.316887	17.446491	-4.699631
Au	-2.877925	14.954135	-4.699631
Au	-1.438962	12.461779	-4.699631
Au	0.000000	9.969423	-4.699631
Au	1.438962	7.477067	-4.699631
Au	2.877925	4.984712	-4.699631
Au	4.316887	2.492356	-4.699631
Au	5.755849	0.000000	-4.699631
Au	-10.072736	22.431202	-4.699631
Au	-8.633774	19.938847	-4.699631
Au	-7.194811	17.446491	-4.699631
Au	-5.755849	14.954135	-4.699631
Au	-4.316887	12.461779	-4.699631
Au	-2.877925	9.969423	-4.699631
Au	-1.438962	7.477067	-4.699631
Au	0.000000	4.984712	-4.699631
Au	1.438962	2.492356	-4.699631
Au	2.877925	0.000000	-4.699631
Au	-12.950661	22.431202	-4.699631
Au	-11.511698	19.938847	-4.699631

Au	-10.072736	17.446491	-4.699631
Au	-8.633774	14.954135	-4.699631
Au	-7.194811	12.461779	-4.699631
Au	-5.755849	9.969423	-4.699631
Au	-4.316887	7.477067	-4.699631
Au	-2.877925	4.984712	-4.699631
Au	-1.438962	2.492356	-4.699631
Au	0.000000	0.000000	-4.699631

5-(4-Nitrophenyl)-10,15,20-tris(3,5-di-t-butylphenyl)porphyrin platinum(II) (**2**) on Au(111) Surface

C	2.643199	10.699754	2.436800
C	3.887101	11.419146	2.673004
N	3.609713	12.820879	2.814486
C	2.197838	12.956084	2.667417
C	2.301252	17.665590	3.746689
C	1.391471	16.601142	3.716782
C	2.091282	15.457243	3.136891
N	3.443015	15.784267	2.884446
C	3.555506	17.176139	3.176781
C	1.456420	14.190625	2.881274
C	8.312752	17.157821	2.503316
C	7.261012	18.101304	2.509967
C	6.023834	17.374784	2.746477
N	6.307204	15.971583	2.873633
C	7.724379	15.843551	2.732476
C	4.718935	17.994056	2.944724
C	7.588666	11.098660	3.711581
C	8.491056	12.170966	3.764328
C	7.823818	13.331814	3.182304
N	6.479111	13.007438	2.874246
C	6.351286	11.607331	3.132046
C	5.180192	10.794374	2.892713
C	8.480227	14.605149	2.944746
C	4.562586	19.495082	2.974767
C	-0.033006	14.129952	2.874261
C	5.300569	9.293004	2.907719
C	9.969074	14.609458	2.931382
C	-0.660923	12.918473	3.309587
C	-2.036163	12.646380	3.104963
C	-2.890653	13.742938	2.752076
C	-2.331890	15.071501	2.690519
C	-0.913039	15.242208	2.568664
C	3.283653	20.136248	2.724640

C	3.103167	21.543547	2.920668
C	4.249070	22.378787	3.082243
C	5.528494	21.786897	3.276061
C	5.653156	20.367446	3.328751
C	10.711162	13.407296	2.574422
C	12.134525	13.364230	2.574444
C	12.174821	15.804712	3.071553
C	10.764263	15.787944	3.229014
C	4.186002	8.441750	3.241033
C	4.269430	7.022940	3.163686
C	5.549427	6.411572	3.013823
C	6.714867	7.214288	2.853758
C	6.558276	8.626104	2.624446
C	1.754143	22.091969	3.405045
C	6.718695	22.648466	3.700331
C	0.576881	21.134806	3.170869
C	6.489939	24.126376	3.329750
C	-2.526135	11.283485	3.615014
C	-1.503735	10.161179	3.322678
C	-3.153798	16.196932	3.334460
C	-4.659104	15.881481	3.391426
C	3.077838	6.181461	3.628389
C	1.742018	6.914901	3.387759
C	8.029638	6.603069	3.366122
C	8.232921	5.110297	2.979767
C	1.956999	22.163952	4.944051
C	6.828909	22.542113	5.236465
C	-2.624222	11.429009	5.152344
C	-2.622399	16.146385	4.799247
C	3.043649	4.761823	3.000594
C	9.292099	7.436102	3.095147
C	1.402118	23.527366	2.954422
C	8.028685	22.100769	3.101778
C	-3.907536	10.849629	3.071889

C	-2.916590	17.642369	2.862674
C	3.266973	6.008673	5.153252
C	7.814725	6.634020	4.906003
C	12.873855	14.582486	2.785493
N	14.308500	14.594267	2.633761
O	14.898058	15.791468	2.402579
O	14.886442	13.413473	2.300540
C	1.596340	11.644950	2.414700
H	2.529832	9.589370	2.243513
H	2.190957	18.699370	4.213364
H	0.346440	16.517208	4.166737
Pt	4.961470	14.396451	2.773927
H	9.402211	17.377250	2.277206
H	7.355535	19.212133	2.297813
H	7.692648	10.047954	4.146089
H	9.539513	12.197139	4.216204
H	0.499431	11.409837	2.273974
H	-0.022810	12.162887	3.854555
H	-4.027999	13.637155	2.777244
H	-0.471440	16.283621	2.450832
H	2.368168	19.510350	2.469287
H	4.132211	23.507867	3.198085
H	6.611213	19.932569	3.751436
H	10.137207	12.455729	2.315297
H	12.728791	12.403236	2.346118
H	12.820348	16.750970	3.216448
H	10.254134	16.706436	3.667859
H	3.244854	8.891342	3.679906
H	5.664245	5.293555	3.215410
H	7.467618	9.262470	2.374018
H	-0.379367	21.666096	3.524402
H	0.440328	20.866153	2.054987
H	0.715738	20.165590	3.769802
H	7.483326	24.700727	3.405091

H	6.069804	24.247208	2.260760
H	5.742865	24.589678	4.067226
H	-1.983744	9.163839	3.634338
H	-1.266862	10.088360	2.189141
H	-0.524898	10.289132	3.905358
H	-5.192619	16.821580	3.784679
H	-5.097741	15.602560	2.355689
H	-4.829313	15.020000	4.130094
H	0.884386	6.160103	3.512042
H	1.596184	7.756980	4.153187
H	1.691570	7.349184	2.319700
H	9.237486	4.957716	2.434739
H	8.239667	4.467084	3.934178
H	7.402771	4.707108	2.292786
H	0.944058	22.406911	5.430217
H	2.716809	22.992625	5.176598
H	2.366883	21.164651	5.337001
H	7.742980	23.143817	5.593523
H	6.950844	21.438670	5.531475
H	5.871798	22.954588	5.721745
H	-2.884213	10.401383	5.599359
H	-3.452824	12.185190	5.409853
H	-1.620206	11.801429	5.573372
H	-3.379605	16.688488	5.475249
H	-1.616438	16.699608	4.842242
H	-2.475906	15.052098	5.127617
H	2.963314	3.979126	3.840909
H	3.978474	4.520052	2.376940
H	2.122641	4.643926	2.320146
H	10.099155	7.081815	3.834211
H	9.117105	8.559402	3.266706
H	9.703882	7.270681	2.030858
H	0.624340	23.518191	2.095885
H	0.921501	24.077661	3.843208

H	2.311312	24.132527	2.595164
H	7.891878	21.851575	1.983341
H	8.864572	22.885781	3.199330
H	8.352154	21.144905	3.649716
H	-3.769103	10.017899	2.284646
H	-4.523130	10.410963	3.940781
H	-4.505974	11.711501	2.601016
H	-3.709548	17.947797	2.080213
H	-3.062445	18.324065	3.779095
H	-1.849656	17.809567	2.463986
H	3.380573	7.038510	5.651687
H	2.356221	5.450656	5.578760
H	4.217325	5.388953	5.338500
H	6.882896	6.015229	5.165516
H	8.742788	6.182648	5.411162
H	7.649501	7.715081	5.264346
Au	7.194811	10.800209	0.000000
Au	10.072736	15.784920	0.000000
Au	14.389623	23.261988	0.000000
Au	-5.755849	23.261988	0.000000
Au	-8.633774	23.261988	0.000000
Au	15.828585	20.769632	0.000000
Au	11.511698	13.292564	0.000000
Au	8.633774	8.307853	0.000000
Au	17.267548	18.277275	0.000000
Au	-10.072736	20.769632	0.000000
Au	12.950661	10.800209	0.000000
Au	18.706511	15.784920	0.000000
Au	-4.316887	20.769632	0.000000
Au	10.072736	5.815497	0.000000
Au	20.145472	13.292564	0.000000
Au	14.389623	8.307853	0.000000
Au	1.438962	5.815497	0.000000
Au	21.584435	10.800209	0.000000

Au	-7.194811	20.769632	0.000000
Au	15.828585	5.815497	0.000000
Au	23.023397	8.307853	0.000000
Au	11.511698	3.323141	0.000000
Au	-2.877925	18.277275	0.000000
Au	24.462359	5.815497	0.000000
Au	17.267548	3.323141	0.000000
Au	-5.755849	13.292564	0.000000
Au	25.901321	3.323141	0.000000
Au	12.950661	0.830785	0.000000
Au	18.706511	0.830785	0.000000
Au	27.340284	0.830785	0.000000
Au	-2.877925	8.307853	0.000000
Au	-1.438962	15.784920	0.000000
Au	11.511698	23.261988	0.000000
Au	2.877925	23.261988	0.000000
Au	-2.877925	23.261988	0.000000
Au	12.950661	20.769632	0.000000
Au	-5.755849	18.277275	0.000000
Au	4.316887	20.769632	0.000000
Au	14.389623	18.277275	0.000000
Au	2.877925	3.323141	0.000000
Au	-1.438962	20.769632	0.000000
Au	15.828585	15.784920	0.000000
Au	5.755849	18.277275	0.000000
Au	0.000000	13.292564	0.000000
Au	17.267548	13.292564	0.000000
Au	-8.633774	18.277275	0.000000
Au	7.194811	15.784920	0.000000
Au	18.706511	10.800209	0.000000
Au	0.000000	18.277275	0.000000
Au	-4.316887	15.784920	0.000000
Au	20.145472	8.307853	0.000000
Au	8.633774	13.292564	0.000000

Au	1.438962	10.800209	0.000000
Au	21.584435	5.815497	0.000000
Au	1.438962	15.784920	0.000000
Au	10.072736	10.800209	0.000000
Au	23.023397	3.323141	0.000000
Au	0.000000	3.323141	0.000000
Au	4.316887	0.830785	0.000000
Au	24.462359	0.830785	0.000000
Au	11.511698	8.307853	0.000000
Au	2.877925	13.292564	0.000000
Au	8.633774	23.261988	0.000000
Au	2.877925	8.307853	0.000000
Au	12.950661	5.815497	0.000000
Au	10.072736	20.769632	0.000000
Au	-2.877925	13.292564	0.000000
Au	4.316887	10.800209	0.000000
Au	11.511698	18.277275	0.000000
Au	14.389623	3.323141	0.000000
Au	-4.316887	10.800209	0.000000
Au	12.950661	15.784920	0.000000
Au	4.316887	5.815497	0.000000
Au	15.828585	0.830785	0.000000
Au	14.389623	13.292564	0.000000
Au	5.755849	8.307853	0.000000
Au	-7.194811	15.784920	0.000000
Au	15.828585	10.800209	0.000000
Au	0.000000	23.261988	0.000000
Au	-1.438962	10.800209	0.000000
Au	17.267548	8.307853	0.000000
Au	7.194811	5.815497	0.000000
Au	1.438962	20.769632	0.000000
Au	18.706511	5.815497	0.000000
Au	5.755849	3.323141	0.000000
Au	-11.511698	23.261988	0.000000

Au	20.145472	3.323141	0.000000
Au	2.877925	18.277275	0.000000
Au	8.633774	3.323141	0.000000
Au	21.584435	0.830785	0.000000
Au	1.438962	0.830785	0.000000
Au	4.316887	15.784920	0.000000
Au	5.755849	23.261988	0.000000
Au	7.194811	0.830785	0.000000
Au	10.072736	0.830785	0.000000
Au	7.194811	20.769632	0.000000
Au	5.755849	13.292564	0.000000
Au	0.000000	8.307853	0.000000
Au	8.633774	18.277275	0.000000
Au	-1.438962	5.815497	0.000000
Au	0.000000	6.646282	-2.349816
Au	12.950661	24.092773	-2.349816
Au	-1.438962	24.092773	-2.349816
Au	14.389623	21.600416	-2.349816
Au	0.000000	11.630994	-2.349816
Au	15.828585	19.108061	-2.349816
Au	0.000000	21.600416	-2.349816
Au	17.267548	16.615706	-2.349816
Au	-1.438962	4.153926	-2.349816
Au	18.706511	14.123350	-2.349816
Au	1.438962	19.108061	-2.349816
Au	20.145472	11.630994	-2.349816
Au	1.438962	9.138637	-2.349816
Au	21.584435	9.138637	-2.349816
Au	2.877925	16.615706	-2.349816
Au	23.023397	6.646282	-2.349816
Au	1.438962	4.153926	-2.349816
Au	24.462359	4.153926	-2.349816
Au	4.316887	14.123350	-2.349816
Au	25.901321	1.661571	-2.349816

Au	2.877925	6.646282	-2.349816
Au	10.072736	24.092773	-2.349816
Au	5.755849	11.630994	-2.349816
Au	11.511698	21.600416	-2.349816
Au	-7.194811	14.123350	-2.349816
Au	12.950661	19.108061	-2.349816
Au	7.194811	9.138637	-2.349816
Au	14.389623	16.615706	-2.349816
Au	4.316887	4.153926	-2.349816
Au	15.828585	14.123350	-2.349816
Au	8.633774	6.646282	-2.349816
Au	17.267548	11.630994	-2.349816
Au	2.877925	1.661571	-2.349816
Au	18.706511	9.138637	-2.349816
Au	10.072736	4.153926	-2.349816
Au	20.145472	6.646282	-2.349816
Au	5.755849	1.661571	-2.349816
Au	21.584435	4.153926	-2.349816
Au	11.511698	1.661571	-2.349816
Au	23.023397	1.661571	-2.349816
Au	-2.877925	6.646282	-2.349816
Au	7.194811	24.092773	-2.349816
Au	-4.316887	24.092773	-2.349816
Au	8.633774	21.600416	-2.349816
Au	-10.072736	24.092773	-2.349816
Au	10.072736	19.108061	-2.349816
Au	-2.877925	21.600416	-2.349816
Au	11.511698	16.615706	-2.349816
Au	-12.950661	24.092773	-2.349816
Au	12.950661	14.123350	-2.349816
Au	-1.438962	19.108061	-2.349816
Au	14.389623	11.630994	-2.349816
Au	-8.633774	21.600416	-2.349816
Au	15.828585	9.138637	-2.349816

Au	0.000000	16.615706	-2.349816
Au	17.267548	6.646282	-2.349816
Au	-5.755849	11.630994	-2.349816
Au	18.706511	4.153926	-2.349816
Au	1.438962	14.123350	-2.349816
Au	20.145472	1.661571	-2.349816
Au	-7.194811	19.108061	-2.349816
Au	4.316887	24.092773	-2.349816
Au	2.877925	11.630994	-2.349816
Au	5.755849	21.600416	-2.349816
Au	-11.511698	21.600416	-2.349816
Au	7.194811	19.108061	-2.349816
Au	4.316887	9.138637	-2.349816
Au	8.633774	16.615706	-2.349816
Au	-5.755849	16.615706	-2.349816
Au	10.072736	14.123350	-2.349816
Au	5.755849	6.646282	-2.349816
Au	11.511698	11.630994	-2.349816
Au	0.000000	1.661571	-2.349816
Au	12.950661	9.138637	-2.349816
Au	7.194811	4.153926	-2.349816
Au	14.389623	6.646282	-2.349816
Au	-4.316887	14.123350	-2.349816
Au	15.828585	4.153926	-2.349816
Au	8.633774	1.661571	-2.349816
Au	17.267548	1.661571	-2.349816
Au	-10.072736	19.108061	-2.349816
Au	1.438962	24.092773	-2.349816
Au	-7.194811	24.092773	-2.349816
Au	2.877925	21.600416	-2.349816
Au	-2.877925	11.630994	-2.349816
Au	4.316887	19.108061	-2.349816
Au	-5.755849	21.600416	-2.349816
Au	5.755849	16.615706	-2.349816

Au	-4.316887	9.138637	-2.349816
Au	7.194811	14.123350	-2.349816
Au	-4.316887	19.108061	-2.349816
Au	8.633774	11.630994	-2.349816
Au	-1.438962	9.138637	-2.349816
Au	10.072736	9.138637	-2.349816
Au	-2.877925	16.615706	-2.349816
Au	11.511698	6.646282	-2.349816
Au	-8.633774	16.615706	-2.349816
Au	12.950661	4.153926	-2.349816
Au	-1.438962	14.123350	-2.349816
Au	14.389623	1.661571	-2.349816
Au	12.950661	22.431202	-4.699631
Au	14.389623	19.938847	-4.699631
Au	15.828585	17.446491	-4.699631
Au	17.267548	14.954135	-4.699631
Au	18.706511	12.461779	-4.699631
Au	20.145472	9.969423	-4.699631
Au	21.584435	7.477067	-4.699631
Au	23.023397	4.984712	-4.699631
Au	24.462359	2.492356	-4.699631
Au	25.901321	0.000000	-4.699631
Au	10.072736	22.431202	-4.699631
Au	11.511698	19.938847	-4.699631
Au	12.950661	17.446491	-4.699631
Au	14.389623	14.954135	-4.699631
Au	15.828585	12.461779	-4.699631
Au	17.267548	9.969423	-4.699631
Au	18.706511	7.477067	-4.699631
Au	20.145472	4.984712	-4.699631
Au	21.584435	2.492356	-4.699631
Au	23.023397	0.000000	-4.699631
Au	7.194811	22.431202	-4.699631
Au	8.633774	19.938847	-4.699631

Au	10.072736	17.446491	-4.699631
Au	11.511698	14.954135	-4.699631
Au	12.950661	12.461779	-4.699631
Au	14.389623	9.969423	-4.699631
Au	15.828585	7.477067	-4.699631
Au	17.267548	4.984712	-4.699631
Au	18.706511	2.492356	-4.699631
Au	20.145472	0.000000	-4.699631
Au	4.316887	22.431202	-4.699631
Au	5.755849	19.938847	-4.699631
Au	7.194811	17.446491	-4.699631
Au	8.633774	14.954135	-4.699631
Au	10.072736	12.461779	-4.699631
Au	11.511698	9.969423	-4.699631
Au	12.950661	7.477067	-4.699631
Au	14.389623	4.984712	-4.699631
Au	15.828585	2.492356	-4.699631
Au	17.267548	0.000000	-4.699631
Au	1.438962	22.431202	-4.699631
Au	2.877925	19.938847	-4.699631
Au	4.316887	17.446491	-4.699631
Au	5.755849	14.954135	-4.699631
Au	7.194811	12.461779	-4.699631
Au	8.633774	9.969423	-4.699631
Au	10.072736	7.477067	-4.699631
Au	11.511698	4.984712	-4.699631
Au	12.950661	2.492356	-4.699631
Au	14.389623	0.000000	-4.699631
Au	-1.438962	22.431202	-4.699631
Au	0.000000	19.938847	-4.699631
Au	1.438962	17.446491	-4.699631
Au	2.877925	14.954135	-4.699631
Au	4.316887	12.461779	-4.699631
Au	5.755849	9.969423	-4.699631

Au	7.194811	7.477067	-4.699631
Au	8.633774	4.984712	-4.699631
Au	10.072736	2.492356	-4.699631
Au	11.511698	0.000000	-4.699631
Au	-4.316887	22.431202	-4.699631
Au	-2.877925	19.938847	-4.699631
Au	-1.438962	17.446491	-4.699631
Au	0.000000	14.954135	-4.699631
Au	1.438962	12.461779	-4.699631
Au	2.877925	9.969423	-4.699631
Au	4.316887	7.477067	-4.699631
Au	5.755849	4.984712	-4.699631
Au	7.194811	2.492356	-4.699631
Au	8.633774	0.000000	-4.699631
Au	-7.194811	22.431202	-4.699631
Au	-5.755849	19.938847	-4.699631
Au	-4.316887	17.446491	-4.699631
Au	-2.877925	14.954135	-4.699631
Au	-1.438962	12.461779	-4.699631
Au	0.000000	9.969423	-4.699631
Au	1.438962	7.477067	-4.699631
Au	2.877925	4.984712	-4.699631
Au	4.316887	2.492356	-4.699631
Au	5.755849	0.000000	-4.699631
Au	-10.072736	22.431202	-4.699631
Au	-8.633774	19.938847	-4.699631
Au	-7.194811	17.446491	-4.699631
Au	-5.755849	14.954135	-4.699631
Au	-4.316887	12.461779	-4.699631
Au	-2.877925	9.969423	-4.699631
Au	-1.438962	7.477067	-4.699631
Au	0.000000	4.984712	-4.699631
Au	1.438962	2.492356	-4.699631
Au	2.877925	0.000000	-4.699631

Au	-12.950661	22.431202	-4.699631
Au	-11.511698	19.938847	-4.699631
Au	-10.072736	17.446491	-4.699631
Au	-8.633774	14.954135	-4.699631
Au	-7.194811	12.461779	-4.699631
Au	-5.755849	9.969423	-4.699631
Au	-4.316887	7.477067	-4.699631
Au	-2.877925	4.984712	-4.699631
Au	-1.438962	2.492356	-4.699631
Au	0.000000	0.000000	-4.699631

Azobenzene dimer of 5-(4-nitrophenyl)-10,15,20-triphenylporphyrin platinum (II) on Au(111) Surface (heterochiral, perpendicular orientation of macrocyclic saddlings, observed experimentally)

C	-7.3513399	27.8183945	2.3774980
C	-7.7224427	26.4370893	2.3515850
C	-6.5259149	25.6706244	2.7079320
N	-5.4206418	26.5397693	2.8676570
C	-5.9239765	27.8642185	2.6807120
C	-1.6547978	29.8576142	3.7591530
C	-3.0149440	30.2055265	3.7632250
C	-3.7534674	29.0722645	3.2212500
N	-2.8674745	27.9838177	2.9825210
C	-1.5656395	28.4956994	3.2473010
C	-5.1669665	29.0865567	2.9548110
C	0.4274950	24.1958292	2.3628230
C	0.8066972	25.5648158	2.3278600
C	-0.3527687	26.3420355	2.7714190
N	-1.4519255	25.4683470	2.9815410
C	-0.9323577	24.1492251	2.7880840
C	-0.3388459	27.7687447	3.0270580
C	-5.1713896	22.1842158	3.7496640
C	-3.8270886	21.7928041	3.7364300
C	-3.0818339	22.9164632	3.2032100
N	-3.9448572	24.0111833	2.9015520
C	-5.2455761	23.5240366	3.1798160
C	-6.4886836	24.2236179	2.8995600
C	-1.6558032	22.9172315	3.0192820
C	0.9769354	28.4655345	3.0299820
C	-5.8557693	30.4014923	2.8779980
C	-7.7298458	23.4003059	2.8147740
C	-0.9098320	21.6337895	2.9909650
C	-5.1036083	31.5746132	2.4418500
C	-5.7068688	32.8784350	2.4591360
C	-7.1252087	32.9892458	2.5368970
C	-7.9025497	31.8309039	2.8676190

C	-7.2555069	30.5927409	3.1413740
C	1.1255877	29.8614071	2.6840160
C	2.4276148	30.4374382	2.5464430
C	3.6076691	29.6281642	2.6167820
C	3.4684864	28.2601796	2.9964270
C	2.1884302	27.7354940	3.3103170
C	-1.4925401	20.3823435	2.5703190
C	-0.6951515	19.1988601	2.4195270
C	1.3322832	20.4785562	3.0865580
C	0.5039208	21.5949239	3.3354800
C	-9.0567313	23.9506931	3.0738160
C	-10.2269627	23.1981312	2.8524200
C	-10.1378209	21.8026051	2.5133580
C	-8.8384244	21.1707924	2.5007490
C	-7.6802263	21.9763924	2.5288180
C	0.7450952	19.2281056	2.6272110
N	1.4602777	18.0089592	2.4577520
C	6.7765963	5.7835652	2.3275500
C	5.4115993	6.1776894	2.3757680
C	5.3963705	7.6027464	2.6705600
N	6.7329698	8.0793244	2.8477590
C	7.5817763	6.9502805	2.6516510
C	11.5810876	9.4843362	3.5843240
C	11.1450695	8.1545576	3.6477300
C	9.7719570	8.1215202	3.1521370
N	9.3300361	9.4501597	2.8873750
C	10.4606410	10.2865906	3.1057100
C	9.0126153	6.9173386	2.9208010
C	7.9158137	14.3274967	2.3036550
C	9.2905643	13.9135808	2.2798250
C	9.3172464	12.5089142	2.6886890
N	7.9897779	12.0238531	2.8401230
C	7.1355823	13.1542451	2.6428290
C	10.5176309	11.7130891	2.9025440

C	3.2101419	10.5945094	3.7193920
C	3.6404182	11.9291771	3.7421650
C	4.9691948	11.9611765	3.1548980
N	5.3915763	10.6454042	2.8102350
C	4.2814612	9.8126739	3.1219750
C	4.1997360	8.3912322	2.9129700
C	5.7112316	13.1664130	2.9103560
C	11.8313329	12.4342885	2.9163200
C	9.7279548	5.5970364	2.8927700
C	2.8515893	7.7422728	2.9296710
C	4.9773109	14.4522490	2.8913600
C	9.0890261	4.3374933	3.2041040
C	9.7623801	3.0921060	3.0309570
C	11.1590082	3.0539271	2.7235210
C	11.8479585	4.2903782	2.5210190
C	11.1204686	5.5248756	2.5134110
C	11.8636006	13.8344493	3.2752690
C	13.0051741	14.6475580	3.0522270
C	14.2627994	14.0356310	2.7238980
C	14.3136866	12.6133039	2.6046270
C	13.1065926	11.8347840	2.5646710
C	5.5735024	15.7282990	3.2726160
C	4.8478912	16.9325644	3.1695610
C	2.8129430	15.6791841	2.4725280
C	3.5783934	14.4757451	2.5104950
C	1.6559650	8.4753227	2.5726100
C	0.3717430	7.8437085	2.5394240
C	0.2517957	6.4327733	2.7625730
C	1.4171854	5.6922072	3.1401980
C	2.6697888	6.3508965	3.2838160
C	3.4484722	16.9568333	2.7841380
N	2.8000000	18.2000000	2.7850000
H	-8.0403402	28.6854301	2.1774380
H	-8.7862998	26.0881740	2.2508800

H	-0.7605598	30.4596566	4.1679560
H	-3.4967765	31.1641528	4.1475330
H	1.1041120	23.3176882	2.1140770
H	1.8512442	25.9395132	2.0904330
H	-6.0681059	21.6164460	4.1761810
H	-3.3229017	20.8518149	4.1515430
Pt	-3.4192408	26.0072011	2.8448230
H	-3.9832943	31.4617424	2.2707820
H	-5.0431470	33.8068371	2.2533730
H	-7.6605756	34.0019824	2.3787490
H	-9.0504422	31.9201320	2.9246290
H	-7.8522643	29.7400250	3.6118870
H	0.2045581	30.4983113	2.4519810
H	2.5323529	31.5557403	2.2576620
H	4.6429331	30.0614402	2.3507290
H	4.3984620	27.5686766	3.0190440
H	2.1130852	26.6830913	3.7411700
H	-2.6103483	20.3014081	2.3399220
H	-1.1985580	18.1953996	2.2004280
H	2.4597601	20.4923508	3.3088960
H	0.9684507	22.5260005	3.8071120
H	-9.1257033	24.9772080	3.5462110
H	-11.2655385	23.7041153	2.9034430
H	-11.0859033	21.1686361	2.4214080
H	-8.7439100	20.0350166	2.3315840
H	-6.6630911	21.4916187	2.3534820
H	7.1476571	4.7288149	2.1262300
H	4.5082208	5.5041569	2.2195860
H	12.5632189	9.9261208	3.9607510
H	11.6951694	7.2484467	4.0682890
Pt	7.3655218	10.0484883	2.7786680
H	7.5533235	15.4014362	2.2474750
H	10.1545175	14.6415660	2.2047940
H	2.2645696	10.1425086	4.1720390

H	3.1275252	12.8434638	4.1948660
H	8.0352950	4.3367271	3.6327510
H	9.1776527	2.1086675	3.1718230
H	11.7119275	2.0509988	2.5826330
H	12.9746586	4.3039784	2.2841880
H	11.6638973	6.4943695	2.2437300
H	10.9336780	14.2811565	3.7459040
H	12.9366565	15.7884059	3.2071100
H	15.2131777	14.6778075	2.6200130
H	15.3265275	12.1079439	2.4025600
H	13.1739378	10.7254466	2.3145330
H	6.6026059	15.7292675	3.7574740
H	5.3279003	17.9285462	3.5032430
H	1.6793553	15.6669595	2.3095690
H	3.0733898	13.4837629	2.2422220
H	1.7228914	9.5855848	2.3233230
H	-0.5627073	8.4745859	2.3058860
H	-0.7672301	5.9081457	2.6387110
H	1.3448522	4.5625560	3.3648990
H	3.5451694	5.7858048	3.7375180
Au	-2.8779250	8.3078530	0.0000000
Au	-11.5116980	28.2466980	0.0000000
Au	4.3168870	35.7237660	0.0000000
Au	-4.3168870	35.7237660	0.0000000
Au	-10.0727360	35.7237660	0.0000000
Au	5.7558490	33.2314110	0.0000000
Au	-5.7558490	23.2619880	0.0000000
Au	-2.8779250	33.2314110	0.0000000
Au	7.1948110	30.7390560	0.0000000
Au	1.4389620	5.8154970	0.0000000
Au	-8.6337740	33.2314110	0.0000000
Au	8.6337740	28.2466980	0.0000000
Au	-1.4389620	30.7390560	0.0000000
Au	-14.3896230	28.2466980	0.0000000

Au	10.0727360	25.7543430	0.0000000
Au	-4.3168870	20.7696320	0.0000000
Au	0.0000000	28.2466980	0.0000000
Au	11.5116980	23.2619880	0.0000000
Au	-7.1948110	30.7390560	0.0000000
Au	-10.0727360	25.7543430	0.0000000
Au	12.9506610	20.7696320	0.0000000
Au	1.4389620	25.7543430	0.0000000
Au	-8.6337740	18.2772750	0.0000000
Au	14.3896230	18.2772750	0.0000000
Au	-5.7558490	28.2466980	0.0000000
Au	2.8779250	23.2619880	0.0000000
Au	15.8285850	15.7849200	0.0000000
Au	-2.8779250	18.2772750	0.0000000
Au	2.8779250	3.3231410	0.0000000
Au	17.2675480	13.2925640	0.0000000
Au	4.3168870	20.7696320	0.0000000
Au	-4.3168870	25.7543430	0.0000000
Au	18.7065110	10.8002090	0.0000000
Au	-8.6337740	23.2619880	0.0000000
Au	5.7558490	18.2772750	0.0000000
Au	20.1454720	8.3078530	0.0000000
Au	-1.4389620	15.7849200	0.0000000
Au	-2.8779250	23.2619880	0.0000000
Au	21.5844350	5.8154970	0.0000000
Au	7.1948110	15.7849200	0.0000000
Au	0.0000000	3.3231410	0.0000000
Au	23.0233970	3.3231410	0.0000000
Au	-12.9506610	25.7543430	0.0000000
Au	8.6337740	13.2925640	0.0000000
Au	24.4623590	0.8307850	0.0000000
Au	-1.4389620	20.7696320	0.0000000
Au	0.0000000	13.2925640	0.0000000
Au	1.4389620	35.7237660	0.0000000

Au	10.0727360	10.8002090	0.0000000
Au	-7.1948110	20.7696320	0.0000000
Au	2.8779250	33.2314110	0.0000000
Au	0.0000000	18.2772750	0.0000000
Au	11.5116980	8.3078530	0.0000000
Au	4.3168870	30.7390560	0.0000000
Au	4.3168870	0.8307850	0.0000000
Au	1.4389620	10.8002090	0.0000000
Au	5.7558490	28.2466980	0.0000000
Au	12.9506610	5.8154970	0.0000000
Au	1.4389620	15.7849200	0.0000000
Au	7.1948110	25.7543430	0.0000000
Au	-4.3168870	10.8002090	0.0000000
Au	14.3896230	3.3231410	0.0000000
Au	8.6337740	23.2619880	0.0000000
Au	-5.7558490	18.2772750	0.0000000
Au	2.8779250	13.2925640	0.0000000
Au	10.0727360	20.7696320	0.0000000
Au	15.8285850	0.8307850	0.0000000
Au	2.8779250	8.3078530	0.0000000
Au	11.5116980	18.2772750	0.0000000
Au	-7.1948110	15.7849200	0.0000000
Au	-7.1948110	35.7237660	0.0000000
Au	12.9506610	15.7849200	0.0000000
Au	4.3168870	10.8002090	0.0000000
Au	-18.7065110	35.7237660	0.0000000
Au	14.3896230	13.2925640	0.0000000
Au	-5.7558490	33.2314110	0.0000000
Au	4.3168870	5.8154970	0.0000000
Au	15.8285850	10.8002090	0.0000000
Au	5.7558490	8.3078530	0.0000000
Au	-4.3168870	30.7390560	0.0000000
Au	17.2675480	8.3078530	0.0000000
Au	-4.3168870	15.7849200	0.0000000

Au	-11.5116980	23.2619880	0.0000000
Au	18.7065110	5.8154970	0.0000000
Au	-2.8779250	28.2466980	0.0000000
Au	7.1948110	5.8154970	0.0000000
Au	20.1454720	3.3231410	0.0000000
Au	5.7558490	3.3231410	0.0000000
Au	-1.4389620	25.7543430	0.0000000
Au	21.5844350	0.8307850	0.0000000
Au	1.4389620	0.8307850	0.0000000
Au	8.6337740	3.3231410	0.0000000
Au	-1.4389620	35.7237660	0.0000000
Au	0.0000000	23.2619880	0.0000000
Au	-2.8779250	13.2925640	0.0000000
Au	0.0000000	33.2314110	0.0000000
Au	7.1948110	0.8307850	0.0000000
Au	1.4389620	20.7696320	0.0000000
Au	1.4389620	30.7390560	0.0000000
Au	10.0727360	0.8307850	0.0000000
Au	-17.2675480	33.2314110	0.0000000
Au	2.8779250	28.2466980	0.0000000
Au	2.8779250	18.2772750	0.0000000
Au	-1.4389620	5.8154970	0.0000000
Au	4.3168870	25.7543430	0.0000000
Au	-12.9506610	35.7237660	0.0000000
Au	4.3168870	15.7849200	0.0000000
Au	5.7558490	23.2619880	0.0000000
Au	-15.8285850	35.7237660	0.0000000
Au	-1.4389620	10.8002090	0.0000000
Au	7.1948110	20.7696320	0.0000000
Au	5.7558490	13.2925640	0.0000000
Au	-11.5116980	33.2314110	0.0000000
Au	8.6337740	18.2772750	0.0000000
Au	-10.0727360	20.7696320	0.0000000
Au	7.1948110	10.8002090	0.0000000

Au	10.0727360	15.7849200	0.0000000
Au	-14.3896230	33.2314110	0.0000000
Au	-10.0727360	30.7390560	0.0000000
Au	11.5116980	13.2925640	0.0000000
Au	8.6337740	8.3078530	0.0000000
Au	-15.8285850	30.7390560	0.0000000
Au	12.9506610	10.8002090	0.0000000
Au	0.0000000	8.3078530	0.0000000
Au	10.0727360	5.8154970	0.0000000
Au	14.3896230	8.3078530	0.0000000
Au	-8.6337740	28.2466980	0.0000000
Au	-12.9506610	30.7390560	0.0000000
Au	15.8285850	5.8154970	0.0000000
Au	11.5116980	3.3231410	0.0000000
Au	-5.7558490	13.2925640	0.0000000
Au	17.2675480	3.3231410	0.0000000
Au	-7.1948110	25.7543430	0.0000000
Au	12.9506610	0.8307850	0.0000000
Au	18.7065110	0.8307850	0.0000000
Au	1.4389620	19.1080610	-2.3498160
Au	2.8779250	36.5545500	-2.3498160
Au	1.4389620	9.1386370	-2.3498160
Au	4.3168870	34.0621950	-2.3498160
Au	2.8779250	16.6157060	-2.3498160
Au	5.7558490	31.5698400	-2.3498160
Au	1.4389620	4.1539260	-2.3498160
Au	7.1948110	29.0774840	-2.3498160
Au	4.3168870	14.1233500	-2.3498160
Au	8.6337740	26.5851290	-2.3498160
Au	2.8779250	6.6462820	-2.3498160
Au	10.0727360	24.0927730	-2.3498160
Au	5.7558490	11.6309940	-2.3498160
Au	11.5116980	21.6004160	-2.3498160
Au	0.0000000	1.6615710	-2.3498160

Au	12.9506610	19.1080610	-2.3498160
Au	7.1948110	9.1386370	-2.3498160
Au	14.3896230	16.6157060	-2.3498160
Au	4.3168870	4.1539260	-2.3498160
Au	15.8285850	14.1233500	-2.3498160
Au	8.6337740	6.6462820	-2.3498160
Au	17.2675480	11.6309940	-2.3498160
Au	2.8779250	1.6615710	-2.3498160
Au	18.7065110	9.1386370	-2.3498160
Au	10.0727360	4.1539260	-2.3498160
Au	20.1454720	6.6462820	-2.3498160
Au	5.7558490	1.6615710	-2.3498160
Au	21.5844350	4.1539260	-2.3498160
Au	11.5116980	1.6615710	-2.3498160
Au	23.0233970	1.6615710	-2.3498160
Au	-10.0727360	19.1080610	-2.3498160
Au	0.0000000	36.5545500	-2.3498160
Au	-11.5116980	36.5545500	-2.3498160
Au	1.4389620	34.0621950	-2.3498160
Au	-17.2675480	36.5545500	-2.3498160
Au	2.8779250	31.5698400	-2.3498160
Au	-10.0727360	34.0621950	-2.3498160
Au	4.3168870	29.0774840	-2.3498160
Au	-20.1454720	36.5545500	-2.3498160
Au	5.7558490	26.5851290	-2.3498160
Au	-8.6337740	31.5698400	-2.3498160
Au	7.1948110	24.0927730	-2.3498160
Au	-15.8285850	34.0621950	-2.3498160
Au	8.6337740	21.6004160	-2.3498160
Au	-7.1948110	29.0774840	-2.3498160
Au	10.0727360	19.1080610	-2.3498160
Au	-4.3168870	9.1386370	-2.3498160
Au	11.5116980	16.6157060	-2.3498160
Au	-5.7558490	26.5851290	-2.3498160

Au	12.9506610	14.1233500	-2.3498160
Au	-14.3896230	31.5698400	-2.3498160
Au	14.3896230	11.6309940	-2.3498160
Au	-4.3168870	24.0927730	-2.3498160
Au	15.8285850	9.1386370	-2.3498160
Au	-18.7065110	34.0621950	-2.3498160
Au	17.2675480	6.6462820	-2.3498160
Au	-2.8779250	21.6004160	-2.3498160
Au	18.7065110	4.1539260	-2.3498160
Au	-12.9506610	29.0774840	-2.3498160
Au	20.1454720	1.6615710	-2.3498160
Au	-1.4389620	19.1080610	-2.3498160
Au	-2.8779250	36.5545500	-2.3498160
Au	-8.6337740	16.6157060	-2.3498160
Au	-1.4389620	34.0621950	-2.3498160
Au	0.0000000	16.6157060	-2.3498160
Au	0.0000000	31.5698400	-2.3498160
Au	-11.5116980	26.5851290	-2.3498160
Au	1.4389620	29.0774840	-2.3498160
Au	1.4389620	14.1233500	-2.3498160
Au	2.8779250	26.5851290	-2.3498160
Au	-17.2675480	31.5698400	-2.3498160
Au	4.3168870	24.0927730	-2.3498160
Au	2.8779250	11.6309940	-2.3498160
Au	5.7558490	21.6004160	-2.3498160
Au	-10.0727360	24.0927730	-2.3498160
Au	7.1948110	19.1080610	-2.3498160
Au	4.3168870	9.1386370	-2.3498160
Au	8.6337740	16.6157060	-2.3498160
Au	-1.4389620	4.1539260	-2.3498160
Au	10.0727360	14.1233500	-2.3498160
Au	5.7558490	6.6462820	-2.3498160
Au	11.5116980	11.6309940	-2.3498160
Au	-8.6337740	21.6004160	-2.3498160

Au 12.9506610 9.1386370 -2.3498160
Au 7.1948110 4.1539260 -2.3498160
Au 14.3896230 6.6462820 -2.3498160
Au -15.8285850 29.0774840 -2.3498160
Au 15.8285850 4.1539260 -2.3498160
Au 8.6337740 1.6615710 -2.3498160
Au 17.2675480 1.6615710 -2.3498160
Au -7.1948110 19.1080610 -2.3498160
Au -5.7558490 36.5545500 -2.3498160
Au -14.3896230 36.5545500 -2.3498160
Au -4.3168870 34.0621950 -2.3498160
Au -7.1948110 14.1233500 -2.3498160
Au -2.8779250 31.5698400 -2.3498160
Au -12.9506610 34.0621950 -2.3498160
Au -1.4389620 29.0774840 -2.3498160
Au -5.7558490 16.6157060 -2.3498160
Au 0.0000000 26.5851290 -2.3498160
Au -11.5116980 31.5698400 -2.3498160
Au 1.4389620 24.0927730 -2.3498160
Au -14.3896230 26.5851290 -2.3498160
Au 2.8779250 21.6004160 -2.3498160
Au -10.0727360 29.0774840 -2.3498160
Au 4.3168870 19.1080610 -2.3498160
Au -4.3168870 14.1233500 -2.3498160
Au 5.7558490 16.6157060 -2.3498160
Au -8.6337740 26.5851290 -2.3498160
Au 7.1948110 14.1233500 -2.3498160
Au -2.8779250 6.6462820 -2.3498160
Au 8.6337740 11.6309940 -2.3498160
Au -7.1948110 24.0927730 -2.3498160
Au 10.0727360 9.1386370 -2.3498160
Au -2.8779250 11.6309940 -2.3498160
Au 11.5116980 6.6462820 -2.3498160
Au -5.7558490 21.6004160 -2.3498160

Au	12.9506610	4.1539260	-2.3498160
Au	-12.9506610	24.0927730	-2.3498160
Au	14.3896230	1.6615710	-2.3498160
Au	-4.3168870	19.1080610	-2.3498160
Au	-8.6337740	36.5545500	-2.3498160
Au	-1.4389620	9.1386370	-2.3498160
Au	-7.1948110	34.0621950	-2.3498160
Au	-2.8779250	16.6157060	-2.3498160
Au	-5.7558490	31.5698400	-2.3498160
Au	-5.7558490	11.6309940	-2.3498160
Au	-4.3168870	29.0774840	-2.3498160
Au	-1.4389620	14.1233500	-2.3498160
Au	-2.8779250	26.5851290	-2.3498160
Au	0.0000000	6.6462820	-2.3498160
Au	-1.4389620	24.0927730	-2.3498160
Au	0.0000000	11.6309940	-2.3498160
Au	0.0000000	21.6004160	-2.3498160
Au	-11.5116980	21.6004160	-2.3498160
Au	2.8779250	34.8929830	-4.6996310
Au	4.3168870	32.4006270	-4.6996310
Au	5.7558490	29.9082700	-4.6996310
Au	7.1948110	27.4159140	-4.6996310
Au	8.6337740	24.9235570	-4.6996310
Au	10.0727360	22.4312020	-4.6996310
Au	11.5116980	19.9388470	-4.6996310
Au	12.9506610	17.4464910	-4.6996310
Au	14.3896230	14.9541350	-4.6996310
Au	15.8285850	12.4617790	-4.6996310
Au	17.2675480	9.9694230	-4.6996310
Au	18.7065110	7.4770670	-4.6996310
Au	20.1454720	4.9847120	-4.6996310
Au	21.5844350	2.4923560	-4.6996310
Au	23.0233970	0.0000000	-4.6996310
Au	0.0000000	34.8929830	-4.6996310

Au	1.4389620	32.4006270	-4.6996310
Au	2.8779250	29.9082700	-4.6996310
Au	4.3168870	27.4159140	-4.6996310
Au	5.7558490	24.9235570	-4.6996310
Au	7.1948110	22.4312020	-4.6996310
Au	8.6337740	19.9388470	-4.6996310
Au	10.0727360	17.4464910	-4.6996310
Au	11.5116980	14.9541350	-4.6996310
Au	12.9506610	12.4617790	-4.6996310
Au	14.3896230	9.9694230	-4.6996310
Au	15.8285850	7.4770670	-4.6996310
Au	17.2675480	4.9847120	-4.6996310
Au	18.7065110	2.4923560	-4.6996310
Au	20.1454720	0.0000000	-4.6996310
Au	-2.8779250	34.8929830	-4.6996310
Au	-1.4389620	32.4006270	-4.6996310
Au	0.0000000	29.9082700	-4.6996310
Au	1.4389620	27.4159140	-4.6996310
Au	2.8779250	24.9235570	-4.6996310
Au	4.3168870	22.4312020	-4.6996310
Au	5.7558490	19.9388470	-4.6996310
Au	7.1948110	17.4464910	-4.6996310
Au	8.6337740	14.9541350	-4.6996310
Au	10.0727360	12.4617790	-4.6996310
Au	11.5116980	9.9694230	-4.6996310
Au	12.9506610	7.4770670	-4.6996310
Au	14.3896230	4.9847120	-4.6996310
Au	15.8285850	2.4923560	-4.6996310
Au	17.2675480	0.0000000	-4.6996310
Au	-5.7558490	34.8929830	-4.6996310
Au	-4.3168870	32.4006270	-4.6996310
Au	-2.8779250	29.9082700	-4.6996310
Au	-1.4389620	27.4159140	-4.6996310
Au	0.0000000	24.9235570	-4.6996310

Au	1.4389620	22.4312020	-4.6996310
Au	2.8779250	19.9388470	-4.6996310
Au	4.3168870	17.4464910	-4.6996310
Au	5.7558490	14.9541350	-4.6996310
Au	7.1948110	12.4617790	-4.6996310
Au	8.6337740	9.9694230	-4.6996310
Au	10.0727360	7.4770670	-4.6996310
Au	11.5116980	4.9847120	-4.6996310
Au	12.9506610	2.4923560	-4.6996310
Au	14.3896230	0.0000000	-4.6996310
Au	-8.6337740	34.8929830	-4.6996310
Au	-7.1948110	32.4006270	-4.6996310
Au	-5.7558490	29.9082700	-4.6996310
Au	-4.3168870	27.4159140	-4.6996310
Au	-2.8779250	24.9235570	-4.6996310
Au	-1.4389620	22.4312020	-4.6996310
Au	0.0000000	19.9388470	-4.6996310
Au	1.4389620	17.4464910	-4.6996310
Au	2.8779250	14.9541350	-4.6996310
Au	4.3168870	12.4617790	-4.6996310
Au	5.7558490	9.9694230	-4.6996310
Au	7.1948110	7.4770670	-4.6996310
Au	8.6337740	4.9847120	-4.6996310
Au	10.0727360	2.4923560	-4.6996310
Au	11.5116980	0.0000000	-4.6996310
Au	-11.5116980	34.8929830	-4.6996310
Au	-10.0727360	32.4006270	-4.6996310
Au	-8.6337740	29.9082700	-4.6996310
Au	-7.1948110	27.4159140	-4.6996310
Au	-5.7558490	24.9235570	-4.6996310
Au	-4.3168870	22.4312020	-4.6996310
Au	-2.8779250	19.9388470	-4.6996310
Au	-1.4389620	17.4464910	-4.6996310
Au	0.0000000	14.9541350	-4.6996310

Au	1.4389620	12.4617790	-4.6996310
Au	2.8779250	9.9694230	-4.6996310
Au	4.3168870	7.4770670	-4.6996310
Au	5.7558490	4.9847120	-4.6996310
Au	7.1948110	2.4923560	-4.6996310
Au	8.6337740	0.0000000	-4.6996310
Au	-14.3896230	34.8929830	-4.6996310
Au	-12.9506610	32.4006270	-4.6996310
Au	-11.5116980	29.9082700	-4.6996310
Au	-10.0727360	27.4159140	-4.6996310
Au	-8.6337740	24.9235570	-4.6996310
Au	-7.1948110	22.4312020	-4.6996310
Au	-5.7558490	19.9388470	-4.6996310
Au	-4.3168870	17.4464910	-4.6996310
Au	-2.8779250	14.9541350	-4.6996310
Au	-1.4389620	12.4617790	-4.6996310
Au	0.0000000	9.9694230	-4.6996310
Au	1.4389620	7.4770670	-4.6996310
Au	2.8779250	4.9847120	-4.6996310
Au	4.3168870	2.4923560	-4.6996310
Au	5.7558490	0.0000000	-4.6996310
Au	-17.2675480	34.8929830	-4.6996310
Au	-15.8285850	32.4006270	-4.6996310
Au	-14.3896230	29.9082700	-4.6996310
Au	-12.9506610	27.4159140	-4.6996310
Au	-11.5116980	24.9235570	-4.6996310
Au	-10.0727360	22.4312020	-4.6996310
Au	-8.6337740	19.9388470	-4.6996310
Au	-7.1948110	17.4464910	-4.6996310
Au	-5.7558490	14.9541350	-4.6996310
Au	-4.3168870	12.4617790	-4.6996310
Au	-2.8779250	9.9694230	-4.6996310
Au	-1.4389620	7.4770670	-4.6996310
Au	0.0000000	4.9847120	-4.6996310

Au	1.4389620	2.4923560	-4.6996310
Au	2.8779250	0.0000000	-4.6996310
Au	-20.1454720	34.8929830	-4.6996310
Au	-18.7065110	32.4006270	-4.6996310
Au	-17.2675480	29.9082700	-4.6996310
Au	-15.8285850	27.4159140	-4.6996310
Au	-14.3896230	24.9235570	-4.6996310
Au	-12.9506610	22.4312020	-4.6996310
Au	-11.5116980	19.9388470	-4.6996310
Au	-10.0727360	17.4464910	-4.6996310
Au	-8.6337740	14.9541350	-4.6996310
Au	-7.1948110	12.4617790	-4.6996310
Au	-5.7558490	9.9694230	-4.6996310
Au	-4.3168870	7.4770670	-4.6996310
Au	-2.8779250	4.9847120	-4.6996310
Au	-1.4389620	2.4923560	-4.6996310
Au	0.0000000	0.0000000	-4.6996310

Azobenzene dimer of 5-(4-nitrophenyl)-10,15,20-triphenylporphyrin platinum (II) on Au(111) Surface (homochiral, parallel orientation of macrocyclic saddlings, NOT observed experimentally)

Pt	-3.8179545	23.5599047	2.8077760
C	-7.9213164	25.0455921	2.4653280
C	-8.1880230	23.6888695	2.7306750
C	-6.9054765	23.0042998	2.8613560
N	-5.8499930	23.9483907	2.7602650
C	-6.4696502	25.2270765	2.5151830
C	-2.5061311	27.4121840	4.0806110
C	-3.8834546	27.6667322	4.0060110
C	-4.4805591	26.5512936	3.2838460
N	-3.4957634	25.5740195	2.9917930
C	-2.2734694	26.1498721	3.3953610
C	-5.8531088	26.5015054	2.8599710
C	0.2568763	22.2011829	2.4185710
C	0.4958439	23.5954247	2.4946200
C	-0.7716475	24.2185084	2.8256010
N	-1.7888061	23.2139347	2.9578040
C	-1.1442356	21.9527757	2.7407640
C	-0.9666241	25.6364837	3.0605820
C	-5.3253392	19.5315756	2.3354820
C	-3.9201775	19.2002722	2.3983710
C	-3.2028057	20.4499618	2.6895400
N	-4.1605159	21.5249625	2.7935870
C	-5.4556267	20.9277243	2.7216640
C	-6.7392429	21.5709328	2.9464800
C	-1.7533471	20.6118638	2.8095590
C	0.1169954	26.6554756	2.9421640
C	-6.5598928	27.8118872	2.7589330
C	-7.9200190	20.6565809	3.0143680
C	-0.8346039	19.4262619	2.7771420
C	-5.7606970	28.9860675	2.4199570
C	-6.3193125	30.3099839	2.4704070
C	-7.7405776	30.4768432	2.5332660

C	-8.5641790	29.3102727	2.6754470
C	-7.9824828	28.0152854	2.8745990
C	-0.2437091	28.0253981	2.6062260
C	0.7272820	29.0727202	2.5863490
C	2.1233814	28.7630592	2.6637840
C	2.5076418	27.3966446	2.8632930
C	1.5279127	26.3775791	3.0779790
C	-1.2188650	18.0442637	2.4834480
C	-0.2854359	16.9749551	2.3942840
C	1.5432293	18.5422181	2.9120390
C	0.5680323	19.5399769	3.1688910
C	-7.7546780	19.3432458	3.5987620
C	-8.7212914	18.3127694	3.4415930
C	-10.0125491	18.6044954	2.8884660
C	-10.2856589	19.9502194	2.5083200
C	-9.2389310	20.9402815	2.4866700
C	1.1503641	17.2076826	2.4778220
N	2.0000000	16.1100000	2.2550000
H	-8.7113687	25.8292688	2.2442140
H	-9.2196876	23.2844549	2.9776010
H	-1.6694096	28.0451859	4.5373890
H	-4.4800637	28.5575196	4.4029980
H	1.0491042	21.4202139	2.1767870
H	1.4934644	24.1042114	2.2935070
H	-6.1417194	18.7451956	2.3603220
H	-3.6531694	18.1754086	2.8049970
H	-4.6281025	28.8671382	2.3404620
H	-5.6225866	31.2204132	2.3418830
H	-8.2209832	31.5218463	2.4424610
H	-9.7121347	29.4254623	2.6835450
H	-8.6614111	27.1845036	3.2529280
H	-1.3455079	28.2892317	2.4690700
H	0.3739376	30.1501907	2.3765000
H	2.9215097	29.5783103	2.4981470

H	3.6280225	27.1170804	2.8597710
H	1.8740534	25.3595459	3.4593280
H	-2.2675105	17.7316970	2.2257820
H	-0.6688929	15.8998902	2.2477060
H	2.6613968	18.7100512	3.1446010
H	0.9040768	20.4788227	3.7170160
H	-6.7768309	19.1246431	4.1424920
H	-8.4852665	17.2536844	3.8353700
H	-10.8206926	17.7873950	2.7685650
H	-11.3476684	20.2643259	2.1925430
H	-9.5142260	21.9444524	2.0445330
Pt	8.0904460	8.4861901	2.8156140
C	11.8945087	6.3934263	2.4619530
C	12.3828407	7.7016085	2.7165950
C	11.2322041	8.5680215	2.8774100
N	10.0377756	7.7855003	2.8519440
C	10.4471833	6.4318874	2.6400500
C	6.1041251	4.8856948	3.9796300
C	7.4349652	4.4367381	3.9676720
C	8.2182861	5.4436386	3.2676280
N	7.4078928	6.5611108	2.9634340
C	6.0903210	6.1775783	3.3184220
C	9.5850099	5.2667175	2.8098190
C	4.2898185	10.5203259	2.3120200
C	3.8336349	9.1570971	2.3140310
C	4.9550340	8.3310169	2.7164080
N	6.1241170	9.1458758	2.8876700
C	5.6933803	10.4946476	2.7201760
C	4.8911888	6.9114920	2.9917510
C	10.1565988	12.3180285	2.8131780
C	8.8496869	12.7685062	3.0331000
C	7.9671424	11.6050862	2.9952930
N	8.7591383	10.4236450	2.8620950
C	10.1159342	10.8538136	2.8362880

C	11.2947982	10.0208393	2.9337650
C	6.5259984	11.6811567	2.9288180
C	3.6320738	6.1371712	2.8486970
C	10.0474165	3.8525077	2.7006530
C	12.6053431	10.7478289	3.0865850
C	5.8191313	12.9934386	2.9551560
C	9.0394003	2.8230031	2.4414850
C	9.3469083	1.4289816	2.4750020
C	10.7184619	0.9886110	2.5213230
C	11.7368768	1.9865187	2.6075080
C	11.4193300	3.3878039	2.7016850
C	3.6931923	4.7278029	2.4827120
C	2.5069589	3.9122094	2.4659860
C	1.2103359	4.5202903	2.5100220
C	1.1323570	5.9435678	2.7247190
C	2.3083839	6.7096614	2.9734810
C	6.3833929	14.2711468	2.5567840
C	5.5977824	15.4521800	2.4661260
C	3.6009232	14.1516436	3.1329470
C	4.4442925	13.0518972	3.4059670
C	12.6468119	11.9903148	3.8231410
C	13.7480012	12.8894890	3.7278660
C	14.9493596	12.4974972	3.0585160
C	15.0279112	11.1587799	2.5741290
C	13.8696767	10.3210628	2.5333250
C	4.1498693	15.3909791	2.6019390
N	3.3713034	16.5196989	2.2777940
H	12.5492869	5.4931792	2.2506210
H	13.4818897	7.9565577	2.8580000
H	5.1671547	4.3890752	4.4105290
H	7.8684158	3.4664377	4.3883860
H	3.5984154	11.4201973	2.2954570
H	2.7530123	8.8433104	2.1851720
H	11.0853604	12.9649862	2.7021760

H	8.5644888	13.8299299	3.3126510
H	7.9457473	3.1322175	2.3636510
H	8.4863691	0.6744072	2.3177350
H	10.9849381	-0.1295293	2.4408260
H	12.8463260	1.6731463	2.5769870
H	12.2732072	4.0682211	3.0177470
H	4.7111423	4.2192878	2.3968500
H	2.6029561	2.7739814	2.3074050
H	0.2499543	3.8925861	2.4046210
H	0.1016931	6.4605181	2.7510780
H	2.1888697	7.7637401	3.3883640
H	7.4526699	14.3497987	2.1938160
H	6.0927381	16.4541377	2.1889930
H	2.4778418	14.1469177	3.3943470
H	4.0239357	12.1480006	3.9574900
H	11.7319445	12.2918837	4.4342300
H	13.6769605	13.9206811	4.2418850
H	15.8490801	13.2121336	2.9412560
H	16.0183569	10.7630996	2.1277530
H	13.9844454	9.3226237	1.9957280
Au	-2.8779250	8.3078530	0.0000000
Au	-11.5116980	28.2466980	0.0000000
Au	4.3168870	35.7237660	0.0000000
Au	-4.3168870	35.7237660	0.0000000
Au	-10.0727360	35.7237660	0.0000000
Au	5.7558490	33.2314110	0.0000000
Au	-5.7558490	23.2619880	0.0000000
Au	-2.8779250	33.2314110	0.0000000
Au	7.1948110	30.7390560	0.0000000
Au	1.4389620	5.8154970	0.0000000
Au	-8.6337740	33.2314110	0.0000000
Au	8.6337740	28.2466980	0.0000000
Au	-1.4389620	30.7390560	0.0000000
Au	-14.3896230	28.2466980	0.0000000

Au	10.0727360	25.7543430	0.0000000
Au	-4.3168870	20.7696320	0.0000000
Au	0.0000000	28.2466980	0.0000000
Au	11.5116980	23.2619880	0.0000000
Au	-7.1948110	30.7390560	0.0000000
Au	-10.0727360	25.7543430	0.0000000
Au	12.9506610	20.7696320	0.0000000
Au	1.4389620	25.7543430	0.0000000
Au	-8.6337740	18.2772750	0.0000000
Au	14.3896230	18.2772750	0.0000000
Au	-5.7558490	28.2466980	0.0000000
Au	2.8779250	23.2619880	0.0000000
Au	15.8285850	15.7849200	0.0000000
Au	-2.8779250	18.2772750	0.0000000
Au	2.8779250	3.3231410	0.0000000
Au	17.2675480	13.2925640	0.0000000
Au	4.3168870	20.7696320	0.0000000
Au	-4.3168870	25.7543430	0.0000000
Au	18.7065100	10.8002090	0.0000000
Au	-8.6337740	23.2619880	0.0000000
Au	5.7558490	18.2772750	0.0000000
Au	20.1454720	8.3078530	0.0000000
Au	-1.4389620	15.7849200	0.0000000
Au	-2.8779250	23.2619880	0.0000000
Au	21.5844340	5.8154970	0.0000000
Au	7.1948110	15.7849200	0.0000000
Au	0.0000000	3.3231410	0.0000000
Au	23.0233970	3.3231410	0.0000000
Au	-12.9506610	25.7543430	0.0000000
Au	8.6337740	13.2925640	0.0000000
Au	24.4623580	0.8307850	0.0000000
Au	-1.4389620	20.7696320	0.0000000
Au	0.0000000	13.2925640	0.0000000
Au	1.4389620	35.7237660	0.0000000

Au	10.0727360	10.8002090	0.0000000
Au	-7.1948110	20.7696320	0.0000000
Au	2.8779250	33.2314110	0.0000000
Au	0.0000000	18.2772750	0.0000000
Au	11.5116980	8.3078530	0.0000000
Au	4.3168870	30.7390560	0.0000000
Au	4.3168870	0.8307850	0.0000000
Au	1.4389620	10.8002090	0.0000000
Au	5.7558490	28.2466980	0.0000000
Au	12.9506610	5.8154970	0.0000000
Au	1.4389620	15.7849200	0.0000000
Au	7.1948110	25.7543430	0.0000000
Au	-4.3168870	10.8002090	0.0000000
Au	14.3896230	3.3231410	0.0000000
Au	8.6337740	23.2619880	0.0000000
Au	-5.7558490	18.2772750	0.0000000
Au	2.8779250	13.2925640	0.0000000
Au	10.0727360	20.7696320	0.0000000
Au	15.8285850	0.8307850	0.0000000
Au	2.8779250	8.3078530	0.0000000
Au	11.5116980	18.2772750	0.0000000
Au	-7.1948110	15.7849200	0.0000000
Au	-7.1948110	35.7237660	0.0000000
Au	12.9506610	15.7849200	0.0000000
Au	4.3168870	10.8002090	0.0000000
Au	-18.7065100	35.7237660	0.0000000
Au	14.3896230	13.2925640	0.0000000
Au	-5.7558490	33.2314110	0.0000000
Au	4.3168870	5.8154970	0.0000000
Au	15.8285850	10.8002090	0.0000000
Au	5.7558490	8.3078530	0.0000000
Au	-4.3168870	30.7390560	0.0000000
Au	17.2675480	8.3078530	0.0000000
Au	-4.3168870	15.7849200	0.0000000

Au	-11.5116980	23.2619880	0.0000000
Au	18.7065100	5.8154970	0.0000000
Au	-2.8779250	28.2466980	0.0000000
Au	7.1948110	5.8154970	0.0000000
Au	20.1454720	3.3231410	0.0000000
Au	5.7558490	3.3231410	0.0000000
Au	-1.4389620	25.7543430	0.0000000
Au	21.5844340	0.8307850	0.0000000
Au	1.4389620	0.8307850	0.0000000
Au	8.6337740	3.3231410	0.0000000
Au	-1.4389620	35.7237660	0.0000000
Au	0.0000000	23.2619880	0.0000000
Au	-2.8779250	13.2925640	0.0000000
Au	0.0000000	33.2314110	0.0000000
Au	7.1948110	0.8307850	0.0000000
Au	1.4389620	20.7696320	0.0000000
Au	1.4389620	30.7390560	0.0000000
Au	10.0727360	0.8307850	0.0000000
Au	-17.2675480	33.2314110	0.0000000
Au	2.8779250	28.2466980	0.0000000
Au	2.8779250	18.2772750	0.0000000
Au	-1.4389620	5.8154970	0.0000000
Au	4.3168870	25.7543430	0.0000000
Au	-12.9506610	35.7237660	0.0000000
Au	4.3168870	15.7849200	0.0000000
Au	5.7558490	23.2619880	0.0000000
Au	-15.8285850	35.7237660	0.0000000
Au	-1.4389620	10.8002090	0.0000000
Au	7.1948110	20.7696320	0.0000000
Au	5.7558490	13.2925640	0.0000000
Au	-11.5116980	33.2314110	0.0000000
Au	8.6337740	18.2772750	0.0000000
Au	-10.0727360	20.7696320	0.0000000
Au	7.1948110	10.8002090	0.0000000

Au	10.0727360	15.7849200	0.0000000
Au	-14.3896230	33.2314110	0.0000000
Au	-10.0727360	30.7390560	0.0000000
Au	11.5116980	13.2925640	0.0000000
Au	8.6337740	8.3078530	0.0000000
Au	-15.8285850	30.7390560	0.0000000
Au	12.9506610	10.8002090	0.0000000
Au	0.0000000	8.3078530	0.0000000
Au	10.0727360	5.8154970	0.0000000
Au	14.3896230	8.3078530	0.0000000
Au	-8.6337740	28.2466980	0.0000000
Au	-12.9506610	30.7390560	0.0000000
Au	15.8285850	5.8154970	0.0000000
Au	11.5116980	3.3231410	0.0000000
Au	-5.7558490	13.2925640	0.0000000
Au	17.2675480	3.3231410	0.0000000
Au	-7.1948110	25.7543430	0.0000000
Au	12.9506610	0.8307850	0.0000000
Au	18.7065100	0.8307850	0.0000000
Au	1.4389620	19.1080610	-2.3498160
Au	2.8779250	36.5545500	-2.3498160
Au	1.4389620	9.1386380	-2.3498160
Au	4.3168870	34.0621950	-2.3498160
Au	2.8779250	16.6157060	-2.3498160
Au	5.7558490	31.5698390	-2.3498160
Au	1.4389620	4.1539260	-2.3498160
Au	7.1948110	29.0774840	-2.3498160
Au	4.3168870	14.1233500	-2.3498160
Au	8.6337740	26.5851290	-2.3498160
Au	2.8779250	6.6462820	-2.3498160
Au	10.0727360	24.0927730	-2.3498160
Au	5.7558490	11.6309940	-2.3498160
Au	11.5116980	21.6004160	-2.3498160
Au	0.0000000	1.6615710	-2.3498160

Au	12.9506610	19.1080610	-2.3498160
Au	7.1948110	9.1386380	-2.3498160
Au	14.3896230	16.6157060	-2.3498160
Au	4.3168870	4.1539260	-2.3498160
Au	15.8285850	14.1233500	-2.3498160
Au	8.6337740	6.6462820	-2.3498160
Au	17.2675480	11.6309940	-2.3498160
Au	2.8779250	1.6615710	-2.3498160
Au	18.7065100	9.1386380	-2.3498160
Au	10.0727360	4.1539260	-2.3498160
Au	20.1454720	6.6462820	-2.3498160
Au	5.7558490	1.6615710	-2.3498160
Au	21.5844340	4.1539260	-2.3498160
Au	11.5116980	1.6615710	-2.3498160
Au	23.0233970	1.6615710	-2.3498160
Au	-10.0727360	19.1080610	-2.3498160
Au	0.0000000	36.5545500	-2.3498160
Au	-11.5116980	36.5545500	-2.3498160
Au	1.4389620	34.0621950	-2.3498160
Au	-17.2675480	36.5545500	-2.3498160
Au	2.8779250	31.5698390	-2.3498160
Au	-10.0727360	34.0621950	-2.3498160
Au	4.3168870	29.0774840	-2.3498160
Au	-20.1454720	36.5545500	-2.3498160
Au	5.7558490	26.5851290	-2.3498160
Au	-8.6337740	31.5698390	-2.3498160
Au	7.1948110	24.0927730	-2.3498160
Au	-15.8285850	34.0621950	-2.3498160
Au	8.6337740	21.6004160	-2.3498160
Au	-7.1948110	29.0774840	-2.3498160
Au	10.0727360	19.1080610	-2.3498160
Au	-4.3168870	9.1386380	-2.3498160
Au	11.5116980	16.6157060	-2.3498160
Au	-5.7558490	26.5851290	-2.3498160

Au	12.9506610	14.1233500	-2.3498160
Au	-14.3896230	31.5698390	-2.3498160
Au	14.3896230	11.6309940	-2.3498160
Au	-4.3168870	24.0927730	-2.3498160
Au	15.8285850	9.1386380	-2.3498160
Au	-18.7065100	34.0621950	-2.3498160
Au	17.2675480	6.6462820	-2.3498160
Au	-2.8779250	21.6004160	-2.3498160
Au	18.7065100	4.1539260	-2.3498160
Au	-12.9506610	29.0774840	-2.3498160
Au	20.1454720	1.6615710	-2.3498160
Au	-1.4389620	19.1080610	-2.3498160
Au	-2.8779250	36.5545500	-2.3498160
Au	-8.6337740	16.6157060	-2.3498160
Au	-1.4389620	34.0621950	-2.3498160
Au	0.0000000	16.6157060	-2.3498160
Au	0.0000000	31.5698390	-2.3498160
Au	-11.5116980	26.5851290	-2.3498160
Au	1.4389620	29.0774840	-2.3498160
Au	1.4389620	14.1233500	-2.3498160
Au	2.8779250	26.5851290	-2.3498160
Au	-17.2675480	31.5698390	-2.3498160
Au	4.3168870	24.0927730	-2.3498160
Au	2.8779250	11.6309940	-2.3498160
Au	5.7558490	21.6004160	-2.3498160
Au	-10.0727360	24.0927730	-2.3498160
Au	7.1948110	19.1080610	-2.3498160
Au	4.3168870	9.1386380	-2.3498160
Au	8.6337740	16.6157060	-2.3498160
Au	-1.4389620	4.1539260	-2.3498160
Au	10.0727360	14.1233500	-2.3498160
Au	5.7558490	6.6462820	-2.3498160
Au	11.5116980	11.6309940	-2.3498160
Au	-8.6337740	21.6004160	-2.3498160

Au 12.9506610 9.1386380 -2.3498160
Au 7.1948110 4.1539260 -2.3498160
Au 14.3896230 6.6462820 -2.3498160
Au -15.8285850 29.0774840 -2.3498160
Au 15.8285850 4.1539260 -2.3498160
Au 8.6337740 1.6615710 -2.3498160
Au 17.2675480 1.6615710 -2.3498160
Au -7.1948110 19.1080610 -2.3498160
Au -5.7558490 36.5545500 -2.3498160
Au -14.3896230 36.5545500 -2.3498160
Au -4.3168870 34.0621950 -2.3498160
Au -7.1948110 14.1233500 -2.3498160
Au -2.8779250 31.5698390 -2.3498160
Au -12.9506610 34.0621950 -2.3498160
Au -1.4389620 29.0774840 -2.3498160
Au -5.7558490 16.6157060 -2.3498160
Au 0.0000000 26.5851290 -2.3498160
Au -11.5116980 31.5698390 -2.3498160
Au 1.4389620 24.0927730 -2.3498160
Au -14.3896230 26.5851290 -2.3498160
Au 2.8779250 21.6004160 -2.3498160
Au -10.0727360 29.0774840 -2.3498160
Au 4.3168870 19.1080610 -2.3498160
Au -4.3168870 14.1233500 -2.3498160
Au 5.7558490 16.6157060 -2.3498160
Au -8.6337740 26.5851290 -2.3498160
Au 7.1948110 14.1233500 -2.3498160
Au -2.8779250 6.6462820 -2.3498160
Au 8.6337740 11.6309940 -2.3498160
Au -7.1948110 24.0927730 -2.3498160
Au 10.0727360 9.1386380 -2.3498160
Au -2.8779250 11.6309940 -2.3498160
Au 11.5116980 6.6462820 -2.3498160
Au -5.7558490 21.6004160 -2.3498160

Au	12.9506610	4.1539260	-2.3498160
Au	-12.9506610	24.0927730	-2.3498160
Au	14.3896230	1.6615710	-2.3498160
Au	-4.3168870	19.1080610	-2.3498160
Au	-8.6337740	36.5545500	-2.3498160
Au	-1.4389620	9.1386380	-2.3498160
Au	-7.1948110	34.0621950	-2.3498160
Au	-2.8779250	16.6157060	-2.3498160
Au	-5.7558490	31.5698390	-2.3498160
Au	-5.7558490	11.6309940	-2.3498160
Au	-4.3168870	29.0774840	-2.3498160
Au	-1.4389620	14.1233500	-2.3498160
Au	-2.8779250	26.5851290	-2.3498160
Au	0.0000000	6.6462820	-2.3498160
Au	-1.4389620	24.0927730	-2.3498160
Au	0.0000000	11.6309940	-2.3498160
Au	0.0000000	21.6004160	-2.3498160
Au	-11.5116980	21.6004160	-2.3498160
Au	2.8779250	34.8929830	-4.6996310
Au	4.3168870	32.4006270	-4.6996310
Au	5.7558490	29.9082700	-4.6996310
Au	7.1948110	27.4159140	-4.6996310
Au	8.6337740	24.9235570	-4.6996310
Au	10.0727360	22.4312020	-4.6996310
Au	11.5116980	19.9388470	-4.6996310
Au	12.9506610	17.4464910	-4.6996310
Au	14.3896230	14.9541350	-4.6996310
Au	15.8285850	12.4617790	-4.6996310
Au	17.2675480	9.9694230	-4.6996310
Au	18.7065100	7.4770670	-4.6996310
Au	20.1454720	4.9847120	-4.6996310
Au	21.5844340	2.4923560	-4.6996310
Au	23.0233970	0.0000000	-4.6996310
Au	0.0000000	34.8929830	-4.6996310

Au	1.4389620	32.4006270	-4.6996310
Au	2.8779250	29.9082700	-4.6996310
Au	4.3168870	27.4159140	-4.6996310
Au	5.7558490	24.9235570	-4.6996310
Au	7.1948110	22.4312020	-4.6996310
Au	8.6337740	19.9388470	-4.6996310
Au	10.0727360	17.4464910	-4.6996310
Au	11.5116980	14.9541350	-4.6996310
Au	12.9506610	12.4617790	-4.6996310
Au	14.3896230	9.9694230	-4.6996310
Au	15.8285850	7.4770670	-4.6996310
Au	17.2675480	4.9847120	-4.6996310
Au	18.7065100	2.4923560	-4.6996310
Au	20.1454720	0.0000000	-4.6996310
Au	-2.8779250	34.8929830	-4.6996310
Au	-1.4389620	32.4006270	-4.6996310
Au	0.0000000	29.9082700	-4.6996310
Au	1.4389620	27.4159140	-4.6996310
Au	2.8779250	24.9235570	-4.6996310
Au	4.3168870	22.4312020	-4.6996310
Au	5.7558490	19.9388470	-4.6996310
Au	7.1948110	17.4464910	-4.6996310
Au	8.6337740	14.9541350	-4.6996310
Au	10.0727360	12.4617790	-4.6996310
Au	11.5116980	9.9694230	-4.6996310
Au	12.9506610	7.4770670	-4.6996310
Au	14.3896230	4.9847120	-4.6996310
Au	15.8285850	2.4923560	-4.6996310
Au	17.2675480	0.0000000	-4.6996310
Au	-5.7558490	34.8929830	-4.6996310
Au	-4.3168870	32.4006270	-4.6996310
Au	-2.8779250	29.9082700	-4.6996310
Au	-1.4389620	27.4159140	-4.6996310
Au	0.0000000	24.9235570	-4.6996310

Au	1.4389620	22.4312020	-4.6996310
Au	2.8779250	19.9388470	-4.6996310
Au	4.3168870	17.4464910	-4.6996310
Au	5.7558490	14.9541350	-4.6996310
Au	7.1948110	12.4617790	-4.6996310
Au	8.6337740	9.9694230	-4.6996310
Au	10.0727360	7.4770670	-4.6996310
Au	11.5116980	4.9847120	-4.6996310
Au	12.9506610	2.4923560	-4.6996310
Au	14.3896230	0.0000000	-4.6996310
Au	-8.6337740	34.8929830	-4.6996310
Au	-7.1948110	32.4006270	-4.6996310
Au	-5.7558490	29.9082700	-4.6996310
Au	-4.3168870	27.4159140	-4.6996310
Au	-2.8779250	24.9235570	-4.6996310
Au	-1.4389620	22.4312020	-4.6996310
Au	0.0000000	19.9388470	-4.6996310
Au	1.4389620	17.4464910	-4.6996310
Au	2.8779250	14.9541350	-4.6996310
Au	4.3168870	12.4617790	-4.6996310
Au	5.7558490	9.9694230	-4.6996310
Au	7.1948110	7.4770670	-4.6996310
Au	8.6337740	4.9847120	-4.6996310
Au	10.0727360	2.4923560	-4.6996310
Au	11.5116980	0.0000000	-4.6996310
Au	-11.5116980	34.8929830	-4.6996310
Au	-10.0727360	32.4006270	-4.6996310
Au	-8.6337740	29.9082700	-4.6996310
Au	-7.1948110	27.4159140	-4.6996310
Au	-5.7558490	24.9235570	-4.6996310
Au	-4.3168870	22.4312020	-4.6996310
Au	-2.8779250	19.9388470	-4.6996310
Au	-1.4389620	17.4464910	-4.6996310
Au	0.0000000	14.9541350	-4.6996310

Au	1.4389620	12.4617790	-4.6996310
Au	2.8779250	9.9694230	-4.6996310
Au	4.3168870	7.4770670	-4.6996310
Au	5.7558490	4.9847120	-4.6996310
Au	7.1948110	2.4923560	-4.6996310
Au	8.6337740	0.0000000	-4.6996310
Au	-14.3896230	34.8929830	-4.6996310
Au	-12.9506610	32.4006270	-4.6996310
Au	-11.5116980	29.9082700	-4.6996310
Au	-10.0727360	27.4159140	-4.6996310
Au	-8.6337740	24.9235570	-4.6996310
Au	-7.1948110	22.4312020	-4.6996310
Au	-5.7558490	19.9388470	-4.6996310
Au	-4.3168870	17.4464910	-4.6996310
Au	-2.8779250	14.9541350	-4.6996310
Au	-1.4389620	12.4617790	-4.6996310
Au	0.0000000	9.9694230	-4.6996310
Au	1.4389620	7.4770670	-4.6996310
Au	2.8779250	4.9847120	-4.6996310
Au	4.3168870	2.4923560	-4.6996310
Au	5.7558490	0.0000000	-4.6996310
Au	-17.2675480	34.8929830	-4.6996310
Au	-15.8285850	32.4006270	-4.6996310
Au	-14.3896230	29.9082700	-4.6996310
Au	-12.9506610	27.4159140	-4.6996310
Au	-11.5116980	24.9235570	-4.6996310
Au	-10.0727360	22.4312020	-4.6996310
Au	-8.6337740	19.9388470	-4.6996310
Au	-7.1948110	17.4464910	-4.6996310
Au	-5.7558490	14.9541350	-4.6996310
Au	-4.3168870	12.4617790	-4.6996310
Au	-2.8779250	9.9694230	-4.6996310
Au	-1.4389620	7.4770670	-4.6996310
Au	0.0000000	4.9847120	-4.6996310

Au	1.4389620	2.4923560	-4.6996310
Au	2.8779250	0.0000000	-4.6996310
Au	-20.1454720	34.8929830	-4.6996310
Au	-18.7065100	32.4006270	-4.6996310
Au	-17.2675480	29.9082700	-4.6996310
Au	-15.8285850	27.4159140	-4.6996310
Au	-14.3896230	24.9235570	-4.6996310
Au	-12.9506610	22.4312020	-4.6996310
Au	-11.5116980	19.9388470	-4.6996310
Au	-10.0727360	17.4464910	-4.6996310
Au	-8.6337740	14.9541350	-4.6996310
Au	-7.1948110	12.4617790	-4.6996310
Au	-5.7558490	9.9694230	-4.6996310
Au	-4.3168870	7.4770670	-4.6996310
Au	-2.8779250	4.9847120	-4.6996310
Au	-1.4389620	2.4923560	-4.6996310
Au	0.0000000	0.0000000	-4.6996310

Azobenzene dimer of 5-(4-Nitrophenyl)-10,15,20-tris(3,5-di-t-butylphenyl)porphyrin platinum(II) (**2**) on Au(111) Surface
(heterochiral, perpendicular orientation of macrocyclic saddlings, observed experimentally)

C	8.990475	11.524034	3.157556
N	7.949963	10.603513	2.889009
C	8.453895	9.333103	3.262401
C	9.738787	9.488306	3.930793
C	10.083568	10.848975	3.852091
Pt	5.961927	11.076824	2.760976
N	3.964205	11.551493	2.867472
C	3.438633	12.830521	3.196679
C	2.106158	12.680944	3.770214
C	1.760741	11.326859	3.684560
C	2.908391	10.629844	3.118980
C	4.070202	14.105850	2.936475
C	3.187531	15.307319	2.845717
C	3.609826	16.683142	3.025448
C	2.752245	17.797605	2.765589
C	1.337811	17.609244	2.488013
C	0.883869	16.227212	2.363246
C	1.788074	15.136221	2.476550
C	2.950803	9.203517	2.921745
C	4.192439	8.477669	2.746699
C	4.304946	7.040419	2.500295
C	5.678278	6.726154	2.482704
C	6.415589	7.967854	2.695074
N	5.491910	9.056080	2.853545
C	7.844873	8.063685	2.925659
C	8.745594	6.865948	2.907469
C	10.157626	7.063805	2.682940
C	11.110016	6.024305	2.895745
C	10.644140	4.677303	3.030867
C	9.241190	4.423908	3.098649
C	8.318359	5.514295	3.143919

C	12.509557	6.412706	3.395278
C	13.666419	5.492855	2.929171
C	8.706567	3.043688	3.505660
C	7.219955	2.881053	3.086289
N	0.376018	18.624308	2.289201
N	0.988522	19.896428	2.470666
C	-0.000511	20.880119	2.582274
C	-1.447879	20.714375	2.387067
C	-2.338116	21.843893	2.537813
C	-1.863410	23.154304	2.913477
C	-0.440623	23.259872	3.209287
C	0.463012	22.217190	2.939562
C	-2.733373	24.360045	2.944338
C	-2.136855	25.661023	2.687854
N	-2.783391	26.910504	2.902684
C	-1.775746	27.904618	2.732640
C	-0.518213	27.299638	2.334712
C	-0.761936	25.888197	2.254429
Pt	-4.818799	27.265134	2.832320
N	-4.482100	29.277693	3.017158
C	-3.237266	29.864714	3.322837
C	-3.416396	31.155070	3.979620
C	-4.800155	31.382675	4.030770
C	-5.449357	30.253535	3.367608
C	-1.958115	29.310079	2.982426
C	-0.844503	30.256616	2.754091
C	-1.177250	31.550322	2.164521
C	-0.195221	32.673375	2.193778
C	1.197266	32.258417	2.417137
C	1.547200	30.935766	2.812028
C	0.516317	29.964018	3.054052
C	-6.839808	30.189790	2.990350
C	-7.571991	31.500655	2.909482
C	-8.983096	31.672283	3.137558

C	-9.581626	32.971483	3.109714
C	-8.763962	34.139632	3.022464
C	-7.345394	34.005971	2.896492
C	-6.793864	32.705533	2.698341
C	-11.057813	33.105024	3.501573
C	-11.620141	34.485673	3.083445
C	-6.431088	35.128561	3.405825
C	-4.936636	34.807813	3.230200
C	-0.610179	33.833069	3.153630
C	0.240417	35.114663	3.032920
C	2.900982	30.675668	3.482595
C	3.537421	29.324707	3.090163
C	8.987195	12.933121	2.818105
C	10.310991	13.589083	2.702160
C	11.489860	12.792417	2.353562
C	12.815336	13.287778	2.656721
C	12.990468	14.694978	2.828436
C	11.844529	15.553578	2.909178
C	10.534910	14.991583	2.922369
C	13.839541	12.345499	3.316455
C	13.591053	12.676936	4.818774
C	11.997621	16.937328	3.544115
C	12.271541	16.627711	5.038427
C	1.665901	8.436099	2.958412
C	0.373600	8.996783	2.604595
C	-0.825575	8.231159	2.789018
C	-0.729146	6.807973	2.968326
C	0.541225	6.237691	3.280228
C	1.667634	7.083425	3.417219
C	0.735006	4.765736	3.653011
C	-0.482041	3.931965	3.213492
C	-2.071349	8.924027	3.366066
C	-2.092425	10.449975	3.198532
N	6.440212	13.104637	2.831541

C	5.510983	14.194604	2.713256
C	6.240153	15.429774	2.479671
C	7.620382	15.111866	2.430457
C	7.738498	13.671487	2.655718
C	12.826540	7.905465	3.172723
C	12.384605	6.216969	4.933001
C	9.614823	1.882277	3.017745
C	8.766033	3.060788	5.054204
C	13.587468	10.835843	3.129604
C	15.322238	12.668056	3.015804
C	13.172588	17.786128	3.025930
C	10.704028	17.770893	3.446347
C	2.061432	4.263563	3.033259
C	0.906284	4.685190	5.186233
C	-3.434837	8.307577	2.977484
C	-1.862195	8.661782	4.886930
C	-4.153631	24.233763	3.166504
N	-5.131483	25.231955	2.875538
C	-6.383470	24.626614	3.133234
C	-6.186801	23.292239	3.689944
C	-4.803367	23.041971	3.687742
C	-7.683830	25.216264	2.862091
C	-7.868793	26.654032	2.723208
N	-6.852807	27.634075	2.924023
C	-7.474314	28.908729	2.751420
C	-8.873029	28.724085	2.387238
C	-9.126352	27.320517	2.398602
C	-8.828342	24.284080	2.703240
C	-10.178922	24.648391	3.007929
C	-11.264509	23.740180	2.813970
C	-11.007331	22.372352	2.453741
C	-9.646745	21.889887	2.321413
C	-8.600781	22.918359	2.206542
C	-12.579413	24.053292	3.535949

C	-12.436710	23.349922	4.910590
C	-9.282330	20.661363	3.189060
C	-9.481885	21.275128	4.611034
C	-11.902217	31.907383	2.980545
C	-11.083454	33.039278	5.049091
C	-6.759387	36.559316	2.919660
C	-6.709316	35.119392	4.935200
C	-12.776142	25.567113	3.745382
C	-13.853361	23.489153	2.883259
C	-10.238300	19.459806	3.040482
C	-7.834161	20.146183	3.071803
C	-2.093201	34.241101	3.097155
C	-0.290447	33.193476	4.544713
C	3.912778	31.814422	3.280834
C	2.584900	30.626175	4.999076
H	11.001320	11.379224	4.269258
H	3.444193	6.323655	2.317567
H	6.117583	5.702636	2.245639
H	1.514978	13.545934	4.227022
H	0.862971	10.775477	4.125617
H	8.451842	15.861551	2.259182
H	5.798003	16.456259	2.277510
H	10.286965	8.624279	4.430107
H	10.530708	8.116953	2.476553
H	11.385471	3.817164	3.188885
H	7.270289	5.287736	3.512366
H	2.603517	6.668497	3.911733
H	-1.669252	6.168246	3.063312
H	0.278118	10.108142	2.367128
H	4.634741	16.900510	3.469704
H	3.119690	18.882462	2.886362
H	-0.241931	16.026169	2.185267
H	1.375625	14.086983	2.302523
H	11.350588	11.671029	2.263884

H	14.012373	15.110119	3.127104
H	9.711142	15.619147	3.380750
H	2.071432	3.113428	2.976612
H	2.192361	4.674601	1.964898
H	2.955534	4.615955	3.661391
H	-4.005917	9.002791	2.256928
H	-3.325382	7.271879	2.485029
H	-4.067868	8.191040	3.933161
H	13.932733	8.092610	3.409722
H	12.638971	8.247235	2.086736
H	12.187903	8.554033	3.864189
H	8.986904	1.051711	2.523907
H	10.392374	2.241344	2.249354
H	10.178189	1.427211	3.914395
H	14.218035	10.283347	3.915138
H	12.501863	10.522584	3.283802
H	13.943139	10.458559	2.095628
H	12.821900	18.485320	2.179552
H	13.522448	18.443237	3.893108
H	14.066945	17.171168	2.644483
H	1.175230	3.603557	5.479863
H	-0.067025	5.009421	5.709053
H	1.760344	5.385915	5.507130
H	-2.624025	9.289886	5.477594
H	-0.784495	8.934379	5.185321
H	-2.021024	7.541514	5.085362
H	13.335487	6.638731	5.424355
H	12.290330	5.093932	5.155581
H	11.451909	6.754777	5.334677
H	8.360105	2.062869	5.461682
H	8.136592	3.941296	5.441873
H	9.860881	3.209704	5.373787
H	15.927333	12.533807	3.986567
H	15.470553	13.741243	2.626935

H	15.745375	11.946654	2.218576
H	10.971963	18.867236	3.668488
H	9.931378	17.427649	4.221651
H	10.256312	17.708228	2.383128
H	-0.194690	2.822906	3.109657
H	-1.322624	4.040997	3.991179
H	-0.888001	4.298970	2.199676
H	-2.214045	10.777484	2.091064
H	-3.010016	10.836643	3.773179
H	-1.150888	10.935085	3.642440
H	14.358550	6.058358	2.202157
H	14.291782	5.187842	3.847301
H	13.280932	4.535610	2.417423
H	6.985403	1.799086	2.770343
H	6.537195	3.166207	3.965326
H	6.968718	3.564622	2.194576
H	12.464508	12.717475	5.053063
H	14.119951	11.893547	5.476775
H	14.020004	13.720996	5.030994
H	13.318501	16.160937	5.134626
H	12.219836	17.606544	5.644950
H	11.491828	15.873568	5.422539
H	-10.140703	26.842836	2.201531
H	-2.556049	31.812866	4.345527
H	-5.383935	32.241342	4.501314
H	0.048364	25.101491	2.151646
H	0.497797	27.801904	2.250728
H	-7.019016	22.631190	4.099544
H	-4.233046	22.139097	4.095452
H	-9.657099	29.533396	2.228751
H	-9.637485	30.807871	3.483325
H	-9.244073	35.170608	3.155932
H	-5.676142	32.607414	2.521727
H	-2.276986	31.821572	2.233509

H	2.003725	33.061719	2.520437
H	0.776919	29.021929	3.634751
H	-3.454014	21.703219	2.314494
H	-1.868633	19.644044	2.325112
H	1.594494	22.351340	3.120494
H	-0.055739	24.210197	3.703480
H	-10.379245	25.627656	3.542780
H	-11.845159	21.618178	2.635561
H	-7.527465	22.559214	2.226073
H	-2.317117	34.858484	4.037583
H	-2.319066	34.917588	2.190062
H	-2.825963	33.369281	3.083269
H	4.929792	31.442008	3.666930
H	4.028541	32.110451	2.171907
H	3.597320	32.730891	3.894482
H	-12.880349	32.267449	2.488625
H	-11.323353	31.301957	2.194398
H	-12.159283	31.205305	3.855666
H	-6.002314	36.892354	2.116562
H	-7.828531	36.653047	2.498860
H	-6.652506	37.276602	3.815455
H	-13.864445	25.733860	4.075972
H	-12.081959	25.964154	4.566579
H	-12.597071	26.134731	2.754223
H	-9.810612	18.685104	2.302407
H	-10.354724	18.951490	4.062450
H	-11.272137	19.753467	2.649803
H	-0.896246	33.717178	5.371684
H	0.830180	33.347421	4.737082
H	-0.483684	32.060076	4.544358
H	3.582966	30.635866	5.571107
H	2.006684	29.669283	5.257844
H	1.945983	31.537028	5.294345
H	-12.174444	33.140006	5.401763

H	-10.444006	33.892601	5.479861
H	-10.652693	32.029452	5.389626
H	-5.929323	35.794842	5.443406
H	-6.651727	34.048197	5.350563
H	-7.766980	35.529548	5.118393
H	-14.642443	23.359876	3.711428
H	-13.680820	22.466458	2.385157
H	-14.272405	24.250204	2.122782
H	-7.651559	19.470891	3.985316
H	-7.019621	20.952679	3.065809
H	-7.706699	19.482298	2.136661
H	-0.218415	35.857373	2.279320
H	0.254304	35.616307	4.068452
H	1.326168	34.898740	2.723469
H	4.255765	29.447151	2.194044
H	4.148846	28.942284	3.987787
H	2.738338	28.538412	2.824198
H	-12.757076	34.425299	2.909976
H	-11.405105	35.254415	3.911331
H	-11.129569	34.864971	2.112669
H	-4.327169	35.746525	3.479794
H	-4.618704	33.973004	3.940298
H	-4.664043	34.489654	2.158391
H	-11.395456	23.547777	5.354804
H	-13.264548	23.736272	5.611245
H	-12.575743	22.221598	4.753271
H	-10.605220	21.423954	4.792192
H	-9.036663	20.570370	5.404941
H	-9.000294	22.318656	4.670655
Au	-2.877925	8.307853	0.000000
Au	-11.511698	28.246698	0.000000
Au	4.316887	35.723766	0.000000
Au	-4.316887	35.723766	0.000000
Au	-10.072736	35.723766	0.000000

Au	5.755849	33.231411	0.000000
Au	-5.755849	23.261988	0.000000
Au	-2.877925	33.231411	0.000000
Au	7.194811	30.739056	0.000000
Au	1.438962	5.815497	0.000000
Au	-8.633774	33.231411	0.000000
Au	8.633774	28.246698	0.000000
Au	-1.438962	30.739056	0.000000
Au	-14.389623	28.246698	0.000000
Au	10.072736	25.754343	0.000000
Au	-4.316887	20.769632	0.000000
Au	0.000000	28.246698	0.000000
Au	11.511698	23.261988	0.000000
Au	-7.194811	30.739056	0.000000
Au	-10.072736	25.754343	0.000000
Au	12.950661	20.769632	0.000000
Au	1.438962	25.754343	0.000000
Au	-8.633774	18.277275	0.000000
Au	14.389623	18.277275	0.000000
Au	-5.755849	28.246698	0.000000
Au	2.877925	23.261988	0.000000
Au	15.828585	15.784920	0.000000
Au	-2.877925	18.277275	0.000000
Au	2.877925	3.323141	0.000000
Au	17.267548	13.292564	0.000000
Au	4.316887	20.769632	0.000000
Au	-4.316887	25.754343	0.000000
Au	18.706511	10.800209	0.000000
Au	-8.633774	23.261988	0.000000
Au	5.755849	18.277275	0.000000
Au	20.145472	8.307853	0.000000
Au	-1.438962	15.784920	0.000000
Au	-2.877925	23.261988	0.000000
Au	21.584435	5.815497	0.000000

Au	7.194811	15.784920	0.000000
Au	0.000000	3.323141	0.000000
Au	23.023397	3.323141	0.000000
Au	-12.950661	25.754343	0.000000
Au	8.633774	13.292564	0.000000
Au	24.462359	0.830785	0.000000
Au	-1.438962	20.769632	0.000000
Au	0.000000	13.292564	0.000000
Au	1.438962	35.723766	0.000000
Au	10.072736	10.800209	0.000000
Au	-7.194811	20.769632	0.000000
Au	2.877925	33.231411	0.000000
Au	0.000000	18.277275	0.000000
Au	11.511698	8.307853	0.000000
Au	4.316887	30.739056	0.000000
Au	4.316887	0.830785	0.000000
Au	1.438962	10.800209	0.000000
Au	5.755849	28.246698	0.000000
Au	12.950661	5.815497	0.000000
Au	1.438962	15.784920	0.000000
Au	7.194811	25.754343	0.000000
Au	-4.316887	10.800209	0.000000
Au	14.389623	3.323141	0.000000
Au	8.633774	23.261988	0.000000
Au	-5.755849	18.277275	0.000000
Au	2.877925	13.292564	0.000000
Au	10.072736	20.769632	0.000000
Au	15.828585	0.830785	0.000000
Au	2.877925	8.307853	0.000000
Au	11.511698	18.277275	0.000000
Au	-7.194811	15.784920	0.000000
Au	-7.194811	35.723766	0.000000
Au	12.950661	15.784920	0.000000
Au	4.316887	10.800209	0.000000

Au	-18.706511	35.723766	0.000000
Au	14.389623	13.292564	0.000000
Au	-5.755849	33.231411	0.000000
Au	4.316887	5.815497	0.000000
Au	15.828585	10.800209	0.000000
Au	5.755849	8.307853	0.000000
Au	-4.316887	30.739056	0.000000
Au	17.267548	8.307853	0.000000
Au	-4.316887	15.784920	0.000000
Au	-11.511698	23.261988	0.000000
Au	18.706511	5.815497	0.000000
Au	-2.877925	28.246698	0.000000
Au	7.194811	5.815497	0.000000
Au	20.145472	3.323141	0.000000
Au	5.755849	3.323141	0.000000
Au	-1.438962	25.754343	0.000000
Au	21.584435	0.830785	0.000000
Au	1.438962	0.830785	0.000000
Au	8.633774	3.323141	0.000000
Au	-1.438962	35.723766	0.000000
Au	0.000000	23.261988	0.000000
Au	-2.877925	13.292564	0.000000
Au	0.000000	33.231411	0.000000
Au	7.194811	0.830785	0.000000
Au	1.438962	20.769632	0.000000
Au	1.438962	30.739056	0.000000
Au	10.072736	0.830785	0.000000
Au	-17.267548	33.231411	0.000000
Au	2.877925	28.246698	0.000000
Au	2.877925	18.277275	0.000000
Au	-1.438962	5.815497	0.000000
Au	4.316887	25.754343	0.000000
Au	-12.950661	35.723766	0.000000
Au	4.316887	15.784920	0.000000

Au	5.755849	23.261988	0.000000
Au	-15.828585	35.723766	0.000000
Au	-1.438962	10.800209	0.000000
Au	7.194811	20.769632	0.000000
Au	5.755849	13.292564	0.000000
Au	-11.511698	33.231411	0.000000
Au	8.633774	18.277275	0.000000
Au	-10.072736	20.769632	0.000000
Au	7.194811	10.800209	0.000000
Au	10.072736	15.784920	0.000000
Au	-14.389623	33.231411	0.000000
Au	-10.072736	30.739056	0.000000
Au	11.511698	13.292564	0.000000
Au	8.633774	8.307853	0.000000
Au	-15.828585	30.739056	0.000000
Au	12.950661	10.800209	0.000000
Au	0.000000	8.307853	0.000000
Au	10.072736	5.815497	0.000000
Au	14.389623	8.307853	0.000000
Au	-8.633774	28.246698	0.000000
Au	-12.950661	30.739056	0.000000
Au	15.828585	5.815497	0.000000
Au	11.511698	3.323141	0.000000
Au	-5.755849	13.292564	0.000000
Au	17.267548	3.323141	0.000000
Au	-7.194811	25.754343	0.000000
Au	12.950661	0.830785	0.000000
Au	18.706511	0.830785	0.000000
Au	1.438962	19.108061	-2.349816
Au	2.877925	36.554550	-2.349816
Au	1.438962	9.138637	-2.349816
Au	4.316887	34.062195	-2.349816
Au	2.877925	16.615706	-2.349816
Au	5.755849	31.569840	-2.349816

Au	1.438962	4.153926	-2.349816
Au	7.194811	29.077484	-2.349816
Au	4.316887	14.123350	-2.349816
Au	8.633774	26.585129	-2.349816
Au	2.877925	6.646282	-2.349816
Au	10.072736	24.092773	-2.349816
Au	5.755849	11.630994	-2.349816
Au	11.511698	21.600416	-2.349816
Au	0.000000	1.661571	-2.349816
Au	12.950661	19.108061	-2.349816
Au	7.194811	9.138637	-2.349816
Au	14.389623	16.615706	-2.349816
Au	4.316887	4.153926	-2.349816
Au	15.828585	14.123350	-2.349816
Au	8.633774	6.646282	-2.349816
Au	17.267548	11.630994	-2.349816
Au	2.877925	1.661571	-2.349816
Au	18.706511	9.138637	-2.349816
Au	10.072736	4.153926	-2.349816
Au	20.145472	6.646282	-2.349816
Au	5.755849	1.661571	-2.349816
Au	21.584435	4.153926	-2.349816
Au	11.511698	1.661571	-2.349816
Au	23.023397	1.661571	-2.349816
Au	-10.072736	19.108061	-2.349816
Au	0.000000	36.554550	-2.349816
Au	-11.511698	36.554550	-2.349816
Au	1.438962	34.062195	-2.349816
Au	-17.267548	36.554550	-2.349816
Au	2.877925	31.569840	-2.349816
Au	-10.072736	34.062195	-2.349816
Au	4.316887	29.077484	-2.349816
Au	-20.145472	36.554550	-2.349816
Au	5.755849	26.585129	-2.349816

Au	-8.633774	31.569840	-2.349816
Au	7.194811	24.092773	-2.349816
Au	-15.828585	34.062195	-2.349816
Au	8.633774	21.600416	-2.349816
Au	-7.194811	29.077484	-2.349816
Au	10.072736	19.108061	-2.349816
Au	-4.316887	9.138637	-2.349816
Au	11.511698	16.615706	-2.349816
Au	-5.755849	26.585129	-2.349816
Au	12.950661	14.123350	-2.349816
Au	-14.389623	31.569840	-2.349816
Au	14.389623	11.630994	-2.349816
Au	-4.316887	24.092773	-2.349816
Au	15.828585	9.138637	-2.349816
Au	-18.706511	34.062195	-2.349816
Au	17.267548	6.646282	-2.349816
Au	-2.877925	21.600416	-2.349816
Au	18.706511	4.153926	-2.349816
Au	-12.950661	29.077484	-2.349816
Au	20.145472	1.661571	-2.349816
Au	-1.438962	19.108061	-2.349816
Au	-2.877925	36.554550	-2.349816
Au	-8.633774	16.615706	-2.349816
Au	-1.438962	34.062195	-2.349816
Au	0.000000	16.615706	-2.349816
Au	0.000000	31.569840	-2.349816
Au	-11.511698	26.585129	-2.349816
Au	1.438962	29.077484	-2.349816
Au	1.438962	14.123350	-2.349816
Au	2.877925	26.585129	-2.349816
Au	-17.267548	31.569840	-2.349816
Au	4.316887	24.092773	-2.349816
Au	2.877925	11.630994	-2.349816
Au	5.755849	21.600416	-2.349816

Au	-10.072736	24.092773	-2.349816
Au	7.194811	19.108061	-2.349816
Au	4.316887	9.138637	-2.349816
Au	8.633774	16.615706	-2.349816
Au	-1.438962	4.153926	-2.349816
Au	10.072736	14.123350	-2.349816
Au	5.755849	6.646282	-2.349816
Au	11.511698	11.630994	-2.349816
Au	-8.633774	21.600416	-2.349816
Au	12.950661	9.138637	-2.349816
Au	7.194811	4.153926	-2.349816
Au	14.389623	6.646282	-2.349816
Au	-15.828585	29.077484	-2.349816
Au	15.828585	4.153926	-2.349816
Au	8.633774	1.661571	-2.349816
Au	17.267548	1.661571	-2.349816
Au	-7.194811	19.108061	-2.349816
Au	-5.755849	36.554550	-2.349816
Au	-14.389623	36.554550	-2.349816
Au	-4.316887	34.062195	-2.349816
Au	-7.194811	14.123350	-2.349816
Au	-2.877925	31.569840	-2.349816
Au	-12.950661	34.062195	-2.349816
Au	-1.438962	29.077484	-2.349816
Au	-5.755849	16.615706	-2.349816
Au	0.000000	26.585129	-2.349816
Au	-11.511698	31.569840	-2.349816
Au	1.438962	24.092773	-2.349816
Au	-14.389623	26.585129	-2.349816
Au	2.877925	21.600416	-2.349816
Au	-10.072736	29.077484	-2.349816
Au	4.316887	19.108061	-2.349816
Au	-4.316887	14.123350	-2.349816
Au	5.755849	16.615706	-2.349816

Au	-8.633774	26.585129	-2.349816
Au	7.194811	14.123350	-2.349816
Au	-2.877925	6.646282	-2.349816
Au	8.633774	11.630994	-2.349816
Au	-7.194811	24.092773	-2.349816
Au	10.072736	9.138637	-2.349816
Au	-2.877925	11.630994	-2.349816
Au	11.511698	6.646282	-2.349816
Au	-5.755849	21.600416	-2.349816
Au	12.950661	4.153926	-2.349816
Au	-12.950661	24.092773	-2.349816
Au	14.389623	1.661571	-2.349816
Au	-4.316887	19.108061	-2.349816
Au	-8.633774	36.554550	-2.349816
Au	-1.438962	9.138637	-2.349816
Au	-7.194811	34.062195	-2.349816
Au	-2.877925	16.615706	-2.349816
Au	-5.755849	31.569840	-2.349816
Au	-5.755849	11.630994	-2.349816
Au	-4.316887	29.077484	-2.349816
Au	-1.438962	14.123350	-2.349816
Au	-2.877925	26.585129	-2.349816
Au	0.000000	6.646282	-2.349816
Au	-1.438962	24.092773	-2.349816
Au	0.000000	11.630994	-2.349816
Au	0.000000	21.600416	-2.349816
Au	-11.511698	21.600416	-2.349816
Au	2.877925	34.892983	-4.699631
Au	4.316887	32.400627	-4.699631
Au	5.755849	29.908270	-4.699631
Au	7.194811	27.415914	-4.699631
Au	8.633774	24.923557	-4.699631
Au	10.072736	22.431202	-4.699631
Au	11.511698	19.938847	-4.699631

Au	12.950661	17.446491	-4.699631
Au	14.389623	14.954135	-4.699631
Au	15.828585	12.461779	-4.699631
Au	17.267548	9.969423	-4.699631
Au	18.706511	7.477067	-4.699631
Au	20.145472	4.984712	-4.699631
Au	21.584435	2.492356	-4.699631
Au	23.023397	0.000000	-4.699631
Au	0.000000	34.892983	-4.699631
Au	1.438962	32.400627	-4.699631
Au	2.877925	29.908270	-4.699631
Au	4.316887	27.415914	-4.699631
Au	5.755849	24.923557	-4.699631
Au	7.194811	22.431202	-4.699631
Au	8.633774	19.938847	-4.699631
Au	10.072736	17.446491	-4.699631
Au	11.511698	14.954135	-4.699631
Au	12.950661	12.461779	-4.699631
Au	14.389623	9.969423	-4.699631
Au	15.828585	7.477067	-4.699631
Au	17.267548	4.984712	-4.699631
Au	18.706511	2.492356	-4.699631
Au	20.145472	0.000000	-4.699631
Au	-2.877925	34.892983	-4.699631
Au	-1.438962	32.400627	-4.699631
Au	0.000000	29.908270	-4.699631
Au	1.438962	27.415914	-4.699631
Au	2.877925	24.923557	-4.699631
Au	4.316887	22.431202	-4.699631
Au	5.755849	19.938847	-4.699631
Au	7.194811	17.446491	-4.699631
Au	8.633774	14.954135	-4.699631
Au	10.072736	12.461779	-4.699631
Au	11.511698	9.969423	-4.699631

Au	12.950661	7.477067	-4.699631
Au	14.389623	4.984712	-4.699631
Au	15.828585	2.492356	-4.699631
Au	17.267548	0.000000	-4.699631
Au	-5.755849	34.892983	-4.699631
Au	-4.316887	32.400627	-4.699631
Au	-2.877925	29.908270	-4.699631
Au	-1.438962	27.415914	-4.699631
Au	0.000000	24.923557	-4.699631
Au	1.438962	22.431202	-4.699631
Au	2.877925	19.938847	-4.699631
Au	4.316887	17.446491	-4.699631
Au	5.755849	14.954135	-4.699631
Au	7.194811	12.461779	-4.699631
Au	8.633774	9.969423	-4.699631
Au	10.072736	7.477067	-4.699631
Au	11.511698	4.984712	-4.699631
Au	12.950661	2.492356	-4.699631
Au	14.389623	0.000000	-4.699631
Au	-8.633774	34.892983	-4.699631
Au	-7.194811	32.400627	-4.699631
Au	-5.755849	29.908270	-4.699631
Au	-4.316887	27.415914	-4.699631
Au	-2.877925	24.923557	-4.699631
Au	-1.438962	22.431202	-4.699631
Au	0.000000	19.938847	-4.699631
Au	1.438962	17.446491	-4.699631
Au	2.877925	14.954135	-4.699631
Au	4.316887	12.461779	-4.699631
Au	5.755849	9.969423	-4.699631
Au	7.194811	7.477067	-4.699631
Au	8.633774	4.984712	-4.699631
Au	10.072736	2.492356	-4.699631
Au	11.511698	0.000000	-4.699631

Au	-11.511698	34.892983	-4.699631
Au	-10.072736	32.400627	-4.699631
Au	-8.633774	29.908270	-4.699631
Au	-7.194811	27.415914	-4.699631
Au	-5.755849	24.923557	-4.699631
Au	-4.316887	22.431202	-4.699631
Au	-2.877925	19.938847	-4.699631
Au	-1.438962	17.446491	-4.699631
Au	0.000000	14.954135	-4.699631
Au	1.438962	12.461779	-4.699631
Au	2.877925	9.969423	-4.699631
Au	4.316887	7.477067	-4.699631
Au	5.755849	4.984712	-4.699631
Au	7.194811	2.492356	-4.699631
Au	8.633774	0.000000	-4.699631
Au	-14.389623	34.892983	-4.699631
Au	-12.950661	32.400627	-4.699631
Au	-11.511698	29.908270	-4.699631
Au	-10.072736	27.415914	-4.699631
Au	-8.633774	24.923557	-4.699631
Au	-7.194811	22.431202	-4.699631
Au	-5.755849	19.938847	-4.699631
Au	-4.316887	17.446491	-4.699631
Au	-2.877925	14.954135	-4.699631
Au	-1.438962	12.461779	-4.699631
Au	0.000000	9.969423	-4.699631
Au	1.438962	7.477067	-4.699631
Au	2.877925	4.984712	-4.699631
Au	4.316887	2.492356	-4.699631
Au	5.755849	0.000000	-4.699631
Au	-17.267548	34.892983	-4.699631
Au	-15.828585	32.400627	-4.699631
Au	-14.389623	29.908270	-4.699631
Au	-12.950661	27.415914	-4.699631

Au	-11.511698	24.923557	-4.699631
Au	-10.072736	22.431202	-4.699631
Au	-8.633774	19.938847	-4.699631
Au	-7.194811	17.446491	-4.699631
Au	-5.755849	14.954135	-4.699631
Au	-4.316887	12.461779	-4.699631
Au	-2.877925	9.969423	-4.699631
Au	-1.438962	7.477067	-4.699631
Au	0.000000	4.984712	-4.699631
Au	1.438962	2.492356	-4.699631
Au	2.877925	0.000000	-4.699631
Au	-20.145472	34.892983	-4.699631
Au	-18.706511	32.400627	-4.699631
Au	-17.267548	29.908270	-4.699631
Au	-15.828585	27.415914	-4.699631
Au	-14.389623	24.923557	-4.699631
Au	-12.950661	22.431202	-4.699631
Au	-11.511698	19.938847	-4.699631
Au	-10.072736	17.446491	-4.699631
Au	-8.633774	14.954135	-4.699631
Au	-7.194811	12.461779	-4.699631
Au	-5.755849	9.969423	-4.699631
Au	-4.316887	7.477067	-4.699631
Au	-2.877925	4.984712	-4.699631
Au	-1.438962	2.492356	-4.699631
Au	0.000000	0.000000	-4.699631

Azobenzene dimer of 5-(4-Nitrophenyl)-10,15,20-tris(3,5-di-t-butylphenyl)porphyrin platinum(II) (**2**) on Au(111) Surface
(homochiral, parallel orientation of macrocyclic saddlings, NOT observed experimentally)

C	10.180149	11.110578	2.349078
C	8.943258	11.832974	2.687931
N	7.896188	10.899753	2.905392
C	8.479462	9.598411	2.772024
C	4.370369	7.471041	4.028418
C	5.736470	7.155639	4.001343
C	6.413903	8.271968	3.346055
N	5.475297	9.281965	3.008795
C	4.217628	8.746375	3.347446
C	7.812958	8.340527	3.022045
C	1.875344	12.780365	2.211432
C	1.562073	11.369139	2.256104
C	2.785050	10.704309	2.703026
N	3.832093	11.656665	2.880177
C	3.248028	12.941765	2.646331
C	2.947461	9.286095	2.923490
C	7.350912	15.216958	3.666571
C	5.972217	15.475756	3.718915
C	5.299095	14.309900	3.173443
N	6.252696	13.304745	2.833514
C	7.518739	13.889641	3.089436
C	8.803844	13.278387	2.804626
C	3.882123	14.218049	2.956417
C	1.849967	8.307895	2.730764
C	8.557339	7.047948	2.946828
C	10.022702	14.147705	2.820520
C	3.058237	15.450743	2.926667
C	9.950328	6.909945	3.253020
C	10.607467	5.649760	3.131990
C	9.829631	4.462921	2.947453
C	8.405950	4.555532	2.805321

C	7.819400	5.848391	2.622952
C	2.164045	6.923024	2.441882
C	1.171535	5.883146	2.531358
C	-0.214168	6.262808	2.690096
C	-0.564663	7.638812	2.847794
C	0.460620	8.635478	2.898215
C	3.592135	16.739923	2.549385
C	2.749629	17.904425	2.423571
C	0.766767	16.453477	2.835160
C	1.628655	15.394685	3.184414
C	11.295970	13.601057	3.193454
C	12.504379	14.345017	3.091465
C	12.438480	15.753438	2.868512
C	11.158173	16.383396	2.753927
C	9.988019	15.577186	2.518131
C	1.610906	4.613169	3.293686
C	-1.928709	8.055080	3.416345
C	3.113301	4.285677	3.216838
C	-3.108515	7.140899	2.991062
C	12.093715	5.589476	3.509236
C	12.843921	6.877053	3.054301
C	7.541984	3.393436	3.323236
C	8.158700	1.999500	3.014207
C	13.806961	13.652497	3.502024
C	13.764719	12.162257	3.079029
C	11.017516	17.787417	3.355984
C	12.112552	18.805811	2.953180
C	1.283120	5.037998	4.759529
C	-1.752409	7.922817	4.951713
C	12.145623	5.551315	5.055970
C	7.682216	3.577838	4.865353
C	15.050968	14.408751	2.966373
C	9.610087	18.376788	3.195949
C	0.799335	3.333139	3.010613

C	-2.221286	9.549821	3.136509
C	12.748756	4.286963	2.998507
C	6.018011	3.506871	3.021771
C	13.864333	13.694921	5.046752
C	11.203770	17.495714	4.871609
C	1.297492	17.771349	2.514534
N	0.347328	18.797416	2.320204
C	9.882006	9.722772	2.446493
H	11.222666	11.540875	2.242954
H	3.494912	6.877920	4.457865
H	6.264336	6.222194	4.394518
Pt	5.866625	11.279985	2.812891
H	1.103417	13.603045	2.090218
H	0.493880	10.985365	2.273333
H	8.220667	15.809297	4.107958
H	5.408948	16.369734	4.161358
H	10.588657	8.861208	2.218860
H	10.546285	7.780807	3.673280
H	10.330703	3.435786	3.030676
H	6.703221	5.965249	2.465152
H	3.256711	6.614726	2.365565
H	-0.992797	5.457829	2.912480
H	0.179452	9.643884	3.335143
H	4.713169	16.845163	2.347796
H	3.190153	18.961909	2.343461
H	-0.378757	16.347024	2.940462
H	1.200160	14.452964	3.661479
H	11.345194	12.570353	3.659143
H	13.375829	16.383456	3.038770
H	8.987565	16.102458	2.386119
H	3.267634	3.318805	3.815381
H	3.457955	4.073713	2.132455
H	3.786104	5.098283	3.656252
H	-3.875361	7.718870	2.352069

H	-2.771993	6.230274	2.374546
H	-3.647023	6.761589	3.937093
H	13.852503	6.626915	2.555778
H	12.233637	7.479222	2.286472
H	13.040091	7.551603	3.966330
H	7.345361	1.245213	2.699746
H	8.950532	2.042541	2.179164
H	8.675326	1.599168	3.960960
H	14.830777	11.736996	2.985170
H	13.185136	11.546132	3.855480
H	13.245764	12.043201	2.056332
H	11.684150	19.587766	2.222678
H	12.477340	19.359101	3.892122
H	13.025468	18.313510	2.464110
H	1.850749	4.356562	5.493909
H	0.151121	4.921273	4.916679
H	1.536247	6.147386	4.931632
H	-2.699414	8.334506	5.458433
H	-0.832138	8.530666	5.276740
H	-1.599312	6.817624	5.231452
H	13.247895	5.488666	5.379926
H	11.574273	4.628048	5.434618
H	11.665243	6.504468	5.481298
H	7.117089	2.718312	5.376824
H	7.241652	4.586314	5.196640
H	8.796588	3.554384	5.143133
H	15.537659	15.007410	3.820768
H	14.768944	15.145098	2.127937
H	15.826959	13.674972	2.534103
H	9.620722	19.443078	3.625281
H	8.834741	17.753188	3.767869
H	9.298848	18.441137	2.077833
H	1.342853	2.661126	2.249824
H	0.715583	2.752421	4.001088

H	-0.265113	3.561464	2.634563
H	-1.821918	9.863616	2.095875
H	-3.356488	9.734194	3.164281
H	-1.725055	10.204537	3.937846
H	13.865349	4.451290	2.771507
H	12.636279	3.455432	3.784269
H	12.243159	3.923819	2.024351
H	5.615236	2.563878	2.498417
H	5.450315	3.619884	4.012413
H	5.719585	4.407442	2.380256
H	12.940689	13.168709	5.485361
H	14.826873	13.164681	5.389859
H	13.883127	14.791267	5.392330
H	12.289062	17.166367	5.054004
H	10.967343	18.458034	5.456102
H	10.504503	16.641401	5.195707
C	-9.000461	27.541333	2.338294
C	-7.749358	26.853644	2.671164
N	-6.732349	27.825962	2.894637
C	-7.347241	29.109878	2.751698
C	-3.289658	31.317025	4.022728
C	-4.660335	31.608907	3.990624
C	-5.322883	30.480074	3.337570
N	-4.366044	29.479553	3.017266
C	-3.117905	30.032712	3.365051
C	-6.720836	30.394707	2.990239
C	-0.660453	26.077945	2.208638
C	-0.398069	27.498942	2.278077
C	-1.646008	28.100847	2.724091
N	-2.662267	27.115347	2.893891
C	-2.027498	25.860310	2.665027
C	-1.833248	29.497512	2.991160
C	-6.087650	23.488196	3.636687
C	-4.700886	23.264776	3.709353

C	-4.049369	24.451856	3.181105
N	-5.025555	25.436750	2.847114
C	-6.280641	24.819600	3.074612
C	-7.577789	25.408813	2.795358
C	-2.630419	24.567681	2.953140
C	-0.753471	30.477047	2.734654
C	-7.501520	31.677268	2.928095
C	-8.771733	24.506040	2.755909
C	-1.775043	23.354312	2.910536
C	-8.915314	31.775272	3.181520
C	-9.589386	33.029139	3.101435
C	-8.837174	34.233936	2.968232
C	-7.413021	34.187019	2.848923
C	-6.786142	32.913420	2.687907
C	-1.154898	31.781358	2.230595
C	-0.228899	32.924111	2.260350
C	1.177660	32.606045	2.442232
C	1.612506	31.282586	2.756073
C	0.641767	30.243786	2.969853
C	-2.281854	22.054424	2.540382
C	-1.419208	20.902461	2.399401
C	0.537049	22.393735	2.831747
C	-0.342751	23.446044	3.156941
C	-10.068572	25.016985	3.096460
C	-11.262674	24.257166	2.959039
C	-11.162886	22.853128	2.684280
C	-9.866059	22.243234	2.614844
C	-8.697978	23.080393	2.420419
C	-0.703479	34.088519	3.172638
C	2.968547	31.127247	3.460774
C	-2.205116	34.421434	3.114034
C	3.913355	32.321804	3.250765
C	-11.059385	33.108102	3.523360
C	-11.888560	31.883224	3.040799

C	-6.608876	35.415177	3.311599
C	-7.288641	36.768676	2.941645
C	-12.540110	24.877542	3.534505
C	-12.554533	26.412063	3.339850
C	-9.725534	20.870041	3.293472
C	-10.806114	19.814980	2.945413
C	-0.354464	33.511487	4.584529
C	2.593150	31.144086	4.966421
C	-11.021930	33.076169	5.071406
C	-6.730532	35.303061	4.859698
C	-13.858677	24.269143	3.004552
C	-8.320239	20.263145	3.182019
C	0.083757	35.402436	3.014761
C	3.705135	29.809584	3.146870
C	-11.684741	34.460125	3.109644
C	-5.084971	35.348816	3.003294
C	-12.464537	24.585962	5.053762
C	-9.931838	21.257689	4.785894
C	0.034215	21.064588	2.516189
N	1.006693	20.059274	2.354322
C	-8.743666	28.938239	2.392669
H	-10.025938	27.083598	2.175166
H	-2.424112	31.944603	4.419605
H	-5.203559	32.529840	4.392967
Pt	-4.702781	27.469431	2.820134
H	0.147654	25.289271	2.113968
H	0.641889	27.957493	2.295999
H	-6.940709	22.867633	4.068309
H	-4.130447	22.377441	4.151320
H	-9.504648	29.760401	2.200962
H	-9.507980	30.888362	3.572063
H	-9.376398	35.232679	3.089242
H	-5.667205	32.837753	2.513594
H	-2.265217	32.028378	2.283536

H	1.928890	33.457377	2.545548
H	0.971733	29.308031	3.528205
H	-3.402499	21.937323	2.342551
H	-1.849114	19.835857	2.354322
H	1.681514	22.519051	2.933596
H	0.073780	24.391855	3.633413
H	-10.137630	26.029283	3.594987
H	-12.068274	22.182733	2.872106
H	-7.680425	22.576103	2.347438
H	-2.358242	35.379305	3.727134
H	-2.543622	34.670692	2.040105
H	-2.888813	33.606465	3.528452
H	4.922072	32.054194	3.735194
H	4.087829	32.554850	2.133289
H	3.495601	33.248543	3.781830
H	-12.822724	32.218624	2.454576
H	-11.281253	31.201501	2.340991
H	-12.225399	31.262552	3.951264
H	-6.577483	37.459963	2.353995
H	-8.234267	36.634240	2.301316
H	-7.597782	37.308170	3.911748
H	-13.633509	26.786020	3.474885
H	-11.887388	26.933855	4.113464
H	-12.208420	26.699321	2.275766
H	-10.359384	19.002535	2.259727
H	-11.163407	19.309146	3.913706
H	-11.728081	20.250434	2.420092
H	-1.021928	34.001142	5.385705
H	0.743582	33.766543	4.798110
H	-0.451125	32.366115	4.605250
H	3.558544	31.232845	5.585346
H	2.024623	30.188285	5.251914
H	1.911389	32.048607	5.167630
H	-12.089868	33.226442	5.473054

H	-10.334173	33.919088	5.442996
H	-10.603561	32.065839	5.422626
H	-6.153430	36.182579	5.321606
H	-6.287535	34.305715	5.222093
H	-7.841104	35.354171	5.149280
H	-14.598900	24.195756	3.883409
H	-13.727594	23.219727	2.549208
H	-14.329050	24.957728	2.211245
H	-8.329116	19.269541	3.760574
H	-7.527941	20.948975	3.650056
H	-8.033817	20.036649	2.083093
H	-0.385626	36.082778	2.208272
H	0.029350	35.951194	4.024646
H	1.191564	35.229305	2.760516
H	4.399848	29.921339	2.224022
H	4.357853	29.545987	4.059713
H	2.973891	28.944346	2.937957
H	-12.826910	34.358018	3.012723
H	-11.440770	35.253450	3.903620
H	-11.267055	34.825787	2.101442
H	-4.710128	36.343529	2.557884
H	-4.508682	35.162237	3.977597
H	-4.784742	34.510537	2.282514
H	-11.444328	24.926298	5.461320
H	-13.316029	25.154290	5.578448
H	-12.590079	23.454822	5.226470
H	-11.029465	21.561434	4.937127
H	-9.667421	20.348400	5.439102
H	-9.267620	22.159528	5.052450
Au	-2.877925	8.307853	0.000000
Au	-11.511698	28.246698	0.000000
Au	4.316887	35.723766	0.000000
Au	-4.316887	35.723766	0.000000
Au	-10.072736	35.723766	0.000000

Au	5.755849	33.231411	0.000000
Au	-5.755849	23.261988	0.000000
Au	-2.877925	33.231411	0.000000
Au	7.194811	30.739056	0.000000
Au	1.438962	5.815497	0.000000
Au	-8.633774	33.231411	0.000000
Au	8.633774	28.246698	0.000000
Au	-1.438962	30.739056	0.000000
Au	-14.389623	28.246698	0.000000
Au	10.072736	25.754343	0.000000
Au	-4.316887	20.769632	0.000000
Au	0.000000	28.246698	0.000000
Au	11.511698	23.261988	0.000000
Au	-7.194811	30.739056	0.000000
Au	-10.072736	25.754343	0.000000
Au	12.950661	20.769632	0.000000
Au	1.438962	25.754343	0.000000
Au	-8.633774	18.277275	0.000000
Au	14.389623	18.277275	0.000000
Au	-5.755849	28.246698	0.000000
Au	2.877925	23.261988	0.000000
Au	15.828585	15.784920	0.000000
Au	-2.877925	18.277275	0.000000
Au	2.877925	3.323141	0.000000
Au	17.267548	13.292564	0.000000
Au	4.316887	20.769632	0.000000
Au	-4.316887	25.754343	0.000000
Au	18.706511	10.800209	0.000000
Au	-8.633774	23.261988	0.000000
Au	5.755849	18.277275	0.000000
Au	20.145472	8.307853	0.000000
Au	-1.438962	15.784920	0.000000
Au	-2.877925	23.261988	0.000000
Au	21.584435	5.815497	0.000000

Au	7.194811	15.784920	0.000000
Au	0.000000	3.323141	0.000000
Au	23.023397	3.323141	0.000000
Au	-12.950661	25.754343	0.000000
Au	8.633774	13.292564	0.000000
Au	24.462359	0.830785	0.000000
Au	-1.438962	20.769632	0.000000
Au	0.000000	13.292564	0.000000
Au	1.438962	35.723766	0.000000
Au	10.072736	10.800209	0.000000
Au	-7.194811	20.769632	0.000000
Au	2.877925	33.231411	0.000000
Au	0.000000	18.277275	0.000000
Au	11.511698	8.307853	0.000000
Au	4.316887	30.739056	0.000000
Au	4.316887	0.830785	0.000000
Au	1.438962	10.800209	0.000000
Au	5.755849	28.246698	0.000000
Au	12.950661	5.815497	0.000000
Au	1.438962	15.784920	0.000000
Au	7.194811	25.754343	0.000000
Au	-4.316887	10.800209	0.000000
Au	14.389623	3.323141	0.000000
Au	8.633774	23.261988	0.000000
Au	-5.755849	18.277275	0.000000
Au	2.877925	13.292564	0.000000
Au	10.072736	20.769632	0.000000
Au	15.828585	0.830785	0.000000
Au	2.877925	8.307853	0.000000
Au	11.511698	18.277275	0.000000
Au	-7.194811	15.784920	0.000000
Au	-7.194811	35.723766	0.000000
Au	12.950661	15.784920	0.000000
Au	4.316887	10.800209	0.000000

Au	-18.706511	35.723766	0.000000
Au	14.389623	13.292564	0.000000
Au	-5.755849	33.231411	0.000000
Au	4.316887	5.815497	0.000000
Au	15.828585	10.800209	0.000000
Au	5.755849	8.307853	0.000000
Au	-4.316887	30.739056	0.000000
Au	17.267548	8.307853	0.000000
Au	-4.316887	15.784920	0.000000
Au	-11.511698	23.261988	0.000000
Au	18.706511	5.815497	0.000000
Au	-2.877925	28.246698	0.000000
Au	7.194811	5.815497	0.000000
Au	20.145472	3.323141	0.000000
Au	5.755849	3.323141	0.000000
Au	-1.438962	25.754343	0.000000
Au	21.584435	0.830785	0.000000
Au	1.438962	0.830785	0.000000
Au	8.633774	3.323141	0.000000
Au	-1.438962	35.723766	0.000000
Au	0.000000	23.261988	0.000000
Au	-2.877925	13.292564	0.000000
Au	0.000000	33.231411	0.000000
Au	7.194811	0.830785	0.000000
Au	1.438962	20.769632	0.000000
Au	1.438962	30.739056	0.000000
Au	10.072736	0.830785	0.000000
Au	-17.267548	33.231411	0.000000
Au	2.877925	28.246698	0.000000
Au	2.877925	18.277275	0.000000
Au	-1.438962	5.815497	0.000000
Au	4.316887	25.754343	0.000000
Au	-12.950661	35.723766	0.000000
Au	4.316887	15.784920	0.000000

Au	5.755849	23.261988	0.000000
Au	-15.828585	35.723766	0.000000
Au	-1.438962	10.800209	0.000000
Au	7.194811	20.769632	0.000000
Au	5.755849	13.292564	0.000000
Au	-11.511698	33.231411	0.000000
Au	8.633774	18.277275	0.000000
Au	-10.072736	20.769632	0.000000
Au	7.194811	10.800209	0.000000
Au	10.072736	15.784920	0.000000
Au	-14.389623	33.231411	0.000000
Au	-10.072736	30.739056	0.000000
Au	11.511698	13.292564	0.000000
Au	8.633774	8.307853	0.000000
Au	-15.828585	30.739056	0.000000
Au	12.950661	10.800209	0.000000
Au	0.000000	8.307853	0.000000
Au	10.072736	5.815497	0.000000
Au	14.389623	8.307853	0.000000
Au	-8.633774	28.246698	0.000000
Au	-12.950661	30.739056	0.000000
Au	15.828585	5.815497	0.000000
Au	11.511698	3.323141	0.000000
Au	-5.755849	13.292564	0.000000
Au	17.267548	3.323141	0.000000
Au	-7.194811	25.754343	0.000000
Au	12.950661	0.830785	0.000000
Au	18.706511	0.830785	0.000000
Au	1.438962	19.108061	-2.349816
Au	2.877925	36.554550	-2.349816
Au	1.438962	9.138637	-2.349816
Au	4.316887	34.062195	-2.349816
Au	2.877925	16.615706	-2.349816
Au	5.755849	31.569840	-2.349816

Au	1.438962	4.153926	-2.349816
Au	7.194811	29.077484	-2.349816
Au	4.316887	14.123350	-2.349816
Au	8.633774	26.585129	-2.349816
Au	2.877925	6.646282	-2.349816
Au	10.072736	24.092773	-2.349816
Au	5.755849	11.630994	-2.349816
Au	11.511698	21.600416	-2.349816
Au	0.000000	1.661571	-2.349816
Au	12.950661	19.108061	-2.349816
Au	7.194811	9.138637	-2.349816
Au	14.389623	16.615706	-2.349816
Au	4.316887	4.153926	-2.349816
Au	15.828585	14.123350	-2.349816
Au	8.633774	6.646282	-2.349816
Au	17.267548	11.630994	-2.349816
Au	2.877925	1.661571	-2.349816
Au	18.706511	9.138637	-2.349816
Au	10.072736	4.153926	-2.349816
Au	20.145472	6.646282	-2.349816
Au	5.755849	1.661571	-2.349816
Au	21.584435	4.153926	-2.349816
Au	11.511698	1.661571	-2.349816
Au	23.023397	1.661571	-2.349816
Au	-10.072736	19.108061	-2.349816
Au	0.000000	36.554550	-2.349816
Au	-11.511698	36.554550	-2.349816
Au	1.438962	34.062195	-2.349816
Au	-17.267548	36.554550	-2.349816
Au	2.877925	31.569840	-2.349816
Au	-10.072736	34.062195	-2.349816
Au	4.316887	29.077484	-2.349816
Au	-20.145472	36.554550	-2.349816
Au	5.755849	26.585129	-2.349816

Au	-8.633774	31.569840	-2.349816
Au	7.194811	24.092773	-2.349816
Au	-15.828585	34.062195	-2.349816
Au	8.633774	21.600416	-2.349816
Au	-7.194811	29.077484	-2.349816
Au	10.072736	19.108061	-2.349816
Au	-4.316887	9.138637	-2.349816
Au	11.511698	16.615706	-2.349816
Au	-5.755849	26.585129	-2.349816
Au	12.950661	14.123350	-2.349816
Au	-14.389623	31.569840	-2.349816
Au	14.389623	11.630994	-2.349816
Au	-4.316887	24.092773	-2.349816
Au	15.828585	9.138637	-2.349816
Au	-18.706511	34.062195	-2.349816
Au	17.267548	6.646282	-2.349816
Au	-2.877925	21.600416	-2.349816
Au	18.706511	4.153926	-2.349816
Au	-12.950661	29.077484	-2.349816
Au	20.145472	1.661571	-2.349816
Au	-1.438962	19.108061	-2.349816
Au	-2.877925	36.554550	-2.349816
Au	-8.633774	16.615706	-2.349816
Au	-1.438962	34.062195	-2.349816
Au	0.000000	16.615706	-2.349816
Au	0.000000	31.569840	-2.349816
Au	-11.511698	26.585129	-2.349816
Au	1.438962	29.077484	-2.349816
Au	1.438962	14.123350	-2.349816
Au	2.877925	26.585129	-2.349816
Au	-17.267548	31.569840	-2.349816
Au	4.316887	24.092773	-2.349816
Au	2.877925	11.630994	-2.349816
Au	5.755849	21.600416	-2.349816

Au	-10.072736	24.092773	-2.349816
Au	7.194811	19.108061	-2.349816
Au	4.316887	9.138637	-2.349816
Au	8.633774	16.615706	-2.349816
Au	-1.438962	4.153926	-2.349816
Au	10.072736	14.123350	-2.349816
Au	5.755849	6.646282	-2.349816
Au	11.511698	11.630994	-2.349816
Au	-8.633774	21.600416	-2.349816
Au	12.950661	9.138637	-2.349816
Au	7.194811	4.153926	-2.349816
Au	14.389623	6.646282	-2.349816
Au	-15.828585	29.077484	-2.349816
Au	15.828585	4.153926	-2.349816
Au	8.633774	1.661571	-2.349816
Au	17.267548	1.661571	-2.349816
Au	-7.194811	19.108061	-2.349816
Au	-5.755849	36.554550	-2.349816
Au	-14.389623	36.554550	-2.349816
Au	-4.316887	34.062195	-2.349816
Au	-7.194811	14.123350	-2.349816
Au	-2.877925	31.569840	-2.349816
Au	-12.950661	34.062195	-2.349816
Au	-1.438962	29.077484	-2.349816
Au	-5.755849	16.615706	-2.349816
Au	0.000000	26.585129	-2.349816
Au	-11.511698	31.569840	-2.349816
Au	1.438962	24.092773	-2.349816
Au	-14.389623	26.585129	-2.349816
Au	2.877925	21.600416	-2.349816
Au	-10.072736	29.077484	-2.349816
Au	4.316887	19.108061	-2.349816
Au	-4.316887	14.123350	-2.349816
Au	5.755849	16.615706	-2.349816

Au	-8.633774	26.585129	-2.349816
Au	7.194811	14.123350	-2.349816
Au	-2.877925	6.646282	-2.349816
Au	8.633774	11.630994	-2.349816
Au	-7.194811	24.092773	-2.349816
Au	10.072736	9.138637	-2.349816
Au	-2.877925	11.630994	-2.349816
Au	11.511698	6.646282	-2.349816
Au	-5.755849	21.600416	-2.349816
Au	12.950661	4.153926	-2.349816
Au	-12.950661	24.092773	-2.349816
Au	14.389623	1.661571	-2.349816
Au	-4.316887	19.108061	-2.349816
Au	-8.633774	36.554550	-2.349816
Au	-1.438962	9.138637	-2.349816
Au	-7.194811	34.062195	-2.349816
Au	-2.877925	16.615706	-2.349816
Au	-5.755849	31.569840	-2.349816
Au	-5.755849	11.630994	-2.349816
Au	-4.316887	29.077484	-2.349816
Au	-1.438962	14.123350	-2.349816
Au	-2.877925	26.585129	-2.349816
Au	0.000000	6.646282	-2.349816
Au	-1.438962	24.092773	-2.349816
Au	0.000000	11.630994	-2.349816
Au	0.000000	21.600416	-2.349816
Au	-11.511698	21.600416	-2.349816
Au	2.877925	34.892983	-4.699631
Au	4.316887	32.400627	-4.699631
Au	5.755849	29.908270	-4.699631
Au	7.194811	27.415914	-4.699631
Au	8.633774	24.923557	-4.699631
Au	10.072736	22.431202	-4.699631
Au	11.511698	19.938847	-4.699631

Au	12.950661	17.446491	-4.699631
Au	14.389623	14.954135	-4.699631
Au	15.828585	12.461779	-4.699631
Au	17.267548	9.969423	-4.699631
Au	18.706511	7.477067	-4.699631
Au	20.145472	4.984712	-4.699631
Au	21.584435	2.492356	-4.699631
Au	23.023397	0.000000	-4.699631
Au	0.000000	34.892983	-4.699631
Au	1.438962	32.400627	-4.699631
Au	2.877925	29.908270	-4.699631
Au	4.316887	27.415914	-4.699631
Au	5.755849	24.923557	-4.699631
Au	7.194811	22.431202	-4.699631
Au	8.633774	19.938847	-4.699631
Au	10.072736	17.446491	-4.699631
Au	11.511698	14.954135	-4.699631
Au	12.950661	12.461779	-4.699631
Au	14.389623	9.969423	-4.699631
Au	15.828585	7.477067	-4.699631
Au	17.267548	4.984712	-4.699631
Au	18.706511	2.492356	-4.699631
Au	20.145472	0.000000	-4.699631
Au	-2.877925	34.892983	-4.699631
Au	-1.438962	32.400627	-4.699631
Au	0.000000	29.908270	-4.699631
Au	1.438962	27.415914	-4.699631
Au	2.877925	24.923557	-4.699631
Au	4.316887	22.431202	-4.699631
Au	5.755849	19.938847	-4.699631
Au	7.194811	17.446491	-4.699631
Au	8.633774	14.954135	-4.699631
Au	10.072736	12.461779	-4.699631
Au	11.511698	9.969423	-4.699631

Au	12.950661	7.477067	-4.699631
Au	14.389623	4.984712	-4.699631
Au	15.828585	2.492356	-4.699631
Au	17.267548	0.000000	-4.699631
Au	-5.755849	34.892983	-4.699631
Au	-4.316887	32.400627	-4.699631
Au	-2.877925	29.908270	-4.699631
Au	-1.438962	27.415914	-4.699631
Au	0.000000	24.923557	-4.699631
Au	1.438962	22.431202	-4.699631
Au	2.877925	19.938847	-4.699631
Au	4.316887	17.446491	-4.699631
Au	5.755849	14.954135	-4.699631
Au	7.194811	12.461779	-4.699631
Au	8.633774	9.969423	-4.699631
Au	10.072736	7.477067	-4.699631
Au	11.511698	4.984712	-4.699631
Au	12.950661	2.492356	-4.699631
Au	14.389623	0.000000	-4.699631
Au	-8.633774	34.892983	-4.699631
Au	-7.194811	32.400627	-4.699631
Au	-5.755849	29.908270	-4.699631
Au	-4.316887	27.415914	-4.699631
Au	-2.877925	24.923557	-4.699631
Au	-1.438962	22.431202	-4.699631
Au	0.000000	19.938847	-4.699631
Au	1.438962	17.446491	-4.699631
Au	2.877925	14.954135	-4.699631
Au	4.316887	12.461779	-4.699631
Au	5.755849	9.969423	-4.699631
Au	7.194811	7.477067	-4.699631
Au	8.633774	4.984712	-4.699631
Au	10.072736	2.492356	-4.699631
Au	11.511698	0.000000	-4.699631

Au	-11.511698	34.892983	-4.699631
Au	-10.072736	32.400627	-4.699631
Au	-8.633774	29.908270	-4.699631
Au	-7.194811	27.415914	-4.699631
Au	-5.755849	24.923557	-4.699631
Au	-4.316887	22.431202	-4.699631
Au	-2.877925	19.938847	-4.699631
Au	-1.438962	17.446491	-4.699631
Au	0.000000	14.954135	-4.699631
Au	1.438962	12.461779	-4.699631
Au	2.877925	9.969423	-4.699631
Au	4.316887	7.477067	-4.699631
Au	5.755849	4.984712	-4.699631
Au	7.194811	2.492356	-4.699631
Au	8.633774	0.000000	-4.699631
Au	-14.389623	34.892983	-4.699631
Au	-12.950661	32.400627	-4.699631
Au	-11.511698	29.908270	-4.699631
Au	-10.072736	27.415914	-4.699631
Au	-8.633774	24.923557	-4.699631
Au	-7.194811	22.431202	-4.699631
Au	-5.755849	19.938847	-4.699631
Au	-4.316887	17.446491	-4.699631
Au	-2.877925	14.954135	-4.699631
Au	-1.438962	12.461779	-4.699631
Au	0.000000	9.969423	-4.699631
Au	1.438962	7.477067	-4.699631
Au	2.877925	4.984712	-4.699631
Au	4.316887	2.492356	-4.699631
Au	5.755849	0.000000	-4.699631
Au	-17.267548	34.892983	-4.699631
Au	-15.828585	32.400627	-4.699631
Au	-14.389623	29.908270	-4.699631
Au	-12.950661	27.415914	-4.699631

Au	-11.511698	24.923557	-4.699631
Au	-10.072736	22.431202	-4.699631
Au	-8.633774	19.938847	-4.699631
Au	-7.194811	17.446491	-4.699631
Au	-5.755849	14.954135	-4.699631
Au	-4.316887	12.461779	-4.699631
Au	-2.877925	9.969423	-4.699631
Au	-1.438962	7.477067	-4.699631
Au	0.000000	4.984712	-4.699631
Au	1.438962	2.492356	-4.699631
Au	2.877925	0.000000	-4.699631
Au	-20.145472	34.892983	-4.699631
Au	-18.706511	32.400627	-4.699631
Au	-17.267548	29.908270	-4.699631
Au	-15.828585	27.415914	-4.699631
Au	-14.389623	24.923557	-4.699631
Au	-12.950661	22.431202	-4.699631
Au	-11.511698	19.938847	-4.699631
Au	-10.072736	17.446491	-4.699631
Au	-8.633774	14.954135	-4.699631
Au	-7.194811	12.461779	-4.699631
Au	-5.755849	9.969423	-4.699631
Au	-4.316887	7.477067	-4.699631
Au	-2.877925	4.984712	-4.699631
Au	-1.438962	2.492356	-4.699631
Au	0.000000	0.000000	-4.699631
