# Supplementary material for: On-Surface Synthesis of Azobenzene-Linked Porphyrin Derivatives
Source: J Phys Chem Lett. 2025 Nov 6;16(45):11833–41. doi: 10.1021/acs.jpclett.5c03174 (PMC12621240; doi:10.1021/acs.jpclett.5c03174)

checkCIF/PLATON report

You have not supplied any structure factors. As a result the full set of tests cannot be run.

THIS REPORT IS FOR GUIDANCE ONLY. IF USED AS PART OF A REVIEW PROCEDURE FOR PUBLICATION, IT SHOULD NOT REPLACE THE EXPERTISE OF AN EXPERIENCED CRYSTALLOGRAPHIC REFEREE.

No syntax errors found.   CIF dictionary   Interpreting this report

Datablock: jon1e_0m_pl

```
Bond precision:   C-C = 0.0048 A          Wavelength=0.71075

Cell:            a=10.5717(3)       b=16.0616(4)      c=19.8423(5)
                 alpha=74.638(1)    beta=83.107(1)    gamma=87.763(1)
Temperature:     110 K
```

	Calculated	Reported
Volume	3225.24(15)	3225.24(15)
Space group	P -1	P -1
Hall group	-P 1	-P 1
Moiety formula	C68 H75 N5 O2 Pt [+ solvent]	C68 H75 N5 O2 Pt
Sum formula	C68 H75 N5 O2 Pt [+ solvent]	C68 H75 N5 O2 Pt
Mr	1189.41	1189.42
Dx,g cm-3	1.225	1.225
Z	2	2
Mu (mm-1)	2.219	2.219
F000	1224.0	1224.0
F000'	1221.00	
h,k,lmax	13,20,24	13,20,24
Nref	13198	13125
Tmin,Tmax	0.821,0.877	0.671,0.980
Tmin'	0.521	

```
Correction method= # Reported T Limits: Tmin=0.671 Tmax=0.980
AbsCorr = MULTI-SCAN

Data completeness= 0.994          Theta(max)= 26.372
```

R(reflections)= 0.0316(12339)   wR2(reflections)= 0.0836(13125)

S = 1.063   Npar= 703

The following ALERTS were generated. Each ALERT has the format
test-name_ALERT_alert-type_alert-level.
Click on the hyperlinks for more details of the test.

Alert level C

```
ABSTY02_ALERT_1_C  An _exptl_absorpt_correction_type has been given without
            a literature citation. This should be contained in the
            _exptl_absorpt_process_details field.
            Absorption correction given as Multi-scan
PLAT094_ALERT_2_C Ratio of Maximum / Minimum Residual Density ....       3.40 Report
PLAT213_ALERT_2_C Atom O2               has ADP max/min Ratio .....       3.7 prolat
PLAT213_ALERT_2_C Atom C60              has ADP max/min Ratio .....       3.3 prolat
PLAT220_ALERT_2_C NonSolvent   Resd 1  C   Ueq(max)/Ueq(min) Range        5.4 Ratio
PLAT222_ALERT_3_C NonSolvent Resd 1  H   Uiso(max)/Uiso(min) Range        6.4 Ratio
PLAT242_ALERT_2_C Low    'MainMol' Ueq as Compared to Neighbors of         N5 Check
PLAT242_ALERT_2_C Low    'MainMol' Ueq as Compared to Neighbors of        C23 Check
PLAT242_ALERT_2_C Low    'MainMol' Ueq as Compared to Neighbors of        C57 Check
PLAT242_ALERT_2_C Low    'MainMol' Ueq as Compared to Neighbors of        C61 Check
PLAT790_ALERT_4_C Centre of Gravity not Within Unit Cell: Resd.  #          1 Note
              C68 H75 N5 O2 Pt
```

Alert level G

```
PLAT083_ALERT_2_G SHELXL Second Parameter in WGHT  Unusually Large       6.11 Why ?
PLAT154_ALERT_1_G The s.u.'s on the Cell Angles are Equal ..(Note)      0.001 Degree
PLAT606_ALERT_4_G Solvent Accessible VOID(S) in Structure ........          ! Info
PLAT794_ALERT_5_G Tentative Bond Valency for Pt        (II)      .       2.34 Info
PLAT868_ALERT_4_G ALERTS Due to the Use of _smtbx_masks Suppressed          ! Info
PLAT883_ALERT_1_G No Info/Value for _atom_sites_solution_primary .     Please Do !
PLAT933_ALERT_2_G Number of HKL-OMIT Records in Embedded .res File         17 Note
                0  1  0,    0 -1  1,    1  0  0,    0 -1  2,    0 -2  2,  -4 18  9,
               -2  2  1,    1  0  3,    1 30  0,    0  3  0,   -1 -1  1,   0  0  2,
                0 31  7,   -2  4  3,    2  1  5,   -3  2  0,   -1 27  1,
```

---

0 **ALERT level A** = Most likely a serious problem - resolve or explain
0 **ALERT level B** = A potentially serious problem, consider carefully
11 **ALERT level C** = Check. Ensure it is not caused by an omission or oversight
7 **ALERT level G** = General information/check it is not something unexpected

3 ALERT type 1 CIF construction/syntax error, inconsistent or missing data
10 ALERT type 2 Indicator that the structure model may be wrong or deficient
1 ALERT type 3 Indicator that the structure quality may be low
3 ALERT type 4 Improvement, methodology, query or suggestion
1 ALERT type 5 Informative message, check

Validation response form

Please find below a validation response form (VRF) that can be filled in and pasted into your CIF.

```
# start Validation Reply Form
_vrf_ABSTY02_jon1e_0m_pl
;
PROBLEM: An _exptl_absorpt_correction_type has been given without
RESPONSE: ...
;
_vrf_PLAT094_jon1e_0m_pl
;
PROBLEM: Ratio of Maximum / Minimum Residual Density ....       3.40 Report
RESPONSE: ...
;
_vrf_PLAT213_jon1e_0m_pl
;
PROBLEM: Atom O2              has ADP max/min Ratio .....        3.7 prolat
RESPONSE: ...
;
_vrf_PLAT220_jon1e_0m_pl
;
PROBLEM: NonSolvent   Resd 1  C   Ueq(max)/Ueq(min) Range        5.4 Ratio
RESPONSE: ...
;
_vrf_PLAT222_jon1e_0m_pl
;
PROBLEM: NonSolvent Resd 1  H   Uiso(max)/Uiso(min) Range        6.4 Ratio
RESPONSE: ...
;
_vrf_PLAT242_jon1e_0m_pl
;
PROBLEM: Low    'MainMol' Ueq as Compared to Neighbors of         N5 Check
RESPONSE: ...
;
_vrf_PLAT790_jon1e_0m_pl
;
PROBLEM: Centre of Gravity not Within Unit Cell: Resd.  #          1 Note
RESPONSE: ...
;
# end Validation Reply Form
```

It is advisable to attempt to resolve as many as possible of the alerts in all categories. Often the minor alerts point to easily fixed oversights, errors and omissions in your CIF or refinement strategy, so attention to these fine details can be worthwhile. In order to resolve some of the more serious problems it may be necessary to carry out additional measurements or structure refinements. However, the purpose of your study may justify the reported deviations and the more serious of these should normally be commented upon in the discussion or experimental section of a paper or in the "special_details" fields of the CIF. checkCIF was carefully designed to identify outliers and unusual parameters, but every test has its limitations and alerts that are not important in a particular case may appear. Conversely, the absence of alerts does not guarantee there are no aspects of the results needing attention. It is up to the individual to critically assess their own results and, if necessary, seek expert advice.

Publication of your CIF in IUCr journals

A basic structural check has been run on your CIF. These basic checks will be run on all CIFs submitted for publication in IUCr journals (*Acta Crystallographica*, *Journal of Applied Crystallography*, *Journal of Synchrotron Radiation*); however, if you intend to submit to *Acta Crystallographica Section C* or *E* or *IUCrData*, you should make sure that full publication checks are run on the final version of your CIF prior to submission.

Publication of your CIF in other journals

Please refer to the *Notes for Authors* of the relevant journal for any special instructions relating to CIF submission.

PLATON version of 06/01/2024; check.def file version of 05/01/2024

Datablock jon1e_0m_pl - ellipsoid plot

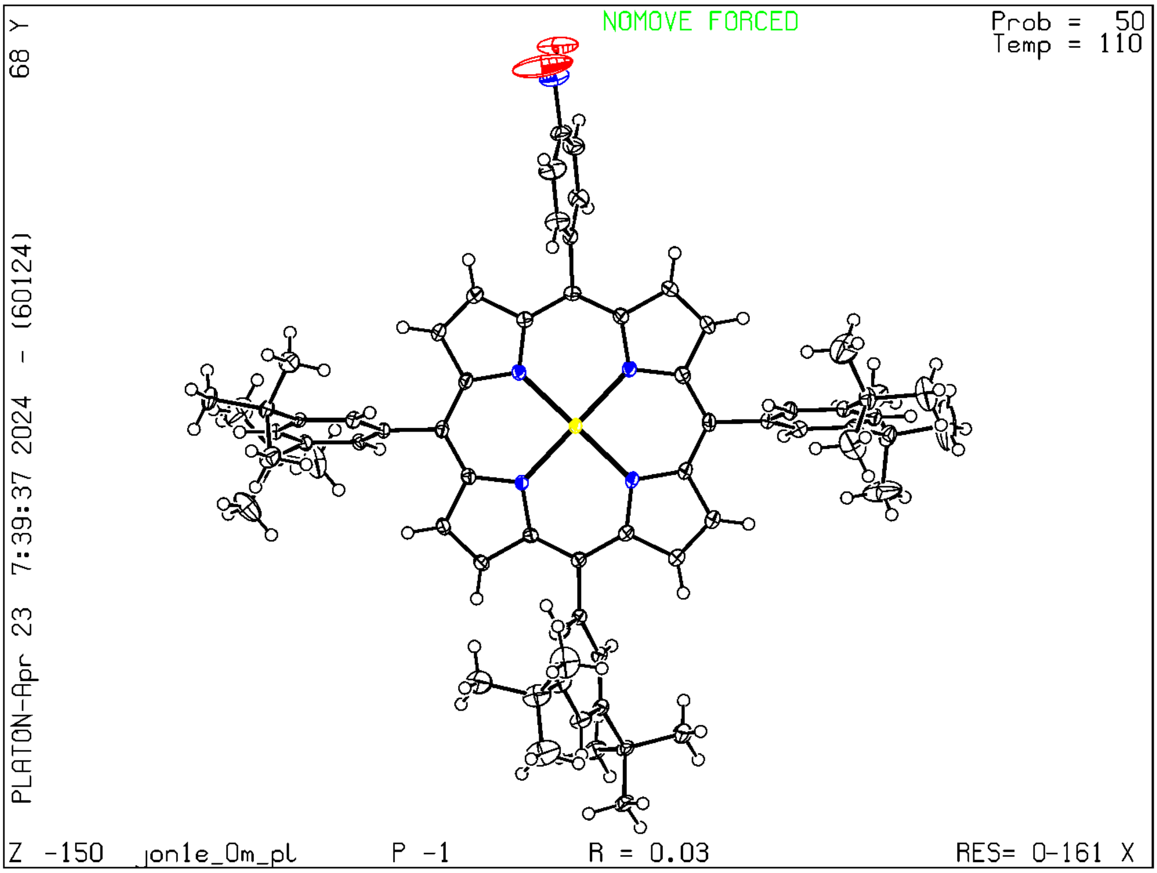

Supplement: Supplementary file 4 [file jz5c03174_si_004.pdf]
